# Supplementary figures and images for: IFN-β therapy rescues dysregulated IFN-stimulated proteins, serum cytokines, and neurotrophic factors in multiple sclerosis: Multiplex analysis of short-term and long-term IFN responses
Source: PLoS One. 2025 Sep 19;20(9):e0330867. doi: 10.1371/journal.pone.0330867 (PMC12449033; doi:10.1371/journal.pone.0330867)

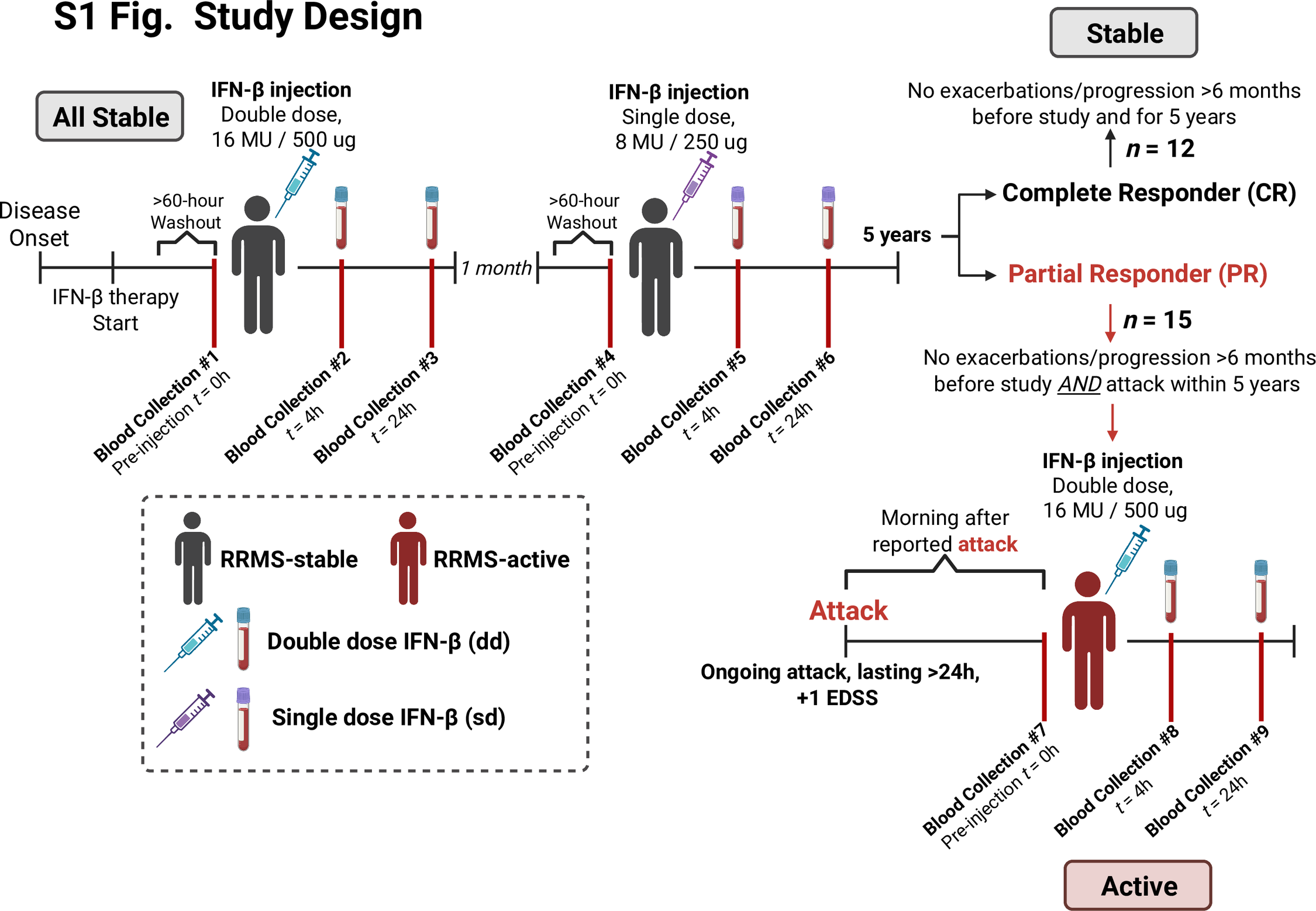

Supplement: S1 Fig — (TIF) [file pone.0330867.s001.tif]

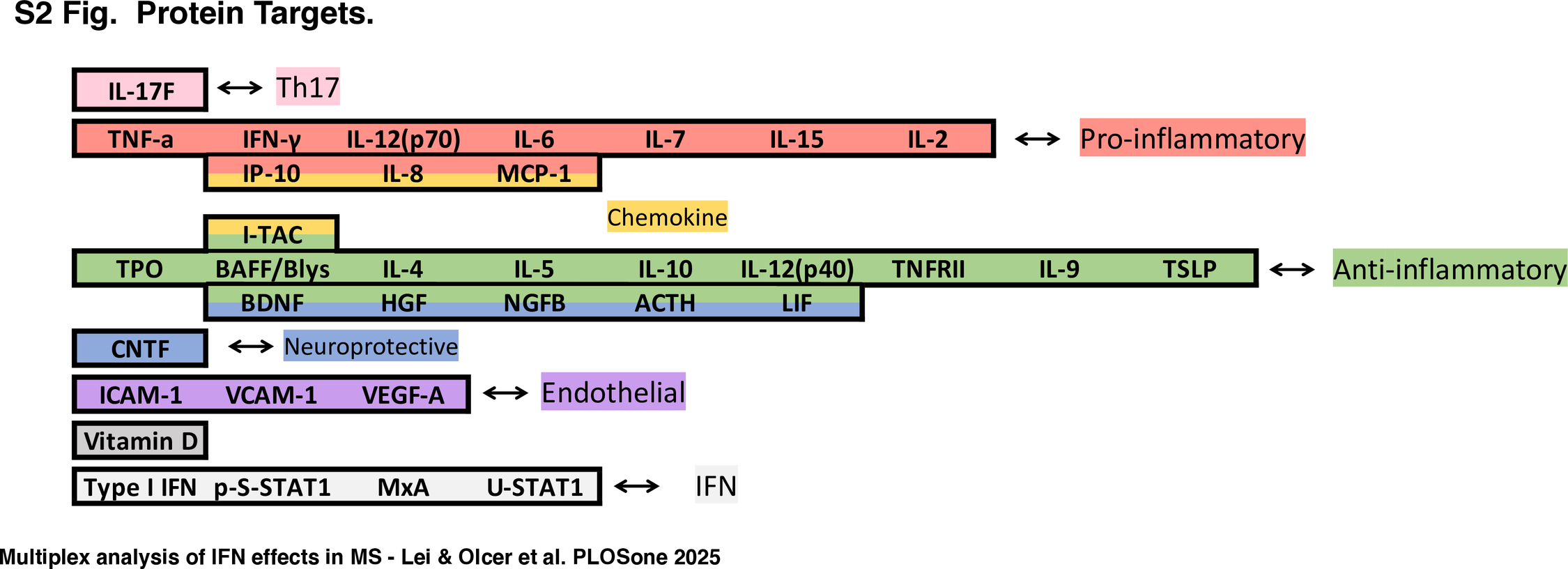

Supplement: S2 Fig — (TIF) [file pone.0330867.s002.tif]

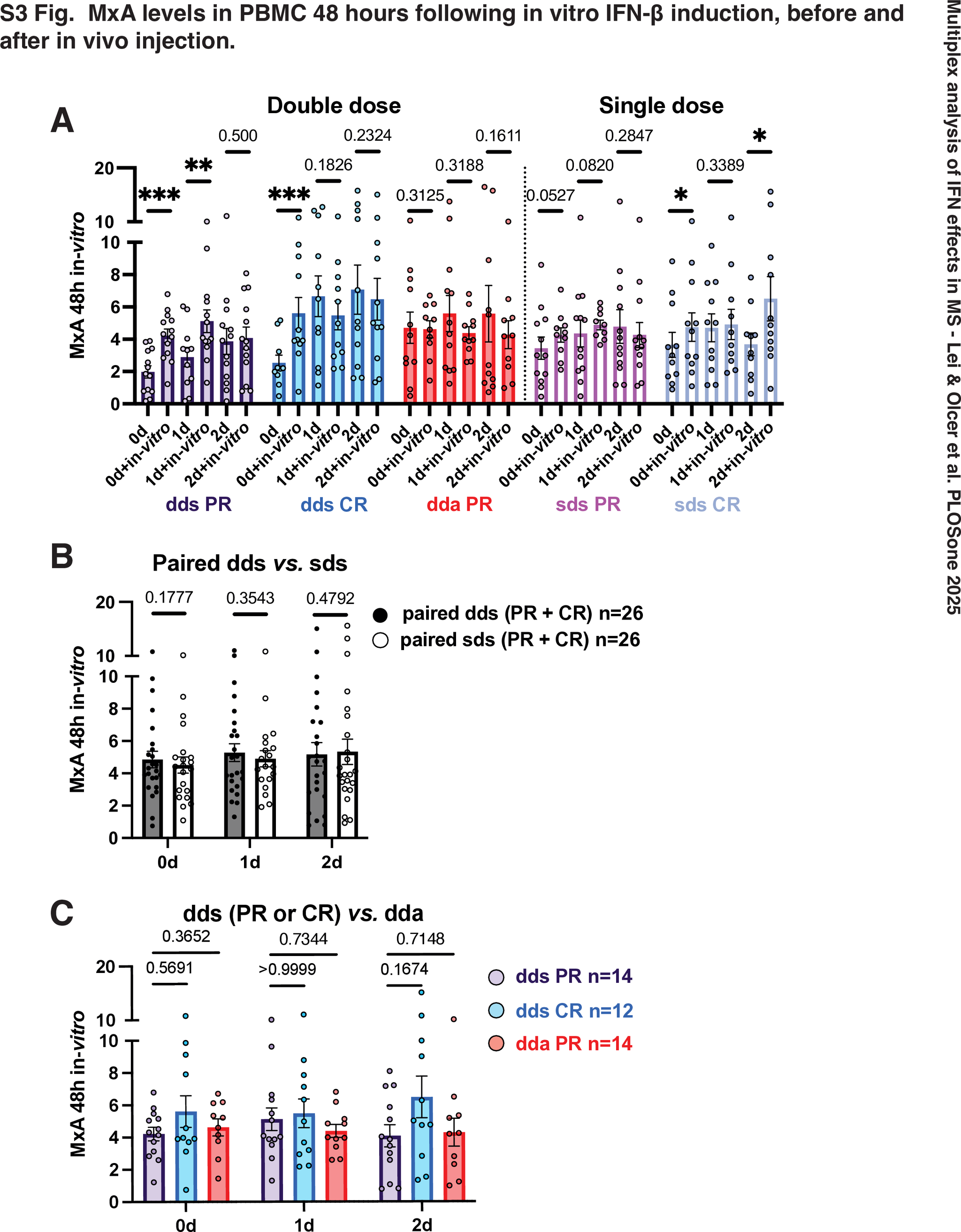

Supplement: S3 Fig — (TIF) [file pone.0330867.s005.tif]

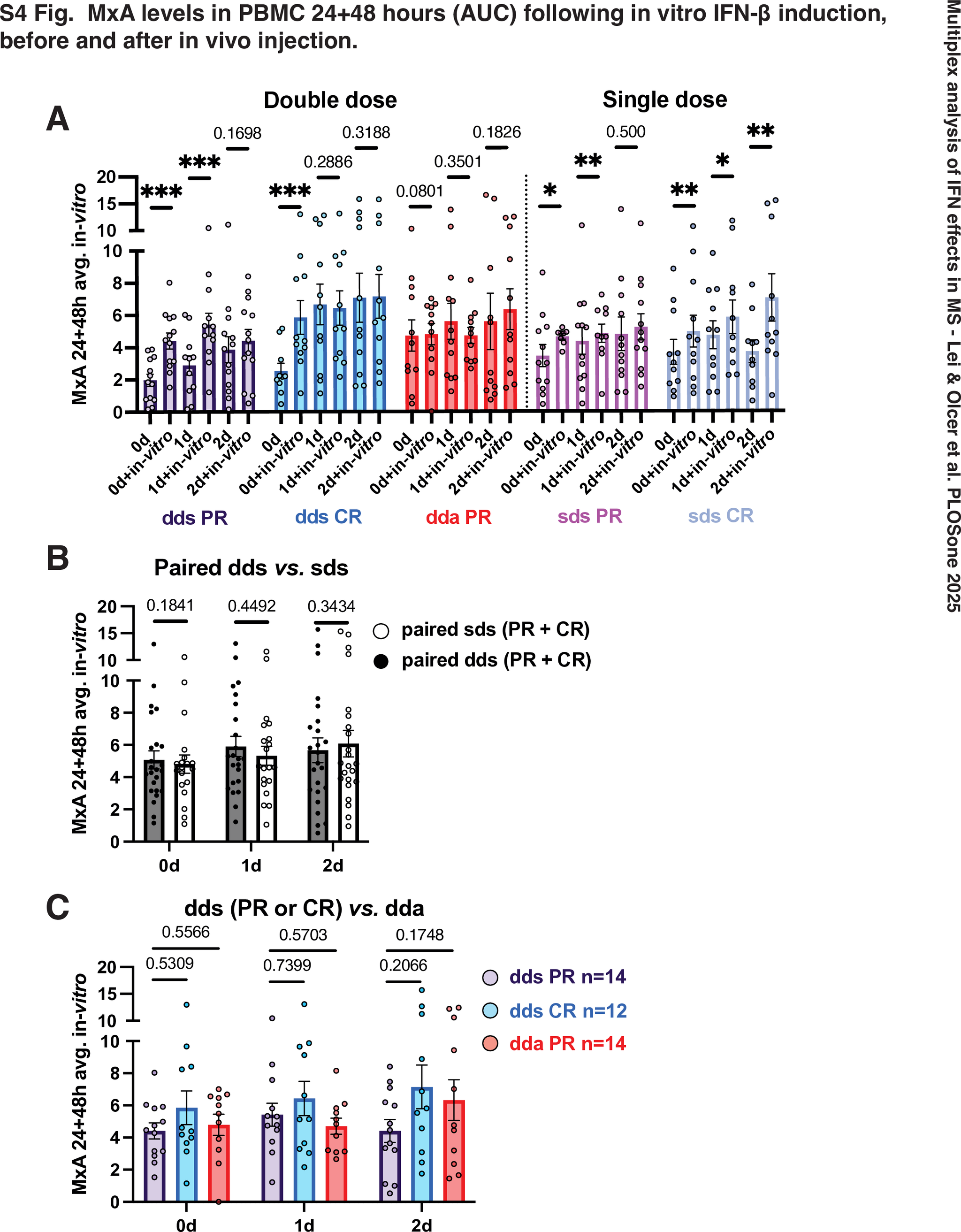

Supplement: S4 Fig — (TIF) [file pone.0330867.s006.tif]

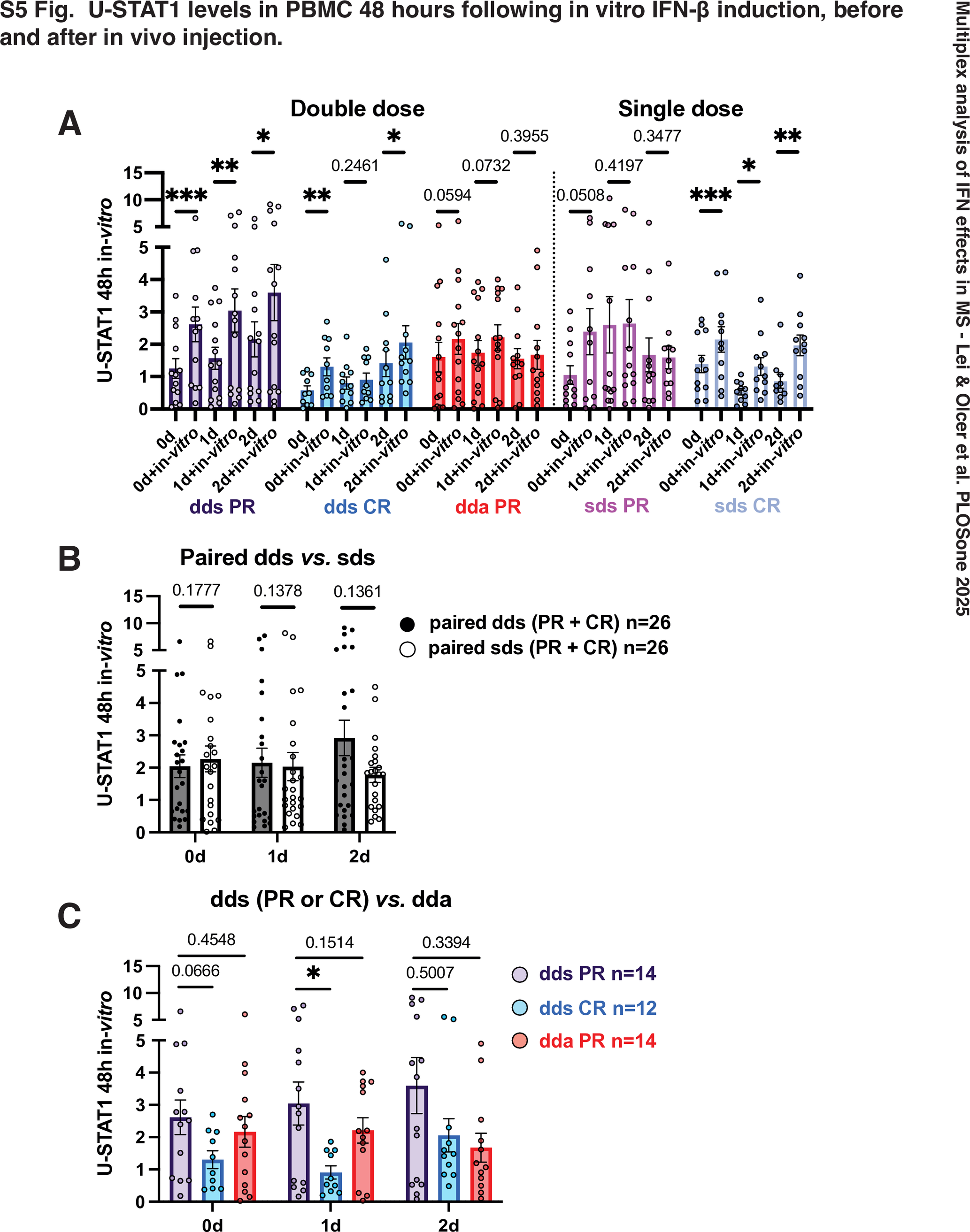

Supplement: S5 Fig — (TIF) [file pone.0330867.s007.tif]

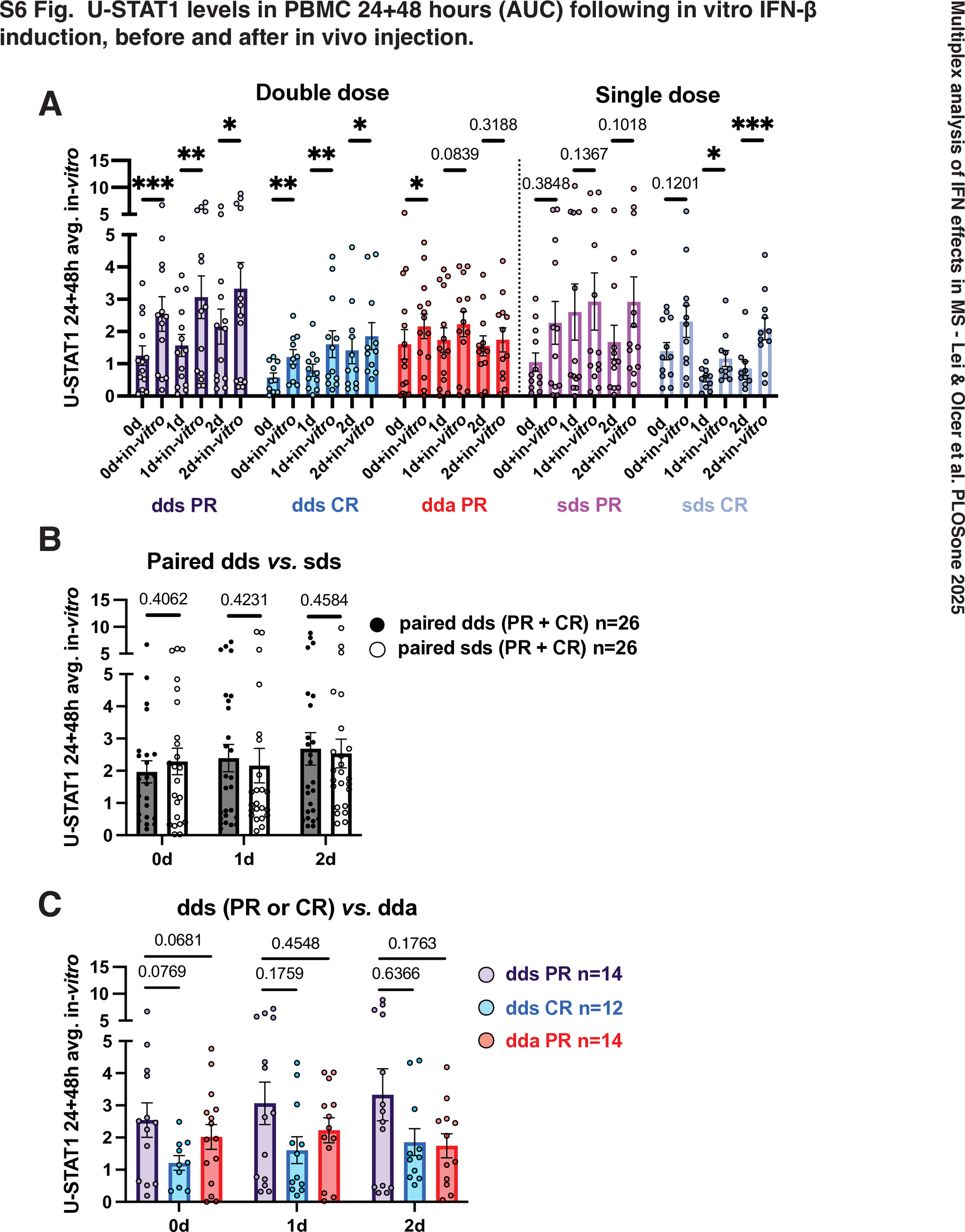

Supplement: S6 Fig — (TIF) [file pone.0330867.s008.tif]

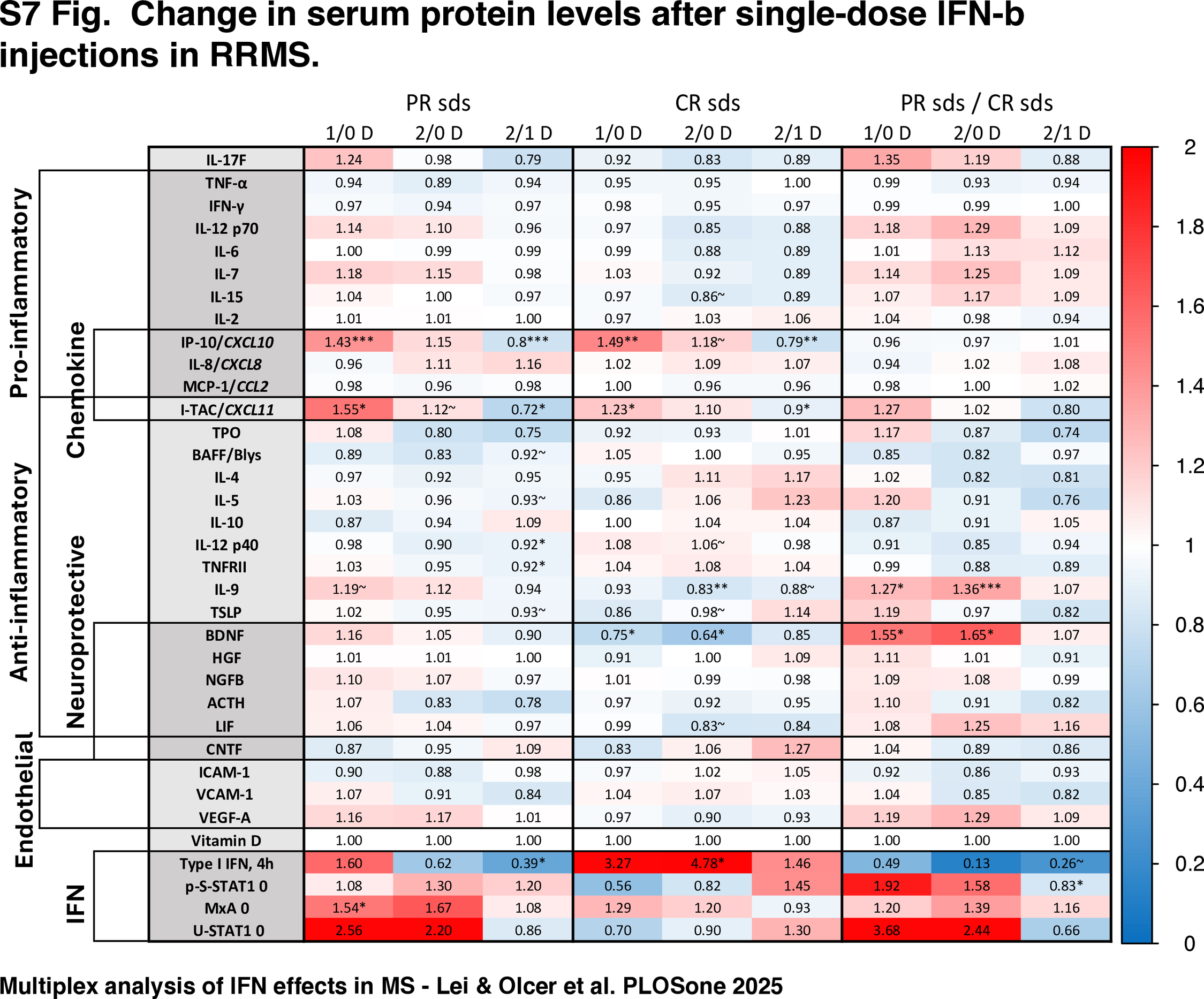

Supplement: S7 Fig — (TIF) [file pone.0330867.s009.tif]

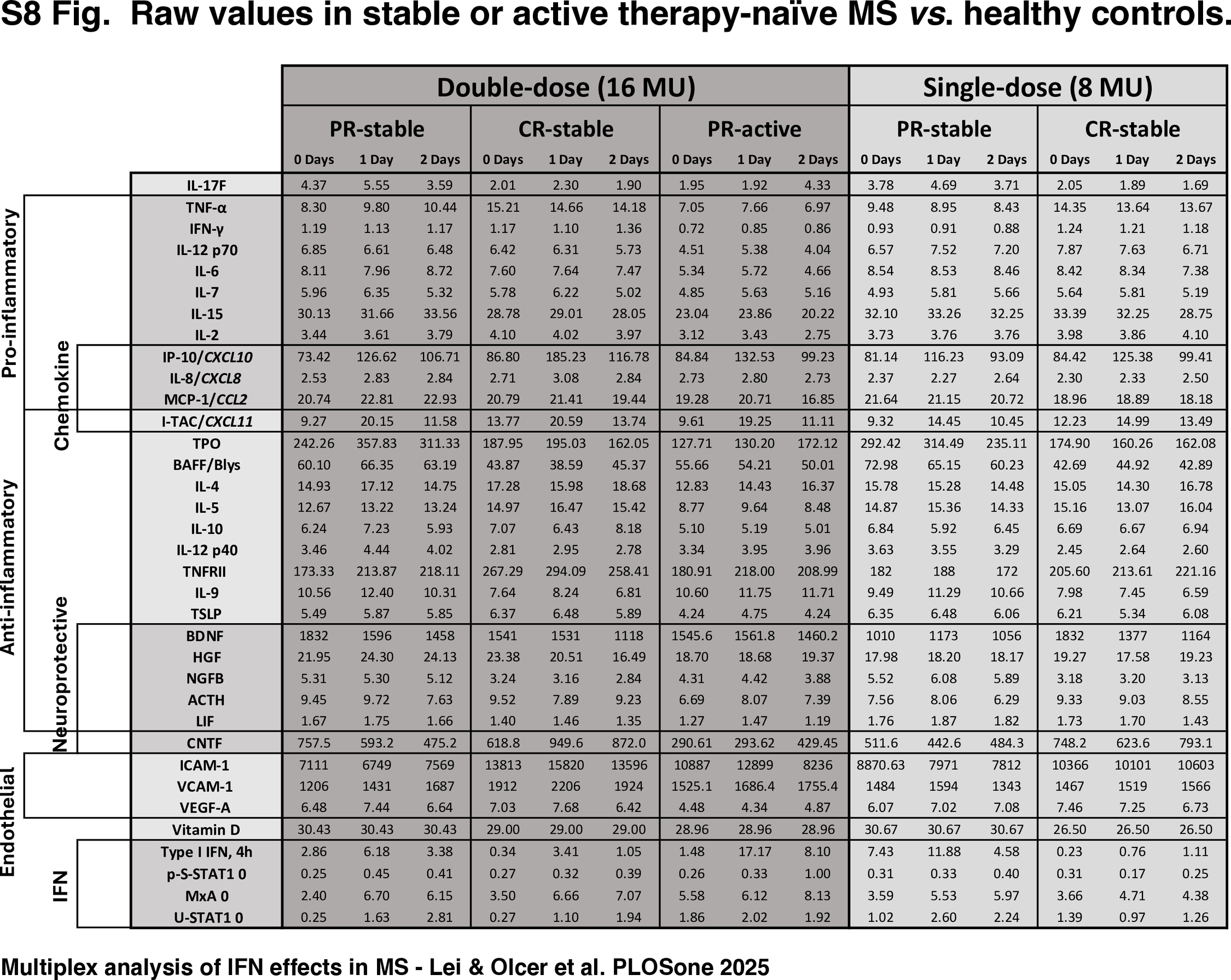

Supplement: S8 Fig — (TIF) [file pone.0330867.s010.tif]

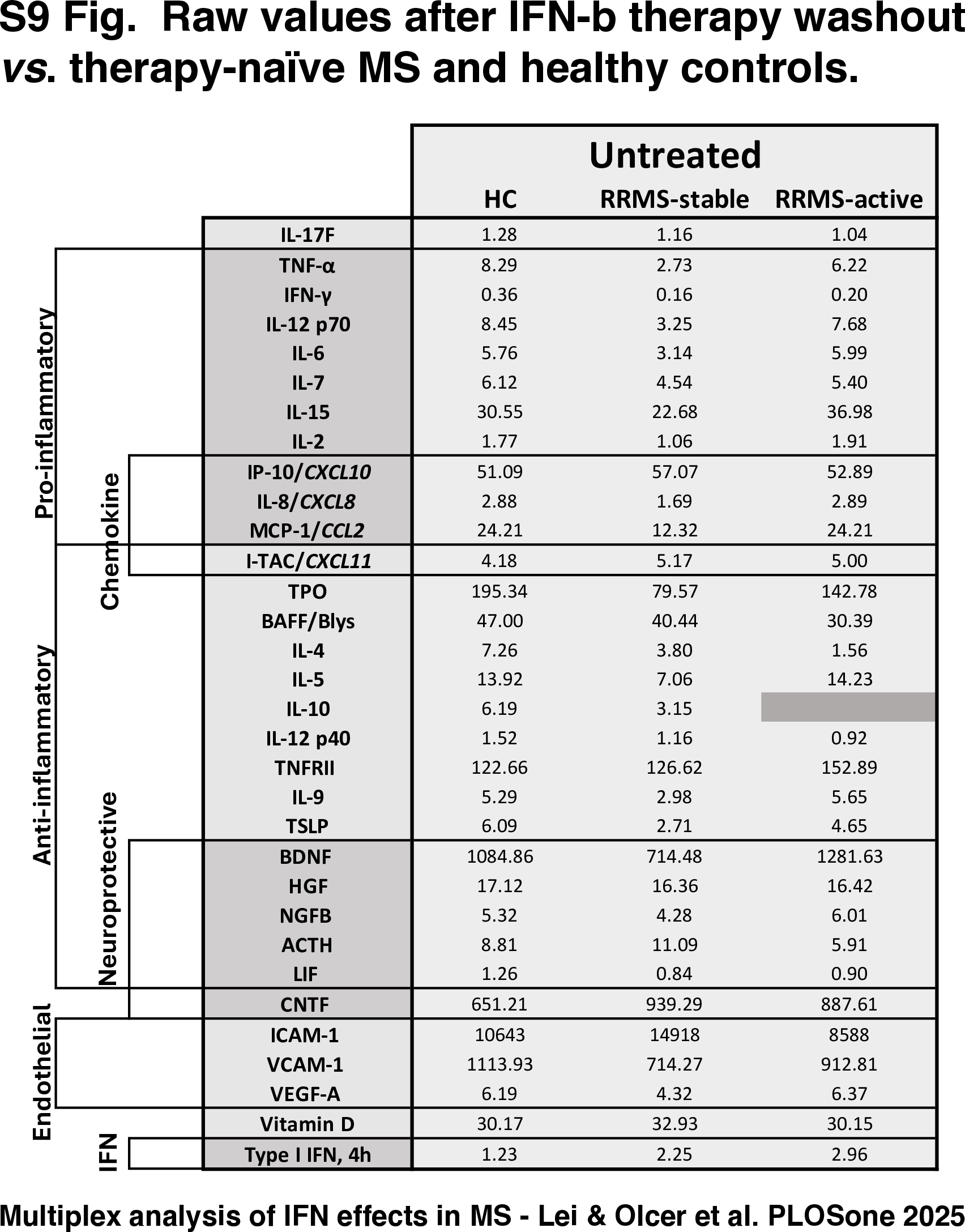

Supplement: S9 Fig — (TIF) [file pone.0330867.s011.tif]

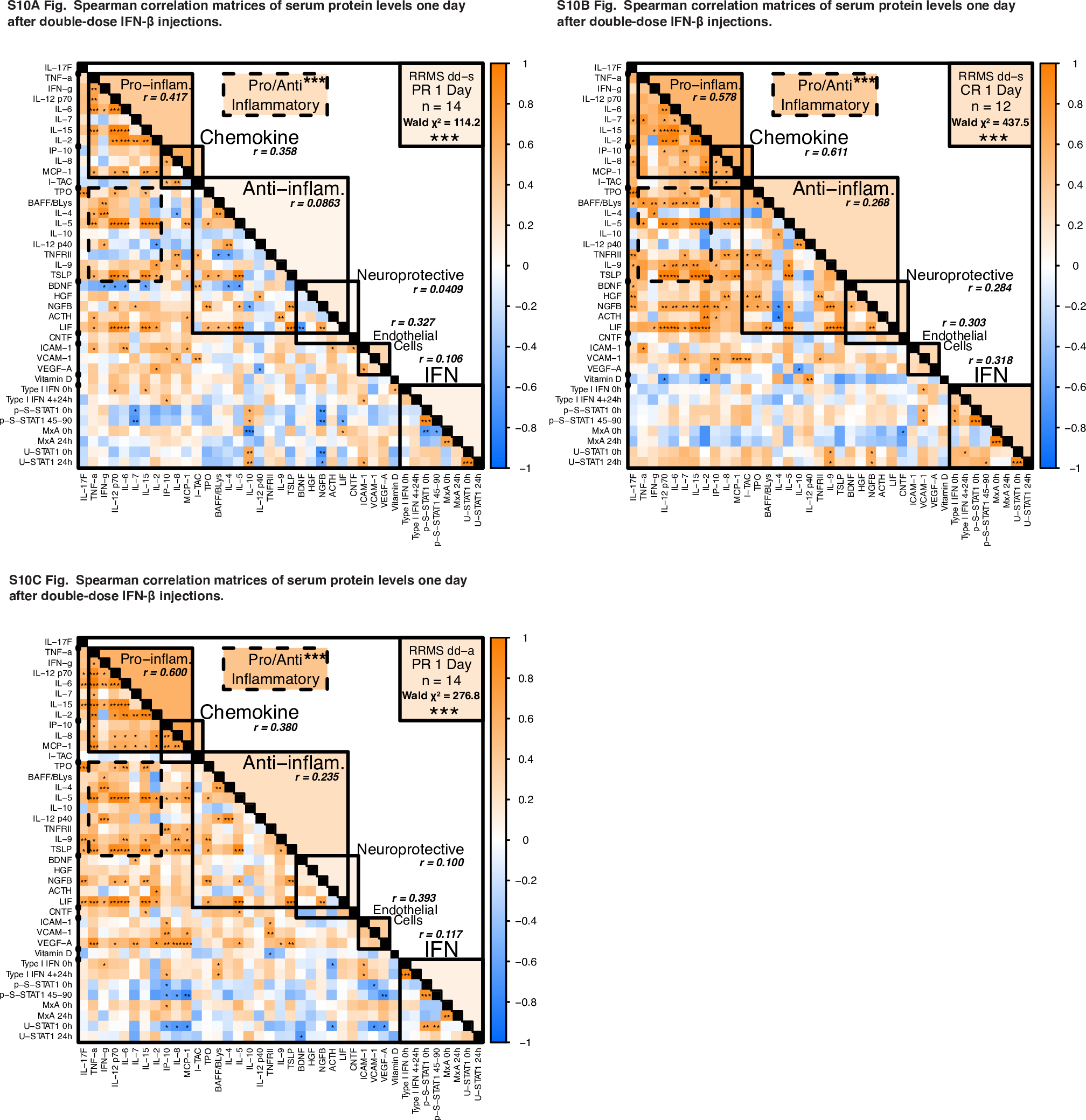

Supplement: S10 Fig — (TIF) [file pone.0330867.s012.tif]
