# Supplementary material for: IFN-β therapy rescues dysregulated IFN-stimulated proteins, serum cytokines, and neurotrophic factors in multiple sclerosis: Multiplex analysis of short-term and long-term IFN responses
Source: PLoS One. 2025 Sep 19;20(9):e0330867. doi: 10.1371/journal.pone.0330867 (PMC12449033; doi:10.1371/journal.pone.0330867)
Supplement: S11 Fig — (PDF) [file pone.0330867.s013.pdf]

p-S-STAT1

Subject #1

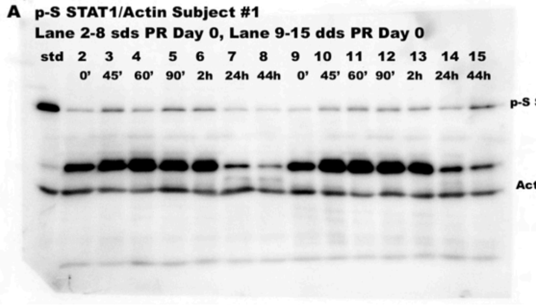

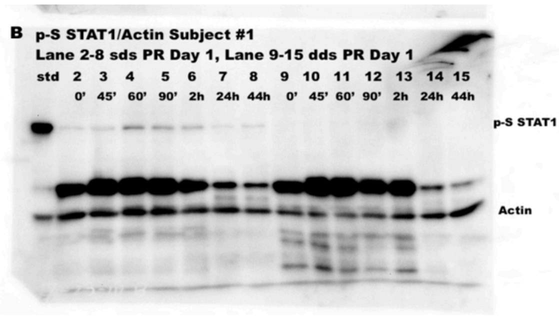

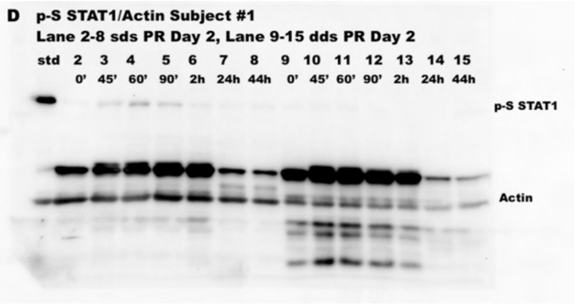

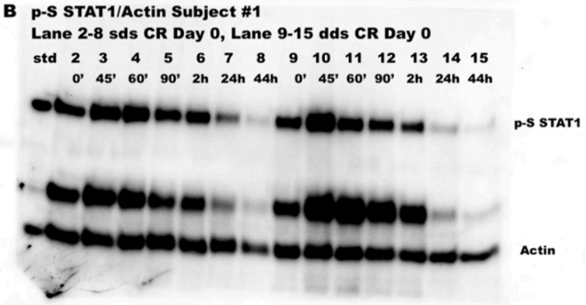

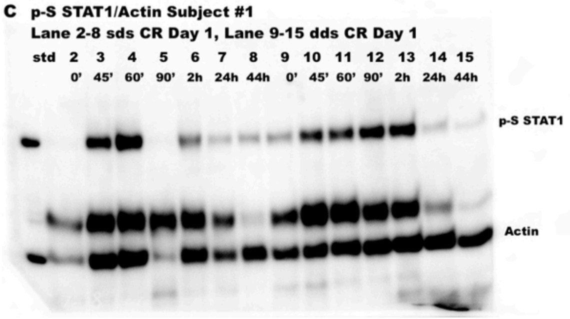

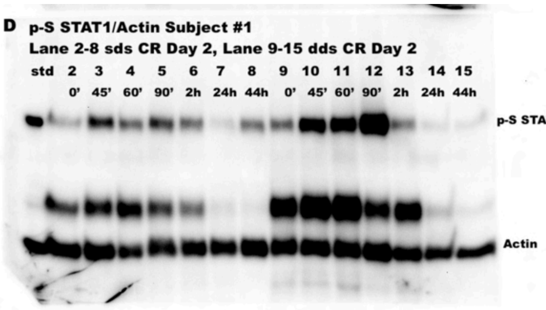

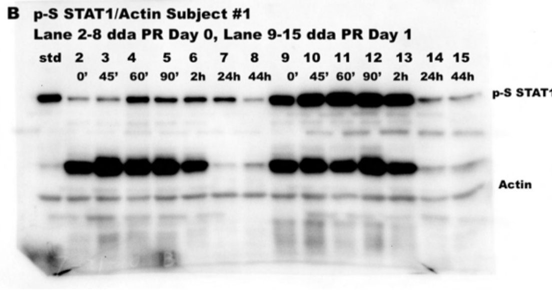

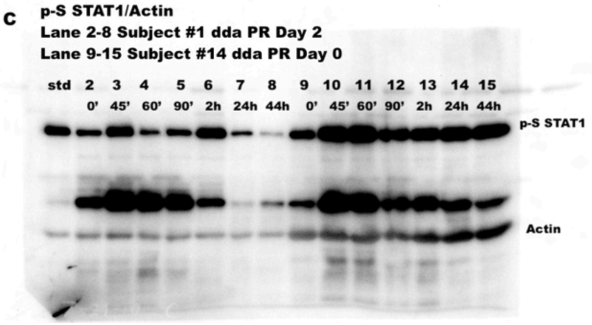

Subject #2

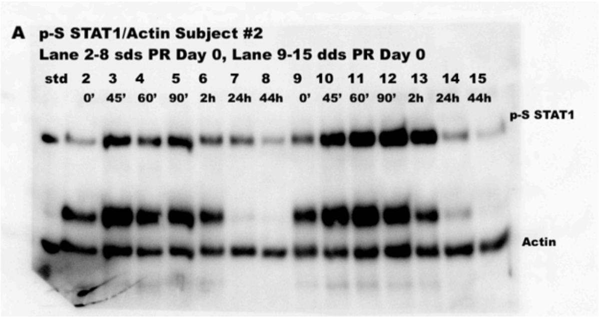
A
p-S STAT1/Actin Subject #2
Lane 2-8 sds PR Day 0, Lane 9-15 dds PR Day 0
std 2 3 4 5 6 7 8 9 10 11 12 13 14 15
0' 45' 60' 90' 2h 24h 44h 0' 45' 60' 90' 2h 24h 44h
p-S STAT1
Actin

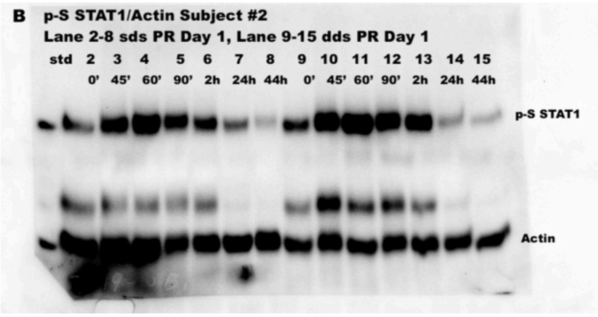
B
p-S STAT1/Actin Subject #2
Lane 2-8 sds PR Day 1, Lane 9-15 dds PR Day 1
std 2 3 4 5 6 7 8 9 10 11 12 13 14 15
0' 45' 60' 90' 2h 24h 44h 0' 45' 60' 90' 2h 24h 44h
p-S STAT1
Actin

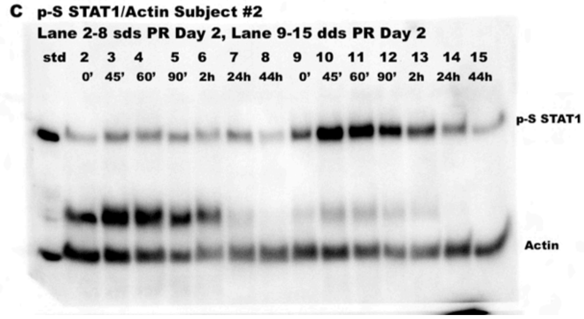
C
p-S STAT1/Actin Subject #2
Lane 2-8 sds PR Day 2, Lane 9-15 dds PR Day 2
std 2 3 4 5 6 7 8 9 10 11 12 13 14 15
0' 45' 60' 90' 2h 24h 44h 0' 45' 60' 90' 2h 24h 44h
p-S STAT1
Actin

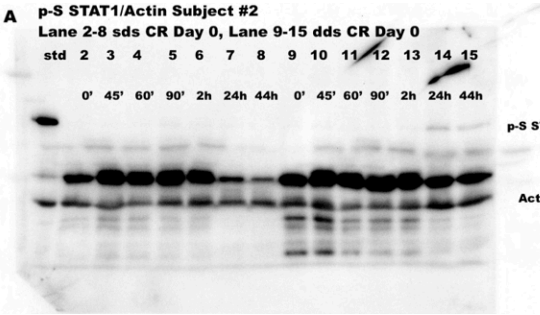
A
p-S STAT1/Actin Subject #2
Lane 2-8 sds CR Day 0, Lane 9-15 dds CR Day 0
std 2 3 4 5 6 7 8 9 10 11 12 13 14 15
0' 45' 60' 90' 2h 24h 44h 0' 45' 60' 90' 2h 24h 44h
p-S STAT1
Actin

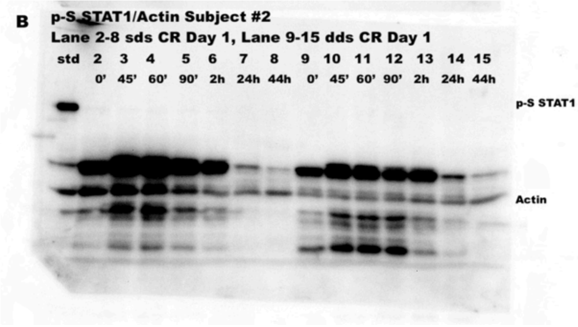
B
p-S STAT1/Actin Subject #2
Lane 2-8 sds CR Day 1, Lane 9-15 dds CR Day 1
std 2 3 4 5 6 7 8 9 10 11 12 13 14 15
0' 45' 60' 90' 2h 24h 44h 0' 45' 60' 90' 2h 24h 44h
p-S STAT1
Actin

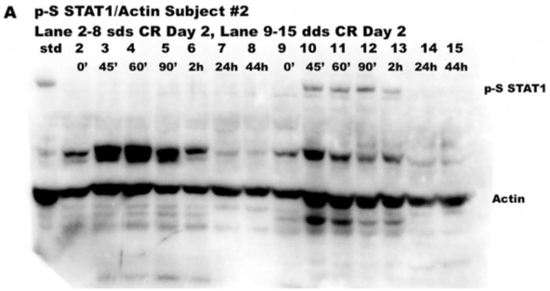
A
p-S STAT1/Actin Subject #2
Lane 2-8 sds CR Day 2, Lane 9-15 dds CR Day 2
std 2 3 4 5 6 7 8 9 10 11 12 13 14 15
0' 45' 60' 90' 2h 24h 44h 0' 45' 60' 90' 2h 24h 44h
p-S STAT1
Actin

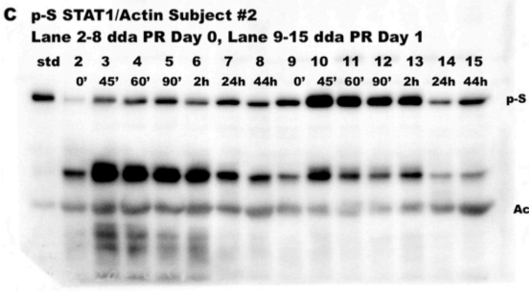
C
p-S STAT1/Actin Subject #2
Lane 2-8 dda PR Day 0, Lane 9-15 dda PR Day 1
std 2 3 4 5 6 7 8 9 10 11 12 13 14 15
0' 45' 60' 90' 2h 24h 44h 0' 45' 60' 90' 2h 24h 44h
p-S STAT1
Actin

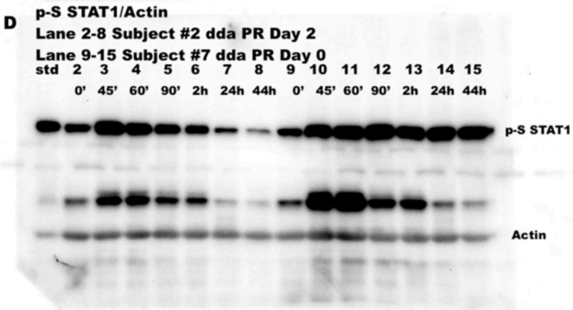
D
p-S STAT1/Actin
Lane 2-8 Subject #2 dda PR Day 2
Lane 9-15 Subject #7 dda PR Day 0
std 2 3 4 5 6 7 8 9 10 11 12 13 14 15
0' 45' 60' 90' 2h 24h 44h 0' 45' 60' 90' 2h 24h 44h
p-S STAT1
Actin

Subject #3

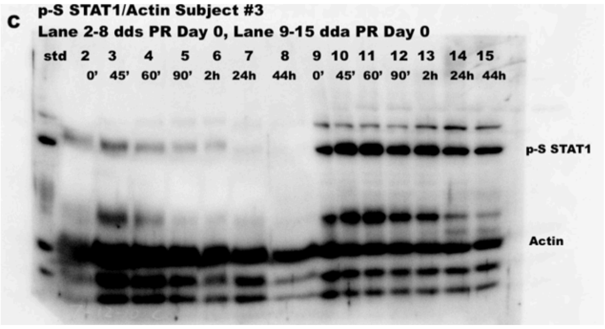
C
p-S STAT1/Actin Subject #3
Lane 2-8 dds PR Day 0, Lane 9-15 dda PR Day 0
std 2 3 4 5 6 7 8 9 10 11 12 13 14 15
0' 45' 60' 90' 2h 24h 44h 0' 45' 60' 90' 2h 24h 44h
p-S STAT1
Actin

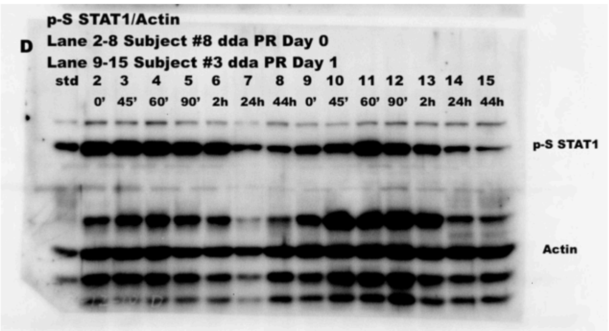
p-S STAT1/Actin
D
Lane 2-8 Subject #8 dda PR Day 0
Lane 9-15 Subject #3 dda PR Day 1
std 2 3 4 5 6 7 8 9 10 11 12 13 14 15
0' 45' 60' 90' 2h 24h 44h 0' 45' 60' 90' 2h 24h 44h
p-S STAT1
Actin

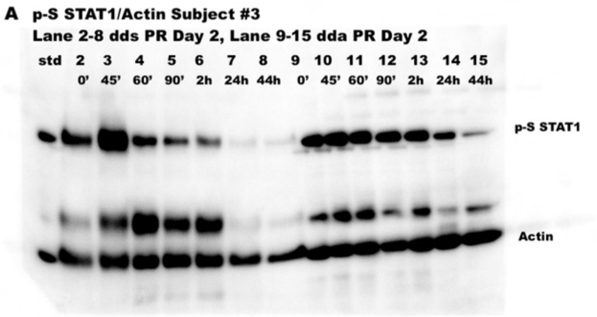
A
p-S STAT1/Actin Subject #3
Lane 2-8 dds PR Day 2, Lane 9-15 dda PR Day 2
std 2 3 4 5 6 7 8 9 10 11 12 13 14 15
0' 45' 60' 90' 2h 24h 44h 0' 45' 60' 90' 2h 24h 44h
p-S STAT1
Actin

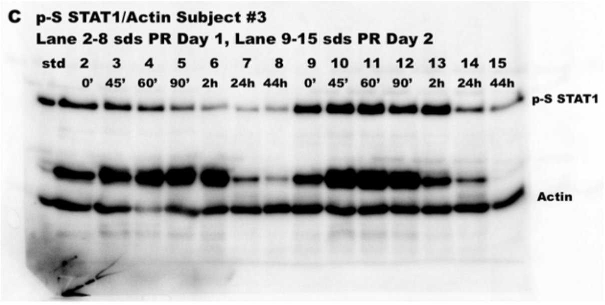
C
p-S STAT1/Actin Subject #3
Lane 2-8 sds PR Day 1, Lane 9-15 sds PR Day 2
std 2 3 4 5 6 7 8 9 10 11 12 13 14 15
0' 45' 60' 90' 2h 24h 44h 0' 45' 60' 90' 2h 24h 44h
p-S STAT1
Actin

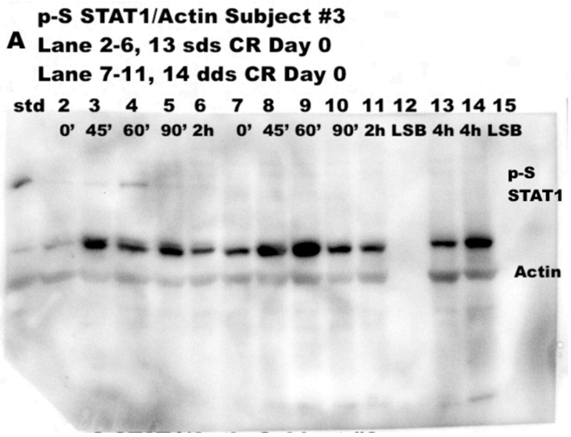
p-S STAT1/Actin Subject #3
A
Lane 2-6, 13 sds CR Day 0
Lane 7-11, 14 dds CR Day 0
std 2 3 4 5 6 7 8 9 10 11 12 13 14 15
0' 45' 60' 90' 2h 0' 45' 60' 90' 2h LSB 4h 4h LSB
p-S
STAT1
Actin

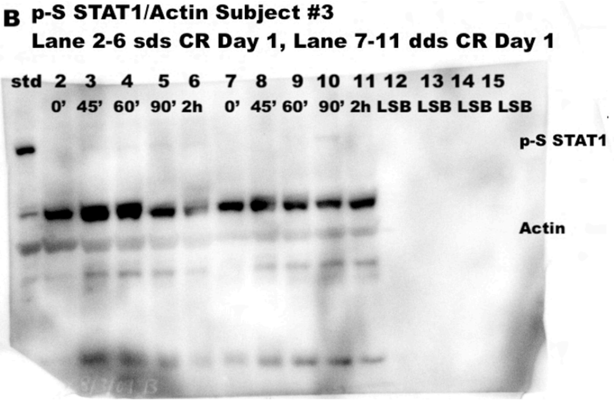
B
p-S STAT1/Actin Subject #3
Lane 2-6 sds CR Day 1, Lane 7-11 dds CR Day 1
std 2 3 4 5 6 7 8 9 10 11 12 13 14 15
0' 45' 60' 90' 2h 0' 45' 60' 90' 2h LSB LSB LSB LSB
p-S STAT1
Actin

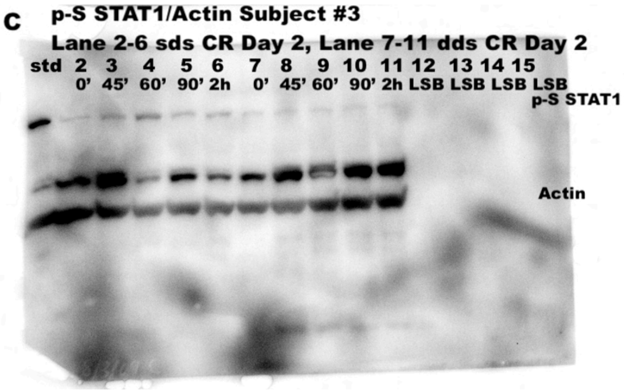
C
p-S STAT1/Actin Subject #3
Lane 2-6 sds CR Day 2, Lane 7-11 dds CR Day 2
std 2 3 4 5 6 7 8 9 10 11 12 13 14 15
0' 45' 60' 90' 2h 0' 45' 60' 90' 2h LSB LSB LSB LSB
p-S STAT1
Actin

Subject #4

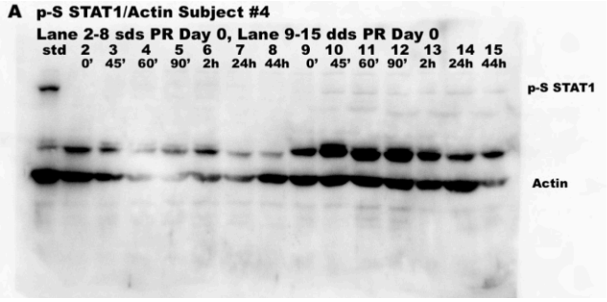

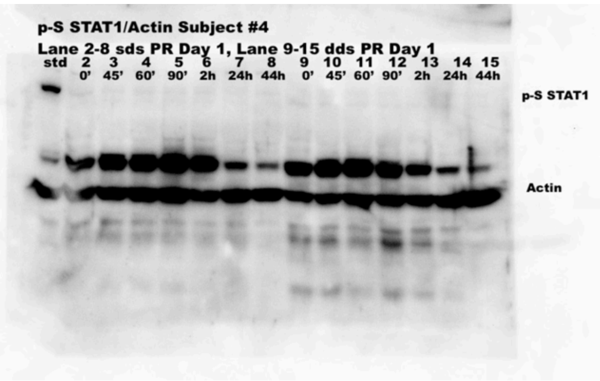

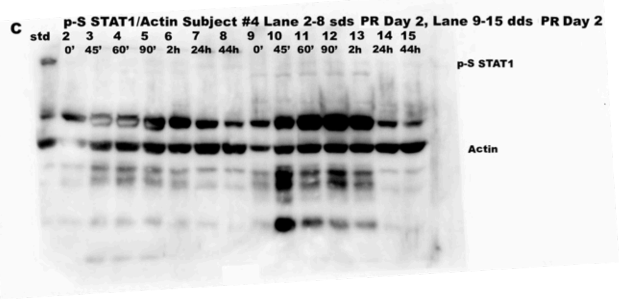

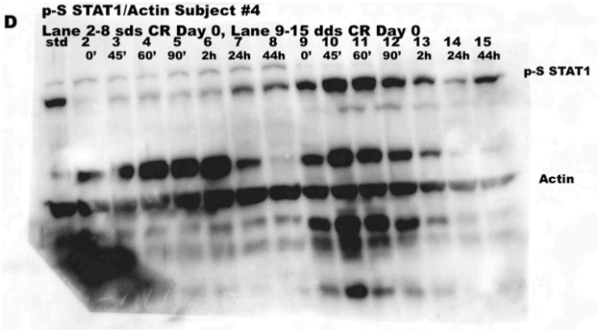

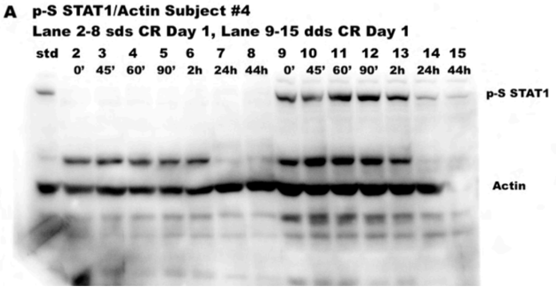

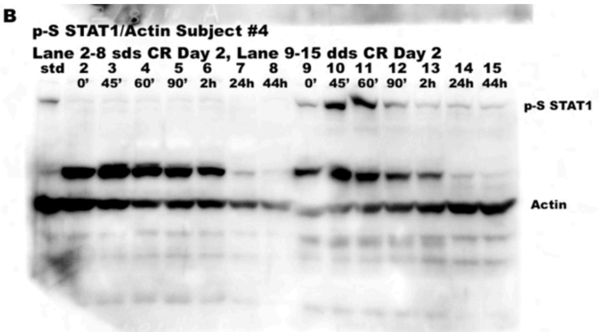

Subject #5

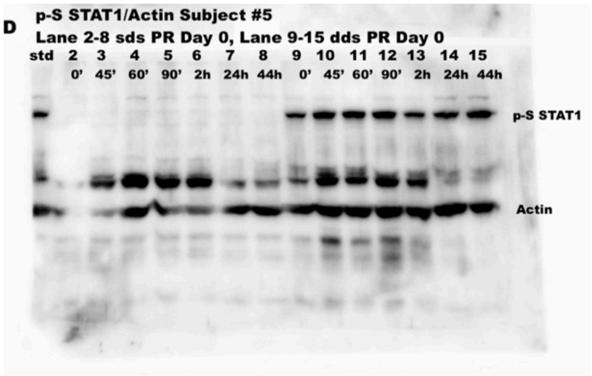

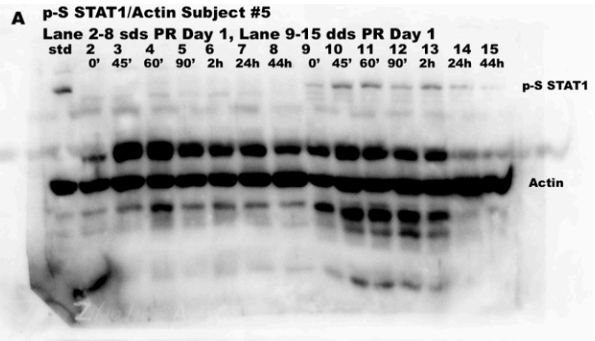

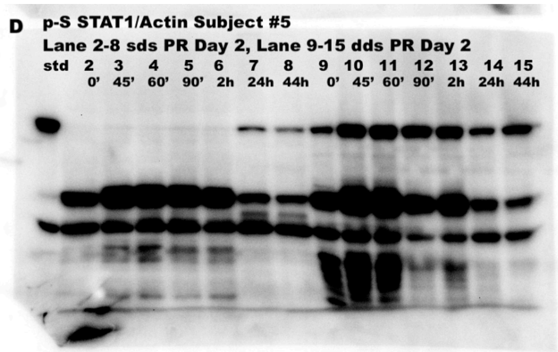
D p-S STAT1/Actin Subject #5
Lane 2-8 sds PR Day 2, Lane 9-15 dds PR Day 2
std 2 3 4 5 6 7 8 9 10 11 12 13 14 15
0' 45' 60' 90' 2h 24h 44h 0' 45' 60' 90' 2h 24h 44h
p-S STAT1
Actin

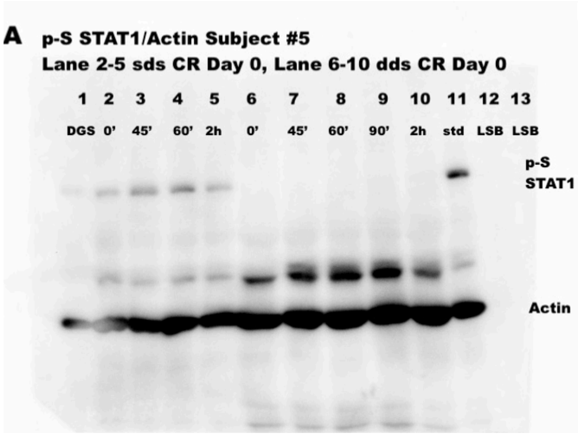
A p-S STAT1/Actin Subject #5
Lane 2-5 sds CR Day 0, Lane 6-10 dds CR Day 0
1 2 3 4 5 6 7 8 9 10 11 12 13
DGS 0' 45' 60' 2h 0' 45' 60' 90' 2h std LSB LSB
p-S STAT1
Actin

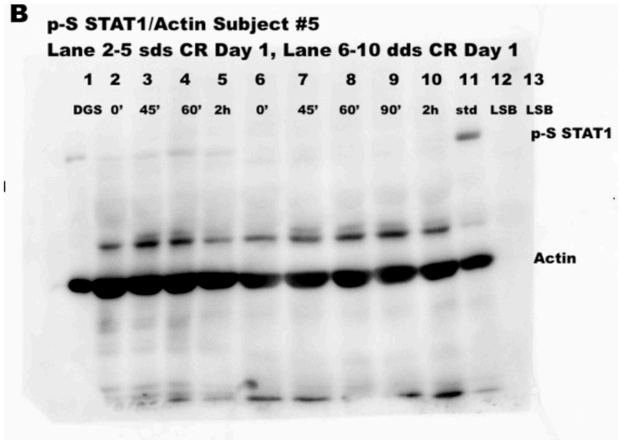
B p-S STAT1/Actin Subject #5
Lane 2-5 sds CR Day 1, Lane 6-10 dds CR Day 1
1 2 3 4 5 6 7 8 9 10 11 12 13
DGS 0' 45' 60' 2h 0' 45' 60' 90' 2h std LSB LSB
p-S STAT1
Actin

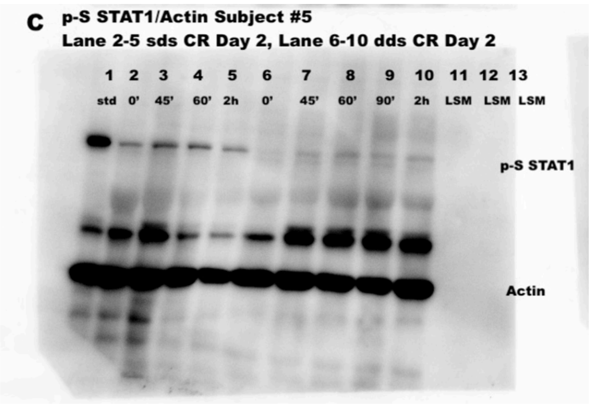
C p-S STAT1/Actin Subject #5
Lane 2-5 sds CR Day 2, Lane 6-10 dds CR Day 2
1 2 3 4 5 6 7 8 9 10 11 12 13
std 0' 45' 60' 2h 0' 45' 60' 90' 2h LSM LSM LSM
p-S STAT1
Actin

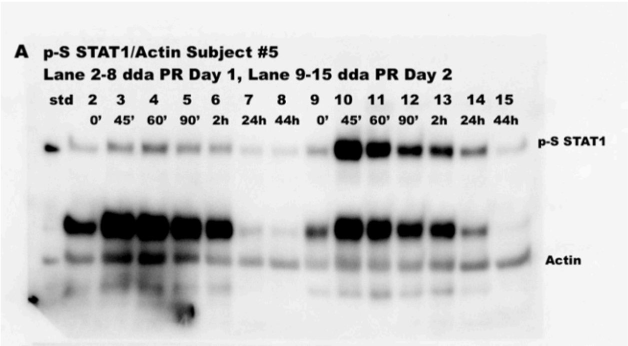
A p-S STAT1/Actin Subject #5
Lane 2-8 dda PR Day 1, Lane 9-15 dda PR Day 2
std 2 3 4 5 6 7 8 9 10 11 12 13 14 15
0' 45' 60' 90' 2h 24h 44h 0' 45' 60' 90' 2h 24h 44h
p-S STAT1
Actin

Subject #6

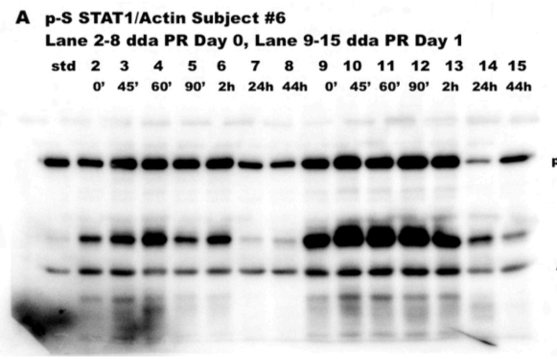
A p-S STAT1/Actin Subject #6
Lane 2-8 dda PR Day 0, Lane 9-15 dda PR Day 1
std 2 3 4 5 6 7 8 9 10 11 12 13 14 15
0' 45' 60' 90' 2h 24h 44h 0' 45' 60' 90' 2h 24h 44h
p-S STAT1
Actin

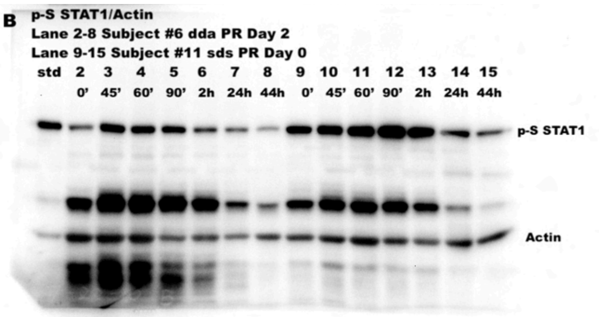
B p-S STAT1/Actin
Lane 2-8 Subject #6 dda PR Day 2
Lane 9-15 Subject #11 sds PR Day 0
std 2 3 4 5 6 7 8 9 10 11 12 13 14 15
0' 45' 60' 90' 2h 24h 44h 0' 45' 60' 90' 2h 24h 44h
p-S STAT1
Actin

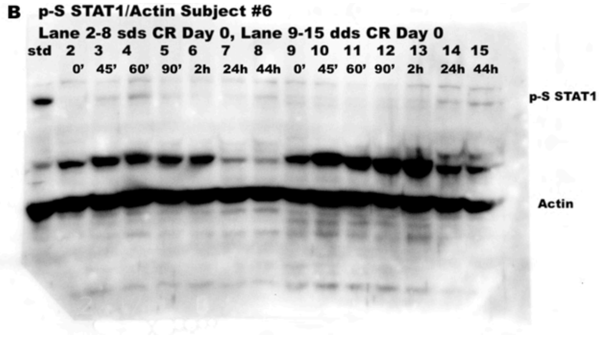
B p-S STAT1/Actin Subject #6
Lane 2-8 sds CR Day 0, Lane 9-15 dds CR Day 0
std 2 3 4 5 6 7 8 9 10 11 12 13 14 15
0' 45' 60' 90' 2h 24h 44h 0' 45' 60' 90' 2h 24h 44h
p-S STAT1
Actin

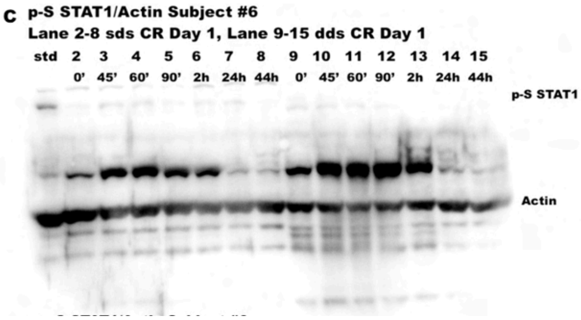
C p-S STAT1/Actin Subject #6
Lane 2-8 sds CR Day 1, Lane 9-15 dds CR Day 1
std 2 3 4 5 6 7 8 9 10 11 12 13 14 15
0' 45' 60' 90' 2h 24h 44h 0' 45' 60' 90' 2h 24h 44h
p-S STAT1
Actin

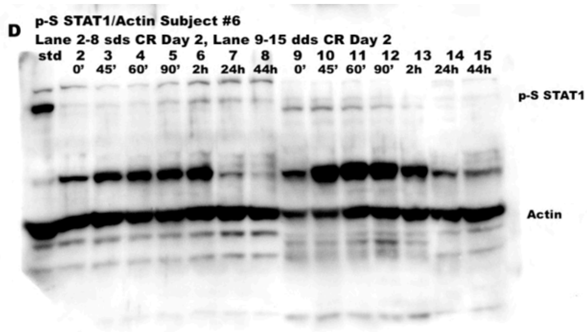
D p-S STAT1/Actin Subject #6
Lane 2-8 sds CR Day 2, Lane 9-15 dds CR Day 2
std 2 3 4 5 6 7 8 9 10 11 12 13 14 15
0' 45' 60' 90' 2h 24h 44h 0' 45' 60' 90' 2h 24h 44h
p-S STAT1
Actin

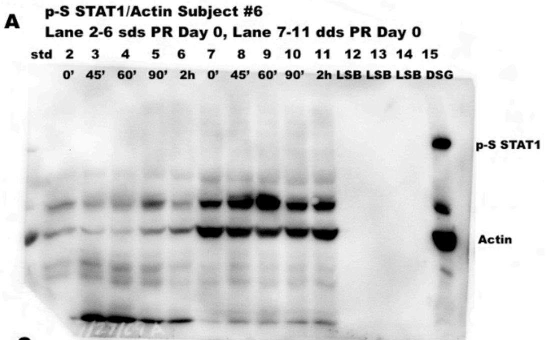
A p-S STAT1/Actin Subject #6
Lane 2-6 sds PR Day 0, Lane 7-11 dds PR Day 0
std 2 3 4 5 6 7 8 9 10 11 12 13 14 15
0' 45' 60' 90' 2h 0' 45' 60' 90' 2h LSB LSB LSB DSG
p-S STAT1
Actin

Subject #7

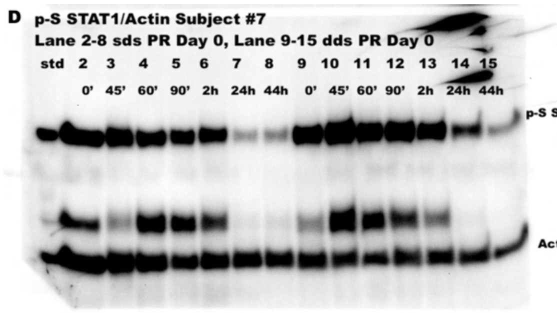
D p-S STAT1/Actin Subject #7
Lane 2-8 sds PR Day 0, Lane 9-15 dds PR Day 0
std 2 3 4 5 6 7 8 9 10 11 12 13 14 15
0' 45' 60' 90' 2h 24h 44h 0' 45' 60' 90' 2h 24h 44h
p-S STAT1
Actin

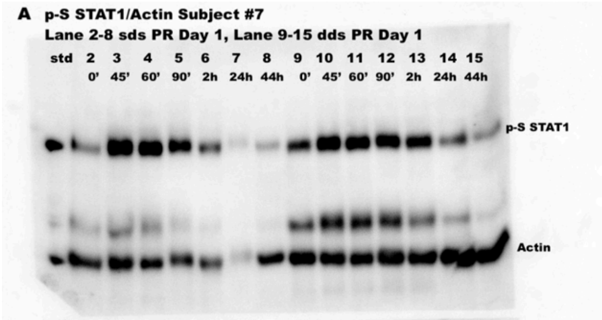
A p-S STAT1/Actin Subject #7
Lane 2-8 sds PR Day 1, Lane 9-15 dds PR Day 1
std 2 3 4 5 6 7 8 9 10 11 12 13 14 15
0' 45' 60' 90' 2h 24h 44h 0' 45' 60' 90' 2h 24h 44h
p-S STAT1
Actin

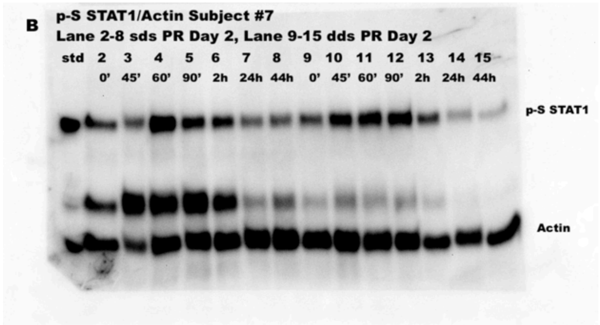
B p-S STAT1/Actin Subject #7
Lane 2-8 sds PR Day 2, Lane 9-15 dds PR Day 2
std 2 3 4 5 6 7 8 9 10 11 12 13 14 15
0' 45' 60' 90' 2h 24h 44h 0' 45' 60' 90' 2h 24h 44h
p-S STAT1
Actin

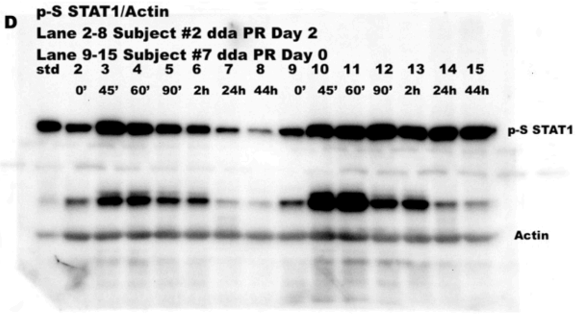
D p-S STAT1/Actin
Lane 2-8 Subject #2 dda PR Day 2
Lane 9-15 Subject #7 dda PR Day 0
std 2 3 4 5 6 7 8 9 10 11 12 13 14 15
0' 45' 60' 90' 2h 24h 44h 0' 45' 60' 90' 2h 24h 44h
p-S STAT1
Actin

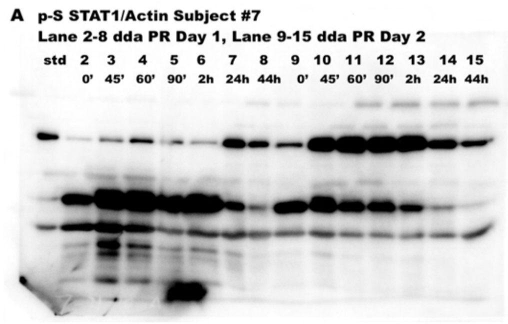
A p-S STAT1/Actin Subject #7
Lane 2-8 dda PR Day 1, Lane 9-15 dda PR Day 2
std 2 3 4 5 6 7 8 9 10 11 12 13 14 15
0' 45' 60' 90' 2h 24h 44h 0' 45' 60' 90' 2h 24h 44h
p-S STAT1
Actin

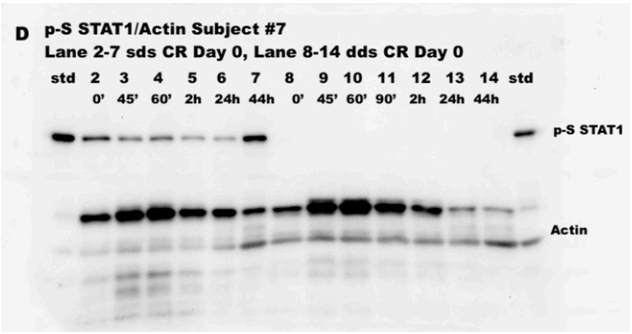
D p-S STAT1/Actin Subject #7
Lane 2-7 sds CR Day 0, Lane 8-14 dds CR Day 0
std 2 3 4 5 6 7 8 9 10 11 12 13 14 std
0' 45' 60' 2h 24h 44h 0' 45' 60' 90' 2h 24h 44h
p-S STAT1
Actin

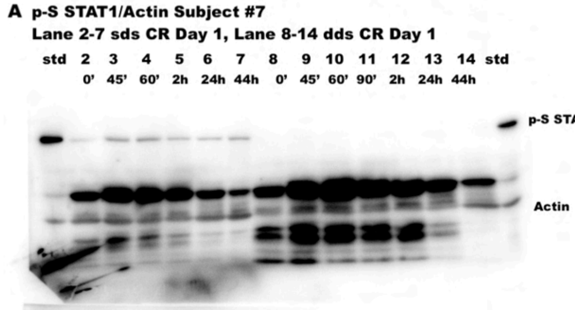
A p-S STAT1/Actin Subject #7
Lane 2-7 sds CR Day 1, Lane 8-14 dds CR Day 1
std 2 3 4 5 6 7 8 9 10 11 12 13 14 std
0' 45' 60' 2h 24h 44h 0' 45' 60' 90' 2h 24h 44h
p-S STAT1
Actin

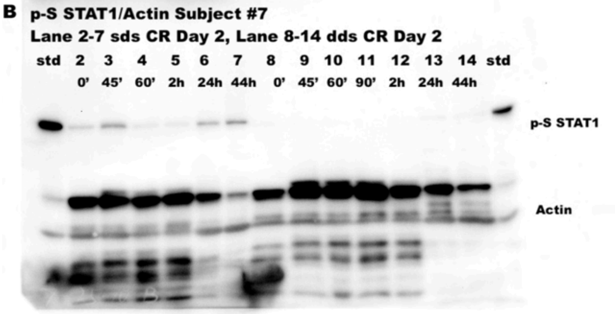
B p-S STAT1/Actin Subject #7
Lane 2-7 sds CR Day 2, Lane 8-14 dds CR Day 2
std 2 3 4 5 6 7 8 9 10 11 12 13 14 std
0' 45' 60' 2h 24h 44h 0' 45' 60' 90' 2h 24h 44h
p-S STAT1
Actin

Subject #8

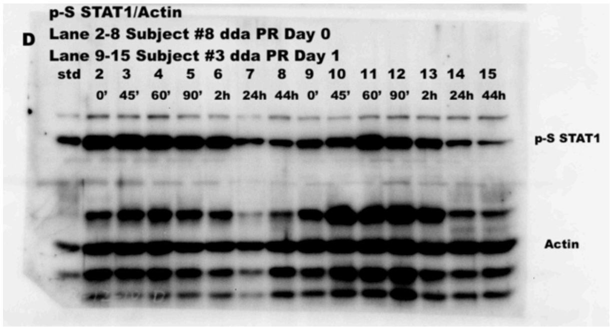
p-S STAT1/Actin
D Lane 2-8 Subject #8 dda PR Day 0
Lane 9-15 Subject #3 dda PR Day 1
std 2 3 4 5 6 7 8 9 10 11 12 13 14 15
0' 45' 60' 90' 2h 24h 44h 0' 45' 60' 90' 2h 24h 44h
p-S STAT1
Actin

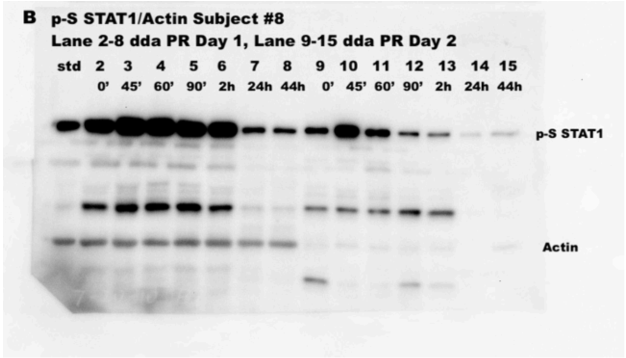
B p-S STAT1/Actin Subject #8
Lane 2-8 dda PR Day 1, Lane 9-15 dda PR Day 2
std 2 3 4 5 6 7 8 9 10 11 12 13 14 15
0' 45' 60' 90' 2h 24h 44h 0' 45' 60' 90' 2h 24h 44h
p-S STAT1
Actin

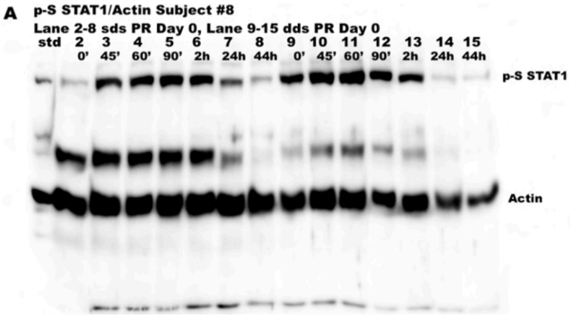
A p-S STAT1/Actin Subject #8
Lane 2-8 sds PR Day 0, Lane 9-15 dds PR Day 0
std 2 3 4 5 6 7 8 9 10 11 12 13 14 15
0' 45' 60' 90' 2h 24h 44h 0' 45' 60' 90' 2h 24h 44h
p-S STAT1
Actin

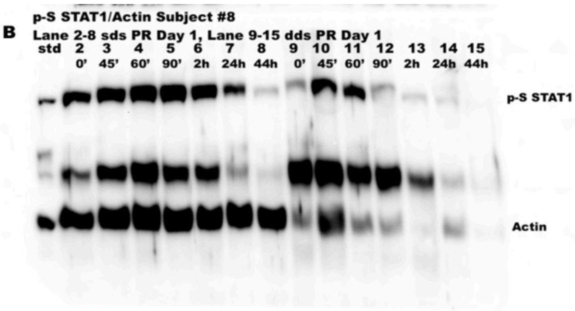
p-S STAT1/Actin Subject #8
B Lane 2-8 sds PR Day 1, Lane 9-15 dds PR Day 1
std 2 3 4 5 6 7 8 9 10 11 12 13 14 15
0' 45' 60' 90' 2h 24h 44h 0' 45' 60' 90' 2h 24h 44h
p-S STAT1
Actin

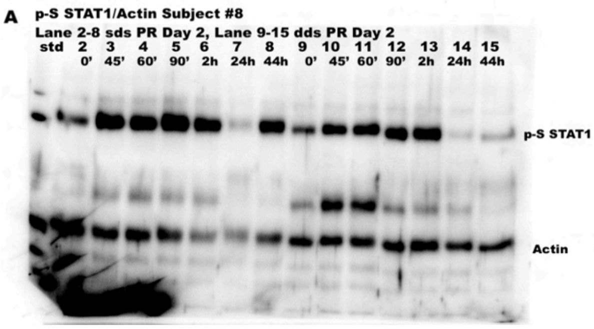
A p-S STAT1/Actin Subject #8
Lane 2-8 sds PR Day 2, Lane 9-15 dds PR Day 2
std 2 3 4 5 6 7 8 9 10 11 12 13 14 15
0' 45' 60' 90' 2h 24h 44h 0' 45' 60' 90' 2h 24h 44h
p-S STAT1
Actin

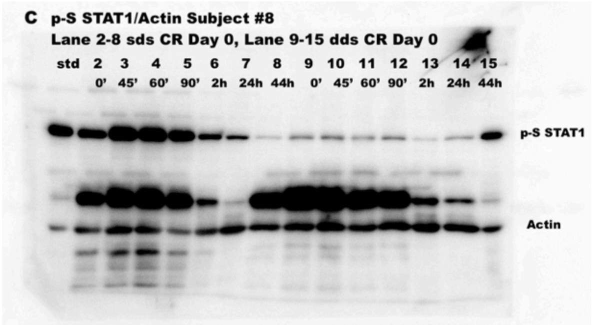
C p-S STAT1/Actin Subject #8
Lane 2-8 sds CR Day 0, Lane 9-15 dds CR Day 0
std 2 3 4 5 6 7 8 9 10 11 12 13 14 15
0' 45' 60' 90' 2h 24h 44h 0' 45' 60' 90' 2h 24h 44h
p-S STAT1
Actin

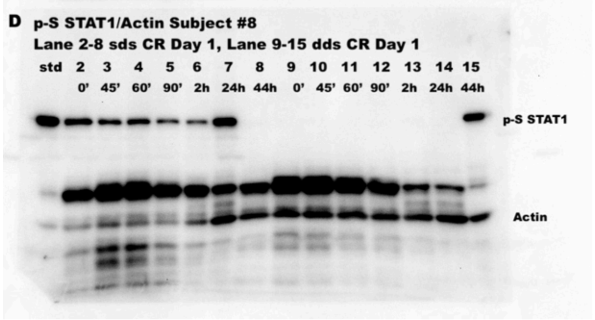
D p-S STAT1/Actin Subject #8
Lane 2-8 sds CR Day 1, Lane 9-15 dds CR Day 1
std 2 3 4 5 6 7 8 9 10 11 12 13 14 15
0' 45' 60' 90' 2h 24h 44h 0' 45' 60' 90' 2h 24h 44h
p-S STAT1
Actin

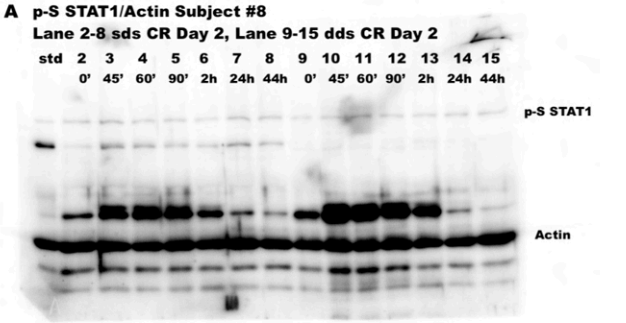
A p-S STAT1/Actin Subject #8
Lane 2-8 sds CR Day 2, Lane 9-15 dds CR Day 2
std 2 3 4 5 6 7 8 9 10 11 12 13 14 15
0' 45' 60' 90' 2h 24h 44h 0' 45' 60' 90' 2h 24h 44h
p-S STAT1
Actin

Subject #9

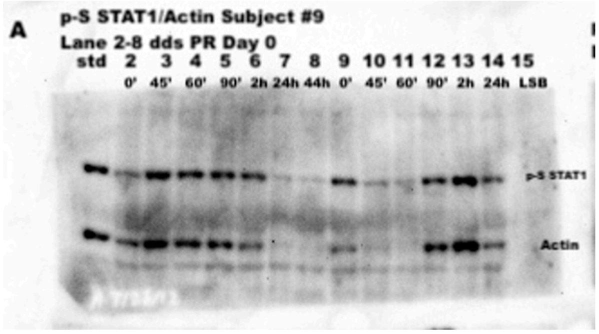
A p-S STAT1/Actin Subject #9
Lane 2-8 dds PR Day 0
std 2 3 4 5 6 7 8 9 10 11 12 13 14 15
0' 45' 60' 90' 2h 24h 44h 0' 45' 60' 90' 2h 24h LSB
p-S STAT1
Actin

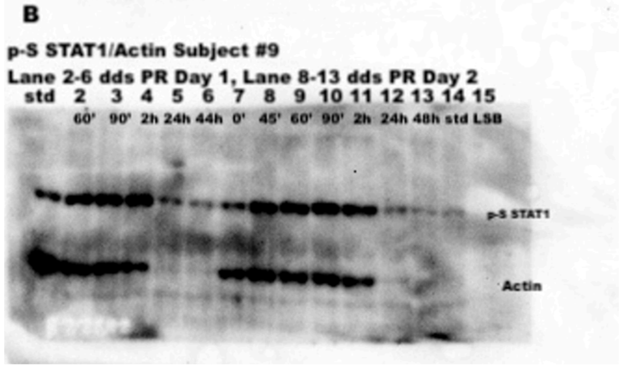
B
p-S STAT1/Actin Subject #9
Lane 2-6 dds PR Day 1, Lane 8-13 dds PR Day 2
std 2 3 4 5 6 7 8 9 10 11 12 13 14 15
60' 90' 2h 24h 44h 0' 45' 60' 90' 2h 24h 48h std LSB
p-S STAT1
Actin

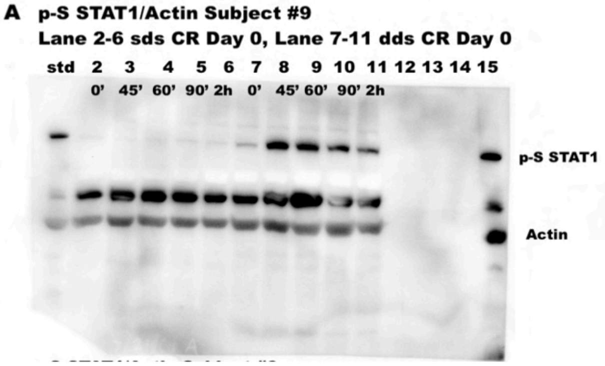
A p-S STAT1/Actin Subject #9
Lane 2-6 sds CR Day 0, Lane 7-11 dds CR Day 0
std 2 3 4 5 6 7 8 9 10 11 12 13 14 15
0' 45' 60' 90' 2h 0' 45' 60' 90' 2h
p-S STAT1
Actin

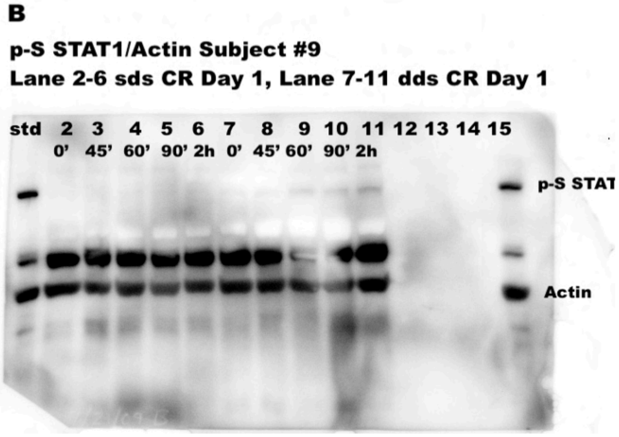
B
p-S STAT1/Actin Subject #9
Lane 2-6 sds CR Day 1, Lane 7-11 dds CR Day 1
std 2 3 4 5 6 7 8 9 10 11 12 13 14 15
0' 45' 60' 90' 2h 0' 45' 60' 90' 2h
Actin

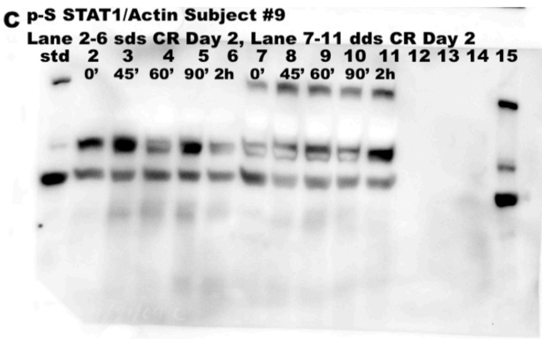
C p-S STAT1/Actin Subject #9
Lane 2-6 sds CR Day 2, Lane 7-11 dds CR Day 2
std 2 3 4 5 6 7 8 9 10 11 12 13 14 15
0' 45' 60' 90' 2h 0' 45' 60' 90' 2h
p-S STAT1
Actin

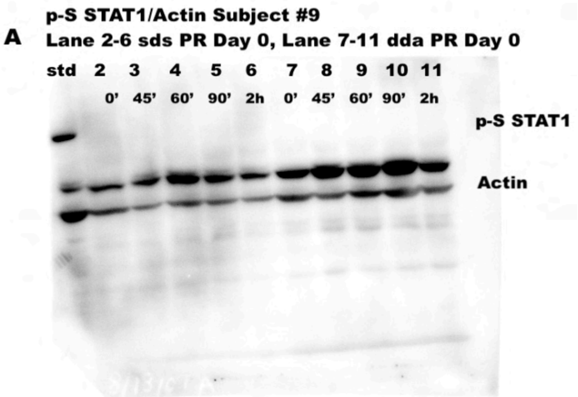
p-S STAT1/Actin Subject #9
A Lane 2-6 sds PR Day 0, Lane 7-11 dda PR Day 0
std 2 3 4 5 6 7 8 9 10 11
0' 45' 60' 90' 2h 0' 45' 60' 90' 2h
p-S STAT1
Actin

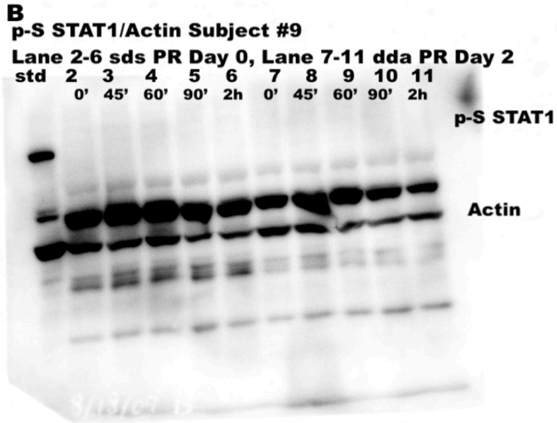
B
p-S STAT1/Actin Subject #9
Lane 2-6 sds PR Day 0, Lane 7-11 dda PR Day 2
std 2 3 4 5 6 7 8 9 10 11
0' 45' 60' 90' 2h 0' 45' 60' 90' 2h
p-S STAT1
Actin

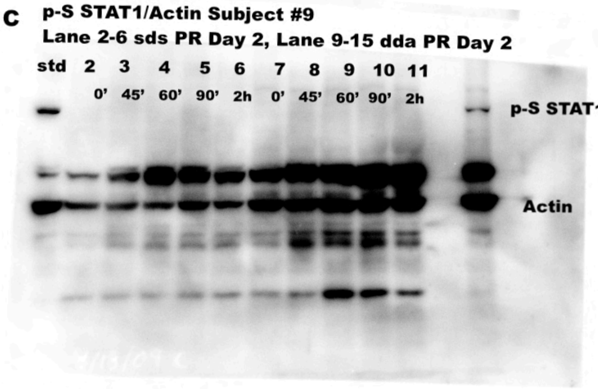
C p-S STAT1/Actin Subject #9
Lane 2-6 sds PR Day 2, Lane 9-15 dda PR Day 2
std 2 3 4 5 6 7 8 9 10 11
0' 45' 60' 90' 2h 0' 45' 60' 90' 2h
p-S STAT1
Actin

Subject #10

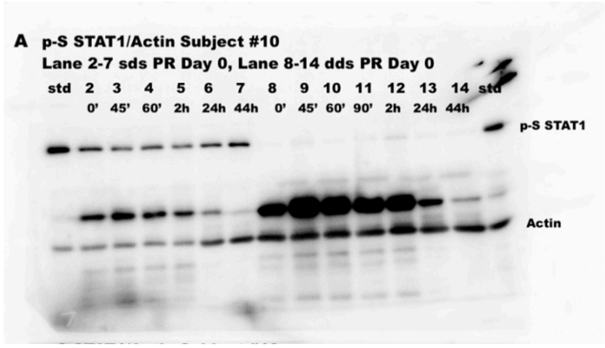
A p-S STAT1/Actin Subject #10
Lane 2-7 sds PR Day 0, Lane 8-14 dds PR Day 0
std 2 3 4 5 6 7 8 9 10 11 12 13 14 std
0' 45' 60' 2h 24h 44h 0' 45' 60' 90' 2h 24h 44h
p-S STAT1
Actin

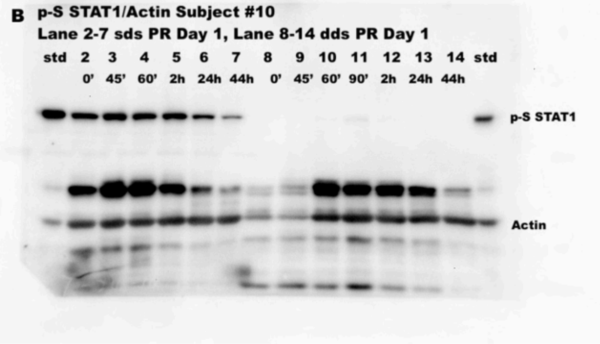
B p-S STAT1/Actin Subject #10
Lane 2-7 sds PR Day 1, Lane 8-14 dds PR Day 1
std 2 3 4 5 6 7 8 9 10 11 12 13 14 std
0' 45' 60' 2h 24h 44h 0' 45' 60' 90' 2h 24h 44h
p-S STAT1
Actin

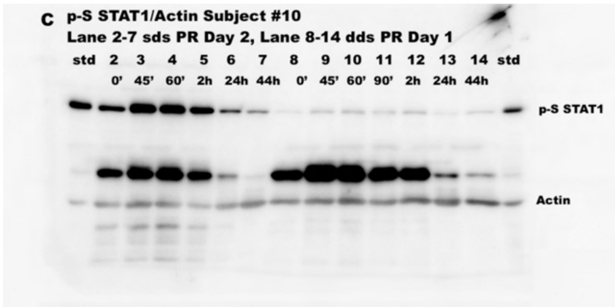
C p-S STAT1/Actin Subject #10
Lane 2-7 sds PR Day 2, Lane 8-14 dds PR Day 1
std 2 3 4 5 6 7 8 9 10 11 12 13 14 std
0' 45' 60' 2h 24h 44h 0' 45' 60' 90' 2h 24h 44h
p-S STAT1
Actin

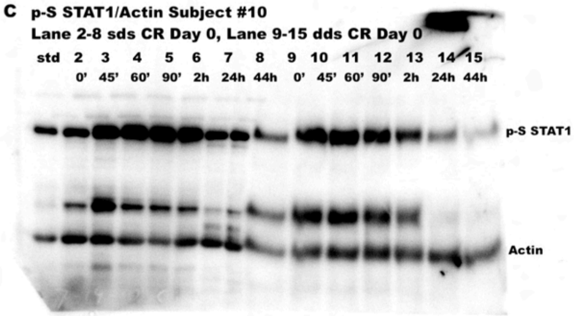
C p-S STAT1/Actin Subject #10
Lane 2-8 sds CR Day 0, Lane 9-15 dds CR Day 0
std 2 3 4 5 6 7 8 9 10 11 12 13 14 15
0' 45' 60' 90' 2h 24h 44h 0' 45' 60' 90' 2h 24h 44h
p-S STAT1
Actin

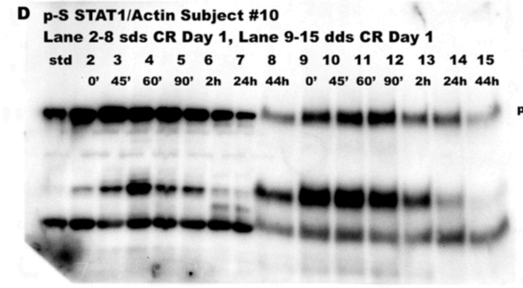
D
p-S STAT1/Actin Subject #10
Lane 2-8 sds CR Day 1, Lane 9-15 dds CR Day 1
std 2 3 4 5 6 7 8 9 10 11 12 13 14 15
0' 45' 60' 90' 2h 24h 44h 0' 45' 60' 90' 2h 24h 44h
p-S STAT1
Actin

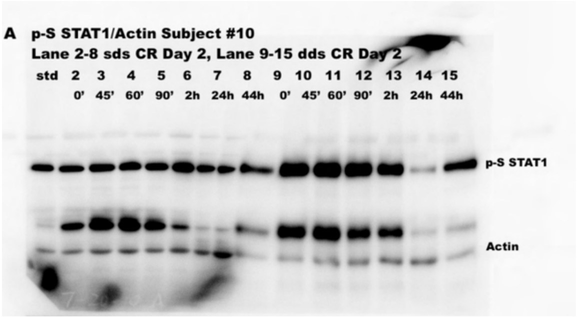
A
p-S STAT1/Actin Subject #10
Lane 2-8 sds CR Day 2, Lane 9-15 dds CR Day 2
std 2 3 4 5 6 7 8 9 10 11 12 13 14 15
0' 45' 60' 90' 2h 24h 44h 0' 45' 60' 90' 2h 24h 44h
p-S STAT1
Actin

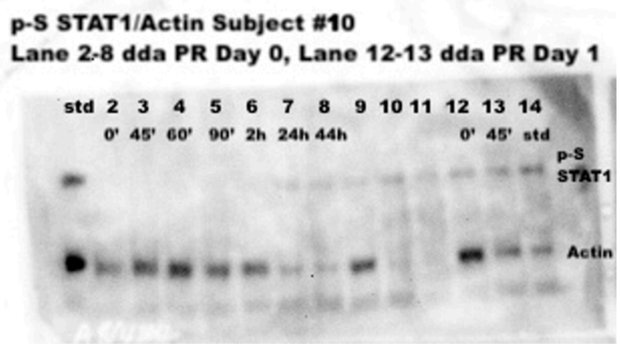
p-S STAT1/Actin Subject #10
Lane 2-8 dda PR Day 0, Lane 12-13 dda PR Day 1
std 2 3 4 5 6 7 8 9 10 11 12 13 14
0' 45' 60' 90' 2h 24h 44h 0' 45' std
p-S STAT1
Actin

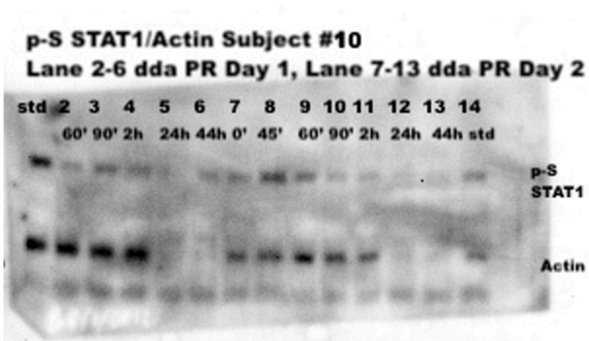
p-S STAT1/Actin Subject #10
Lane 2-6 dda PR Day 1, Lane 7-13 dda PR Day 2
std 2 3 4 5 6 7 8 9 10 11 12 13 14
60' 90' 2h 24h 44h 0' 45' 60' 90' 2h 24h 44h std
p-S STAT1
Actin

Subject #11

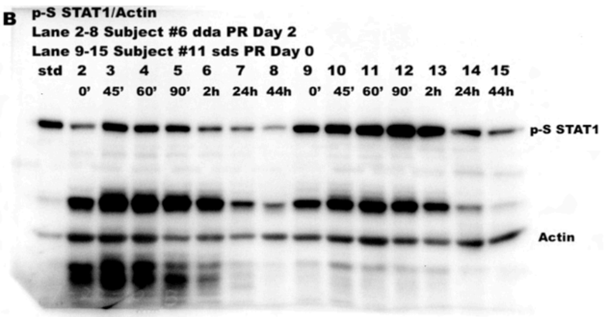
B
p-S STAT1/Actin
Lane 2-8 Subject #6 dda PR Day 2
Lane 9-15 Subject #11 sds PR Day 0
std 2 3 4 5 6 7 8 9 10 11 12 13 14 15
0' 45' 60' 90' 2h 24h 44h 0' 45' 60' 90' 2h 24h 44h
p-S STAT1
Actin

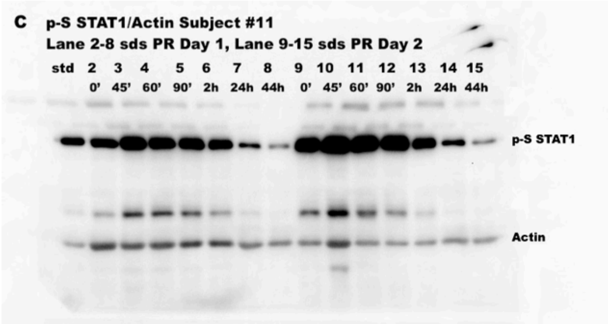
C
p-S STAT1/Actin Subject #11
Lane 2-8 sds PR Day 1, Lane 9-15 sds PR Day 2
std 2 3 4 5 6 7 8 9 10 11 12 13 14 15
0' 45' 60' 90' 2h 24h 44h 0' 45' 60' 90' 2h 24h 44h
p-S STAT1
Actin

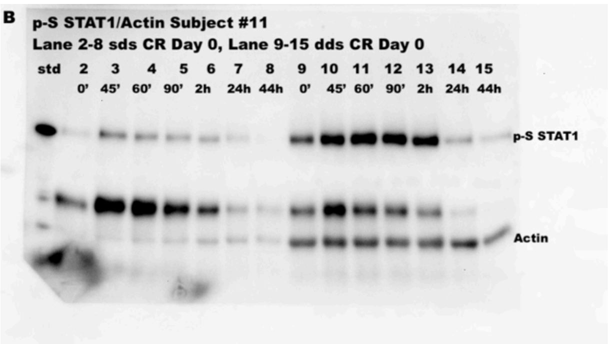
B
p-S STAT1/Actin Subject #11
Lane 2-8 sds CR Day 0, Lane 9-15 dds CR Day 0
std 2 3 4 5 6 7 8 9 10 11 12 13 14 15
0' 45' 60' 90' 2h 24h 44h 0' 45' 60' 90' 2h 24h 44h
p-S STAT1
Actin

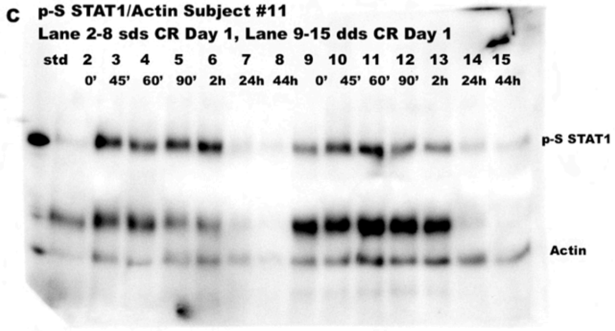
C
p-S STAT1/Actin Subject #11
Lane 2-8 sds CR Day 1, Lane 9-15 dds CR Day 1
std 2 3 4 5 6 7 8 9 10 11 12 13 14 15
0' 45' 60' 90' 2h 24h 44h 0' 45' 60' 90' 2h 24h 44h
p-S STAT1
Actin

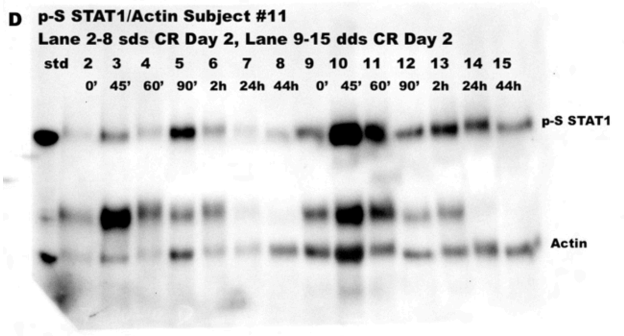
D
p-S STAT1/Actin Subject #11
Lane 2-8 sds CR Day 2, Lane 9-15 dds CR Day 2
std 2 3 4 5 6 7 8 9 10 11 12 13 14 15
0' 45' 60' 90' 2h 24h 44h 0' 45' 60' 90' 2h 24h 44h
p-S STAT1
Actin

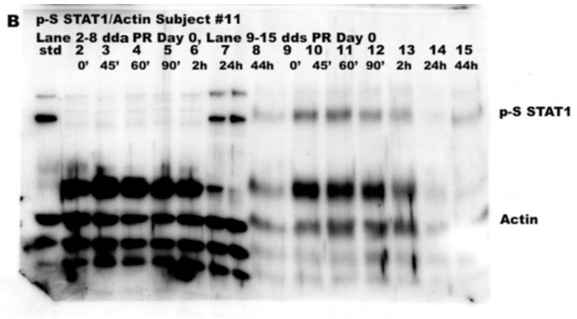
B
p-S STAT1/Actin Subject #11
Lane 2-8 dda PR Day 0, Lane 9-15 dds PR Day 0
std 2 3 4 5 6 7 8 9 10 11 12 13 14 15
0' 45' 60' 90' 2h 24h 44h 0' 45' 60' 90' 2h 24h 44h
p-S STAT1
Actin

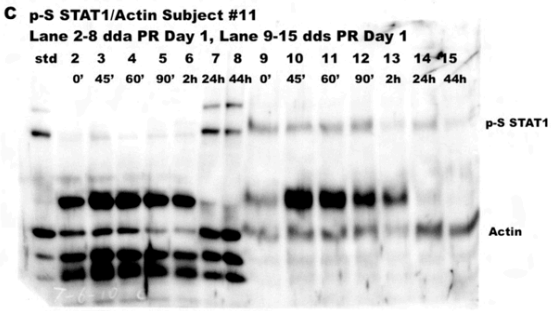
C
p-S STAT1/Actin Subject #11
Lane 2-8 dda PR Day 1, Lane 9-15 dds PR Day 1
std 2 3 4 5 6 7 8 9 10 11 12 13 14 15
0' 45' 60' 90' 2h 24h 44h 0' 45' 60' 90' 2h 24h 44h
p-S STAT1
Actin

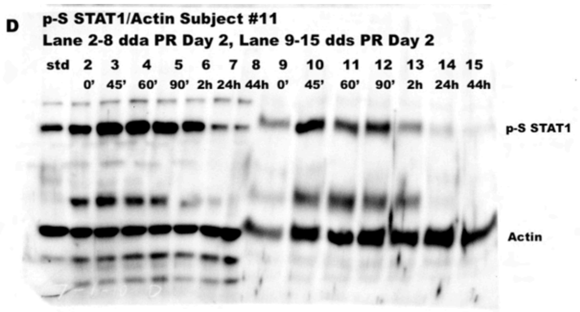
D
p-S STAT1/Actin Subject #11
Lane 2-8 dda PR Day 2, Lane 9-15 dds PR Day 2
std 2 3 4 5 6 7 8 9 10 11 12 13 14 15
0' 45' 60' 90' 2h 24h 44h 0' 45' 60' 90' 2h 24h 44h
p-S STAT1
Actin

Subject #12

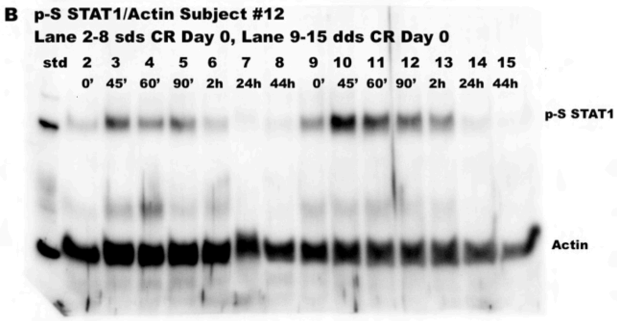
B
p-S STAT1/Actin Subject #12
Lane 2-8 sds CR Day 0, Lane 9-15 dds CR Day 0
std 2 3 4 5 6 7 8 9 10 11 12 13 14 15
0' 45' 60' 90' 2h 24h 44h 0' 45' 60' 90' 2h 24h 44h
p-S STAT1
Actin

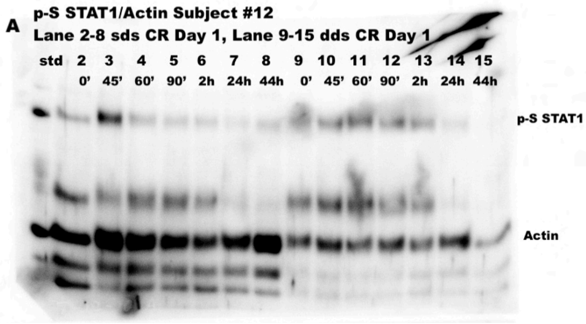
A
p-S STAT1/Actin Subject #12
Lane 2-8 sds CR Day 1, Lane 9-15 dds CR Day 1
std 2 3 4 5 6 7 8 9 10 11 12 13 14 15
0' 45' 60' 90' 2h 24h 44h 0' 45' 60' 90' 2h 24h 44h
p-S STAT1
Actin

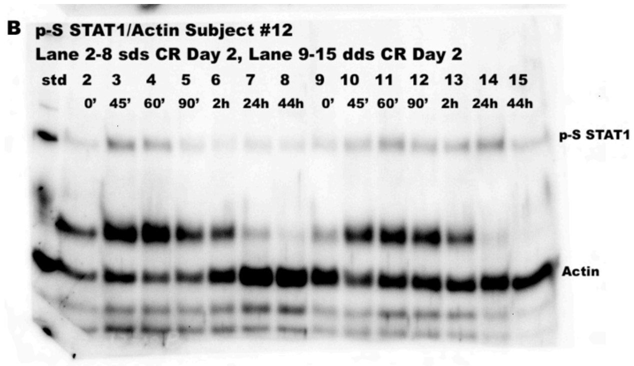
B
p-S STAT1/Actin Subject #12
Lane 2-8 sds CR Day 2, Lane 9-15 dds CR Day 2
std 2 3 4 5 6 7 8 9 10 11 12 13 14 15
0' 45' 60' 90' 2h 24h 44h 0' 45' 60' 90' 2h 24h 44h
p-S STAT1
Actin

Subject #13

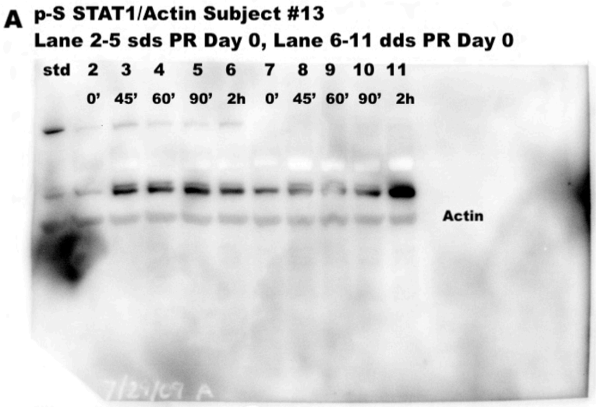

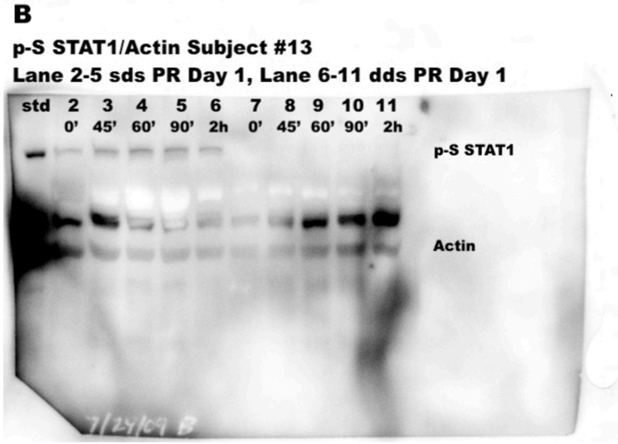

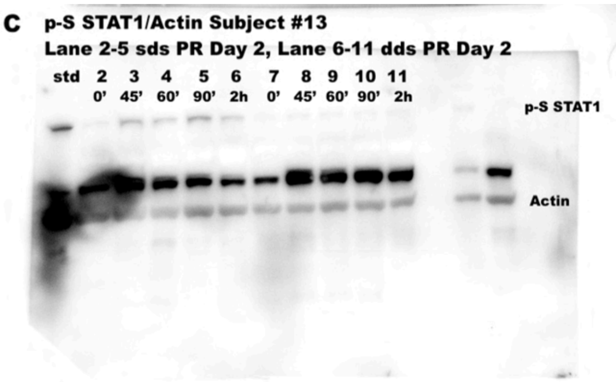

Subject #14

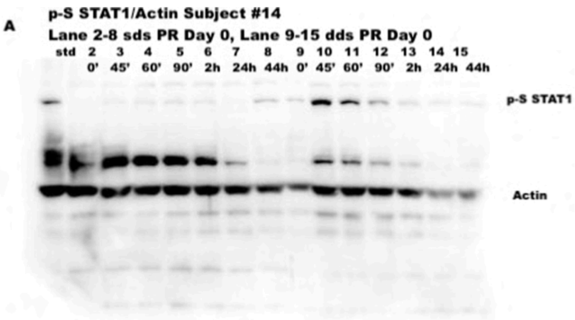

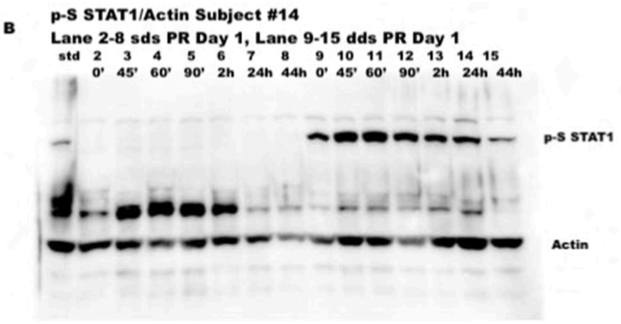

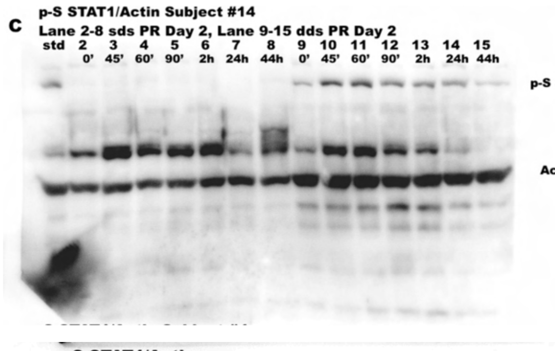

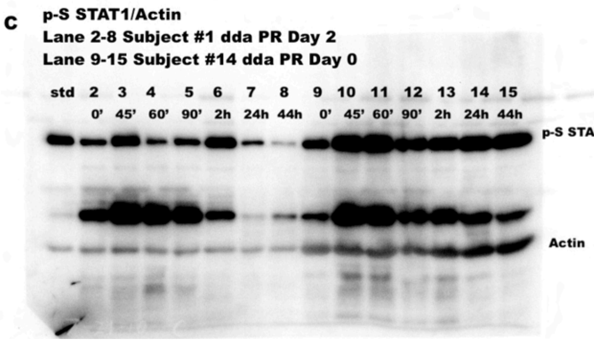

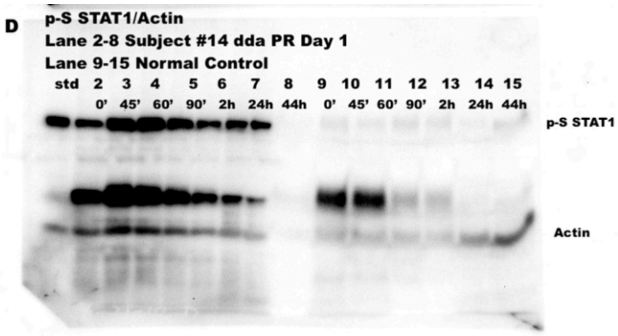

MxA

Subject #1

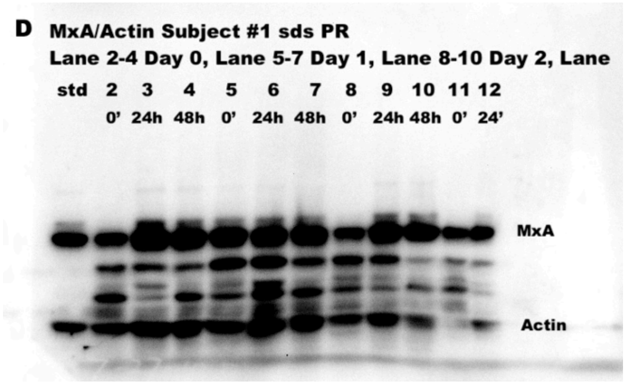

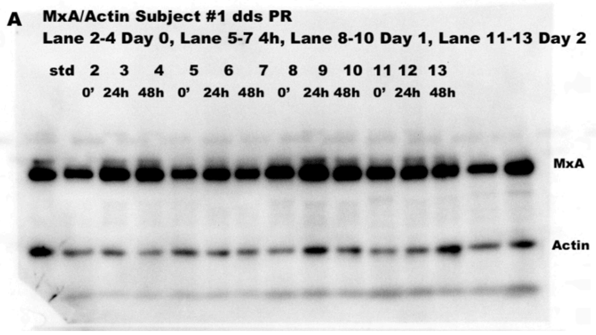

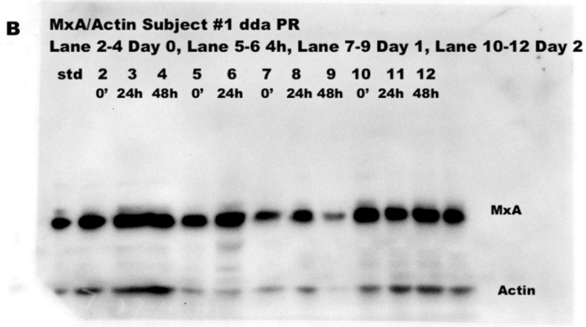

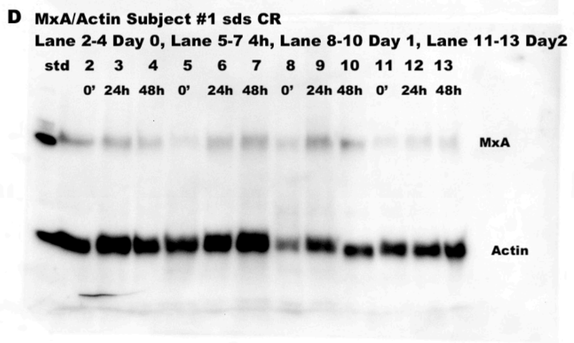

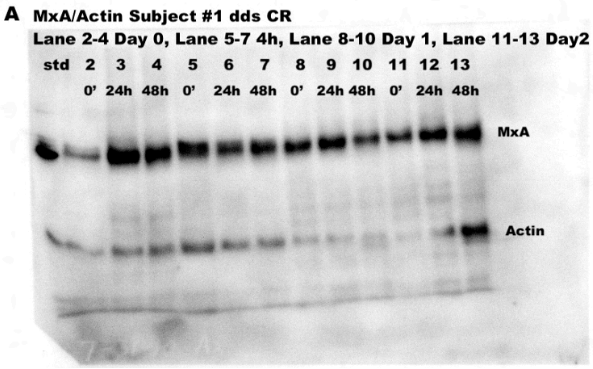
A MxA/Actin Subject #1 dds CR
Lane 2-4 Day 0, Lane 5-7 4h, Lane 8-10 Day 1, Lane 11-13 Day2
std 2 3 4 5 6 7 8 9 10 11 12 13
0' 24h 48h 0' 24h 48h 0' 24h 48h 0' 24h 48h
MxA
Actin

Subject #2

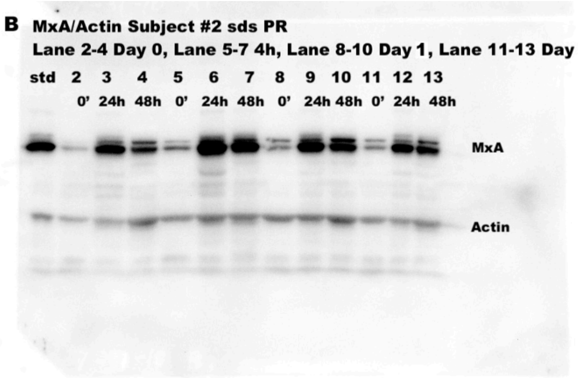
B MxA/Actin Subject #2 sds PR
Lane 2-4 Day 0, Lane 5-7 4h, Lane 8-10 Day 1, Lane 11-13 Day 2
std 2 3 4 5 6 7 8 9 10 11 12 13
0' 24h 48h 0' 24h 48h 0' 24h 48h 0' 24h 48h
MxA
Actin

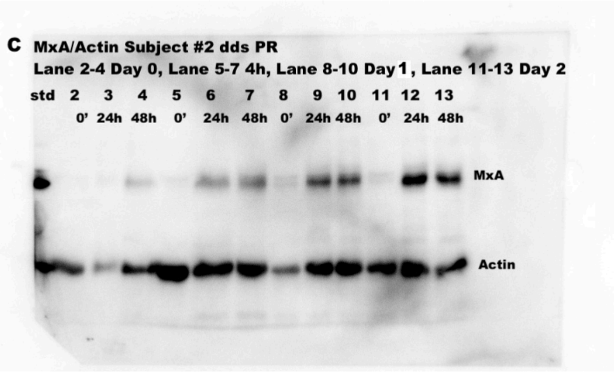
C MxA/Actin Subject #2 dds PR
Lane 2-4 Day 0, Lane 5-7 4h, Lane 8-10 Day 1, Lane 11-13 Day 2
std 2 3 4 5 6 7 8 9 10 11 12 13
0' 24h 48h 0' 24h 48h 0' 24h 48h 0' 24h 48h
MxA
Actin

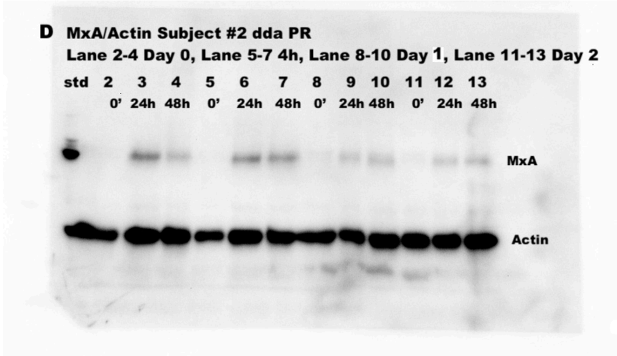
D MxA/Actin Subject #2 dda PR
Lane 2-4 Day 0, Lane 5-7 4h, Lane 8-10 Day 1, Lane 11-13 Day 2
std 2 3 4 5 6 7 8 9 10 11 12 13
0' 24h 48h 0' 24h 48h 0' 24h 48h 0' 24h 48h
MxA
Actin

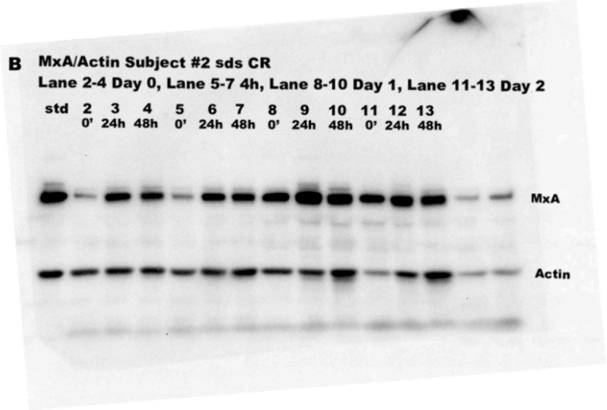
B MxA/Actin Subject #2 sds CR
Lane 2-4 Day 0, Lane 5-7 4h, Lane 8-10 Day 1, Lane 11-13 Day 2
std 2 3 4 5 6 7 8 9 10 11 12 13
0' 24h 48h 0' 24h 48h 0' 24h 48h 0' 24h 48h
MxA
Actin

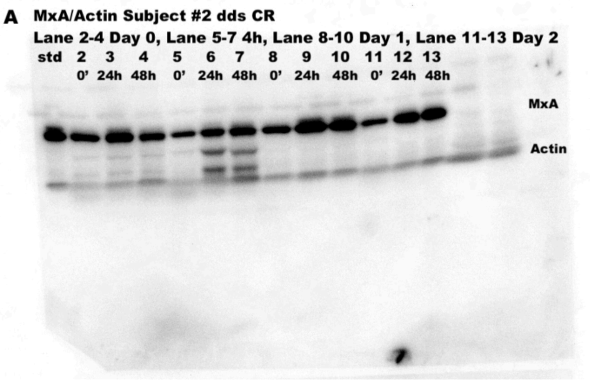
A MxA/Actin Subject #2 dds CR
Lane 2-4 Day 0, Lane 5-7 4h, Lane 8-10 Day 1, Lane 11-13 Day 2
std 2 3 4 5 6 7 8 9 10 11 12 13
0' 24h 48h 0' 24h 48h 0' 24h 48h 0' 24h 48h
MxA
Actin

Subject #3

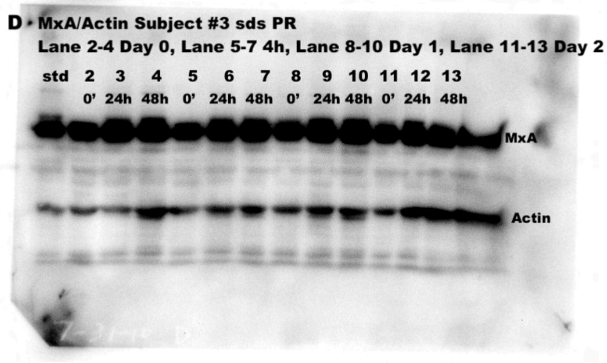

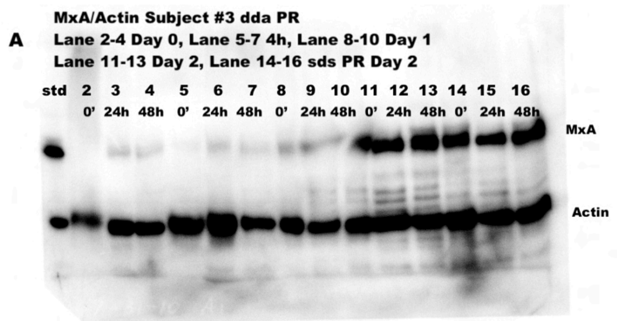

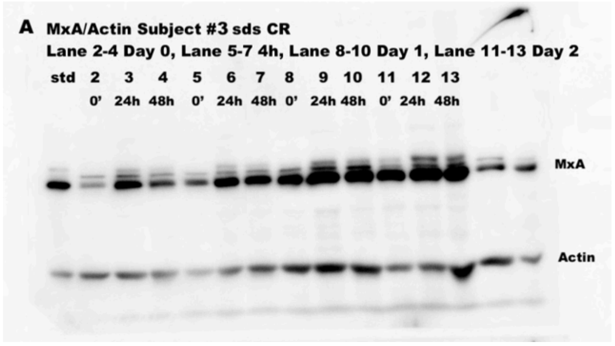

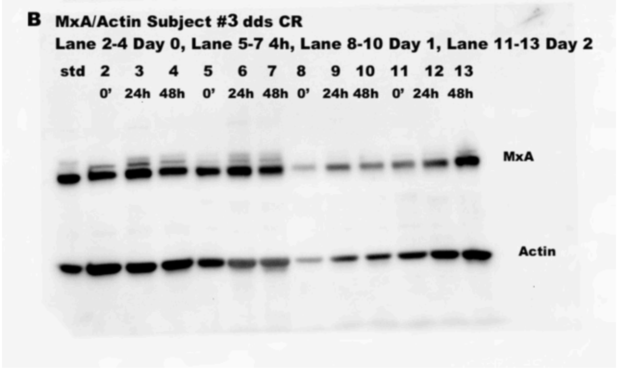

Subject #4

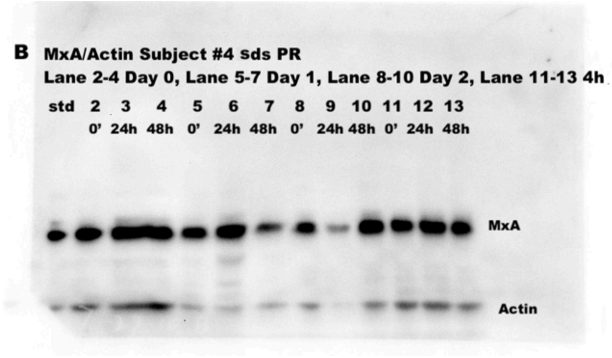

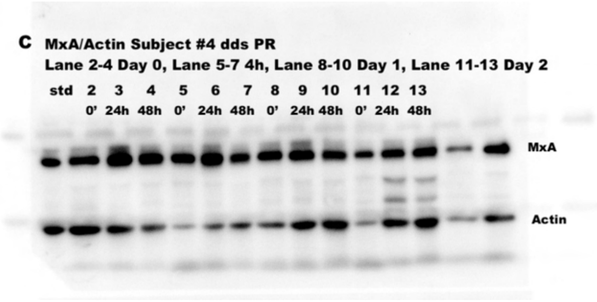

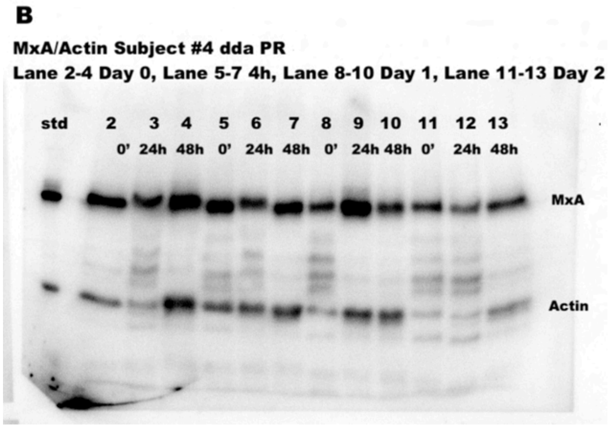

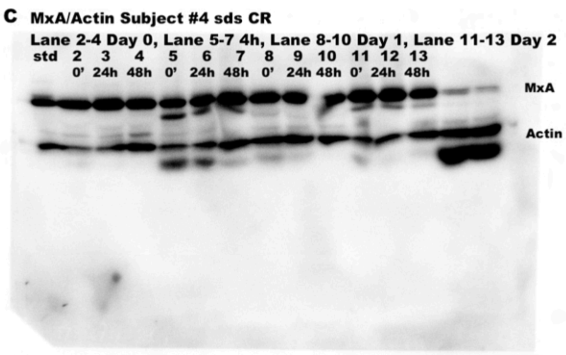

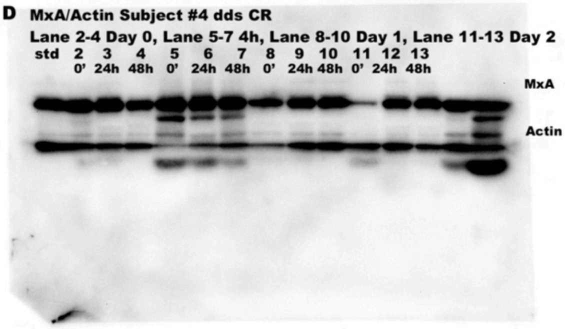

Subject #5

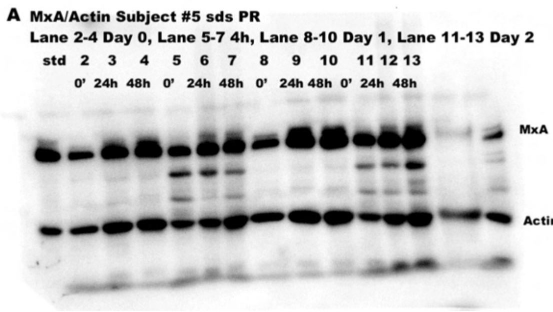

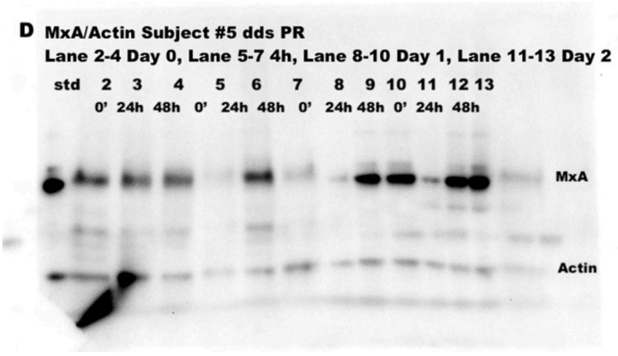

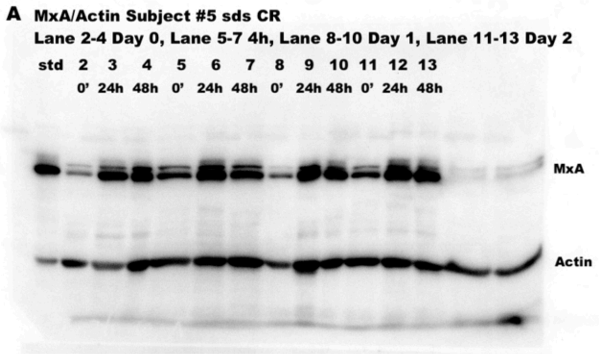

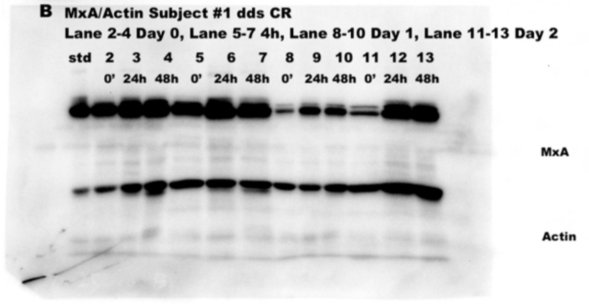

Subject #6

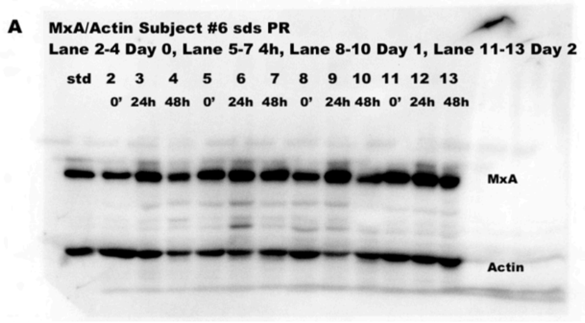

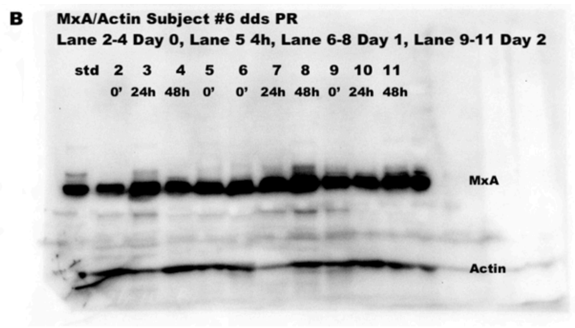

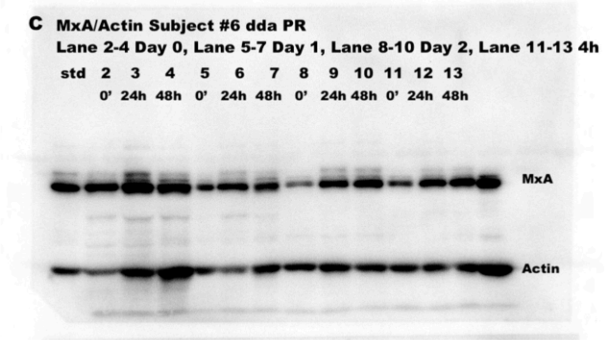
C MxA/Actin Subject #6 dda PR
Lane 2-4 Day 0, Lane 5-7 Day 1, Lane 8-10 Day 2, Lane 11-13 4h
std 2 3 4 5 6 7 8 9 10 11 12 13
0' 24h 48h 0' 24h 48h 0' 24h 48h 0' 24h 48h
MxA
Actin

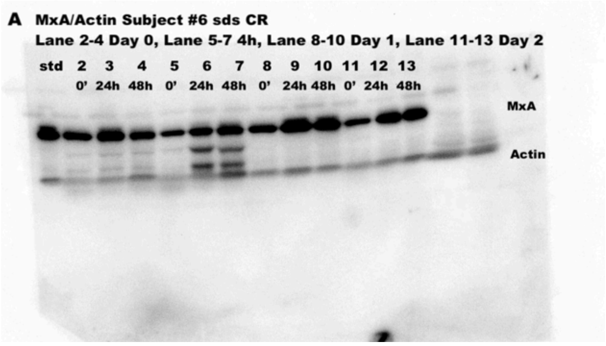
A MxA/Actin Subject #6 sds CR
Lane 2-4 Day 0, Lane 5-7 4h, Lane 8-10 Day 1, Lane 11-13 Day 2
std 2 3 4 5 6 7 8 9 10 11 12 13
0' 24h 48h 0' 24h 48h 0' 24h 48h 0' 24h 48h
MxA
Actin

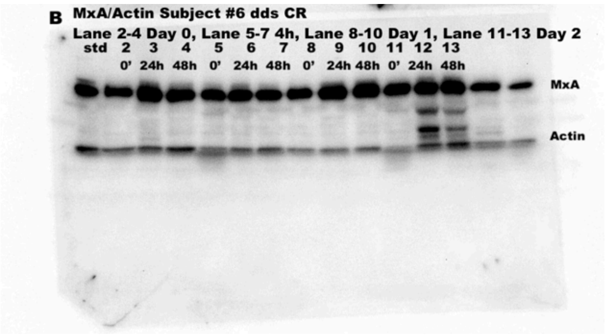
B MxA/Actin Subject #6 dds CR
Lane 2-4 Day 0, Lane 5-7 4h, Lane 8-10 Day 1, Lane 11-13 Day 2
std 2 3 4 5 6 7 8 9 10 11 12 13
0' 24h 48h 0' 24h 48h 0' 24h 48h 0' 24h 48h
MxA
Actin

Subject #7

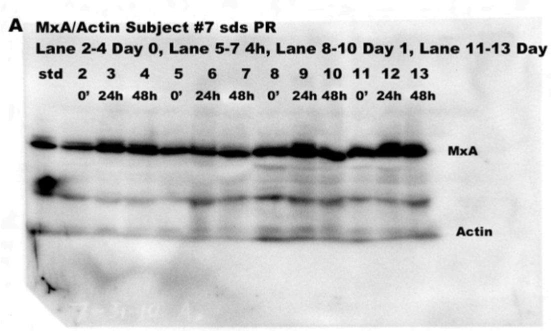
A MxA/Actin Subject #7 sds PR
Lane 2-4 Day 0, Lane 5-7 4h, Lane 8-10 Day 1, Lane 11-13 Day 2
std 2 3 4 5 6 7 8 9 10 11 12 13
0' 24h 48h 0' 24h 48h 0' 24h 48h 0' 24h 48h
MxA
Actin

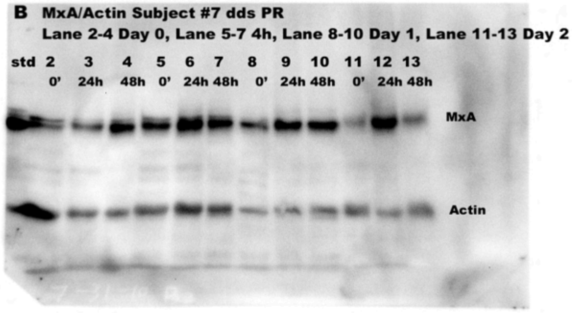
B MxA/Actin Subject #7 dds PR
Lane 2-4 Day 0, Lane 5-7 4h, Lane 8-10 Day 1, Lane 11-13 Day 2
std 2 3 4 5 6 7 8 9 10 11 12 13
0' 24h 48h 0' 24h 48h 0' 24h 48h 0' 24h 48h
MxA
Actin

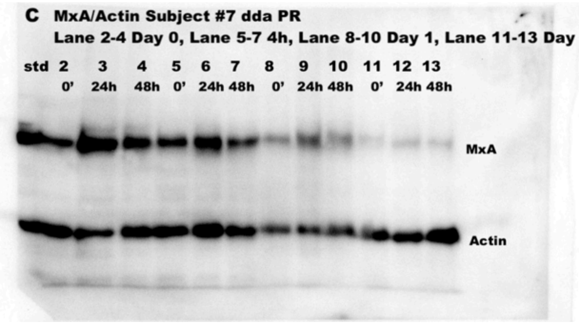
C MxA/Actin Subject #7 dda PR
Lane 2-4 Day 0, Lane 5-7 4h, Lane 8-10 Day 1, Lane 11-13 Day 2
std 2 3 4 5 6 7 8 9 10 11 12 13
0' 24h 48h 0' 24h 48h 0' 24h 48h 0' 24h 48h
MxA
Actin

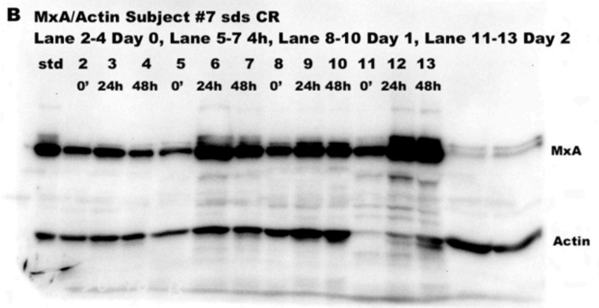
B MxA/Actin Subject #7 sds CR
Lane 2-4 Day 0, Lane 5-7 4h, Lane 8-10 Day 1, Lane 11-13 Day 2
std 2 3 4 5 6 7 8 9 10 11 12 13
0' 24h 48h 0' 24h 48h 0' 24h 48h 0' 24h 48h
MxA
Actin

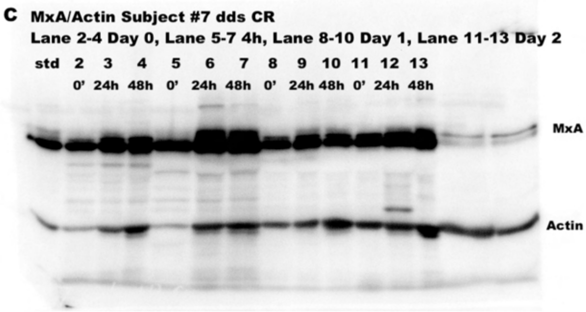
C MxA/Actin Subject #7 dds CR
Lane 2-4 Day 0, Lane 5-7 4h, Lane 8-10 Day 1, Lane 11-13 Day 2
std 2 3 4 5 6 7 8 9 10 11 12 13
0' 24h 48h 0' 24h 48h 0' 24h 48h 0' 24h 48h
MxA
Actin

Subject #8

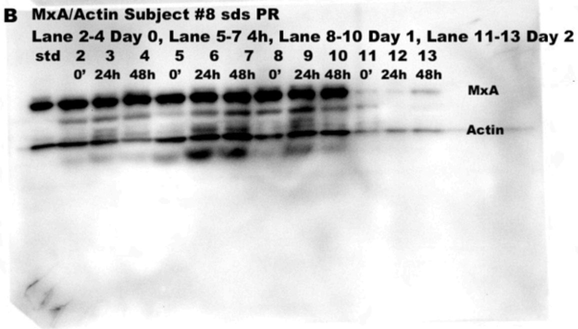
B MxA/Actin Subject #8 sds PR
Lane 2-4 Day 0, Lane 5-7 4h, Lane 8-10 Day 1, Lane 11-13 Day 2
std 2 3 4 5 6 7 8 9 10 11 12 13
0' 24h 48h 0' 24h 48h 0' 24h 48h 0' 24h 48h
MxA
Actin

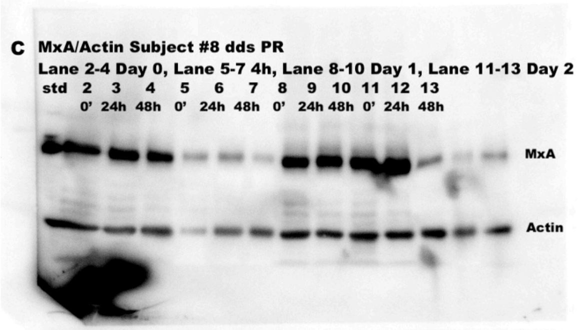
C MxA/Actin Subject #8 dds PR
Lane 2-4 Day 0, Lane 5-7 4h, Lane 8-10 Day 1, Lane 11-13 Day 2
std 2 3 4 5 6 7 8 9 10 11 12 13
0' 24h 48h 0' 24h 48h 0' 24h 48h 0' 24h 48h
MxA
Actin

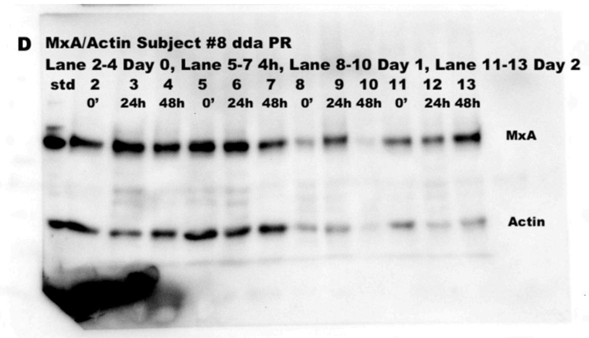
D MxA/Actin Subject #8 dda PR
Lane 2-4 Day 0, Lane 5-7 4h, Lane 8-10 Day 1, Lane 11-13 Day 2
std 2 3 4 5 6 7 8 9 10 11 12 13
0' 24h 48h 0' 24h 48h 0' 24h 48h 0' 24h 48h
MxA
Actin

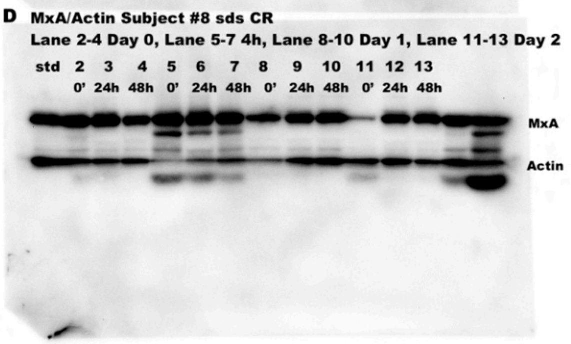
D MxA/Actin Subject #8 sds CR
Lane 2-4 Day 0, Lane 5-7 4h, Lane 8-10 Day 1, Lane 11-13 Day 2
std 2 3 4 5 6 7 8 9 10 11 12 13
0' 24h 48h 0' 24h 48h 0' 24h 48h 0' 24h 48h
MxA
Actin

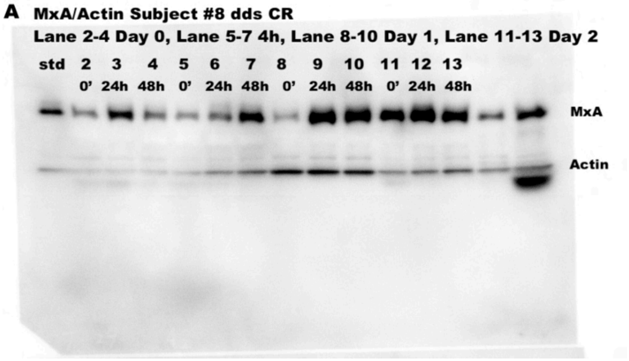
A MxA/Actin Subject #8 dds CR
Lane 2-4 Day 0, Lane 5-7 4h, Lane 8-10 Day 1, Lane 11-13 Day 2
std 2 3 4 5 6 7 8 9 10 11 12 13
0' 24h 48h 0' 24h 48h 0' 24h 48h 0' 24h 48h
MxA
Actin

Subject #9

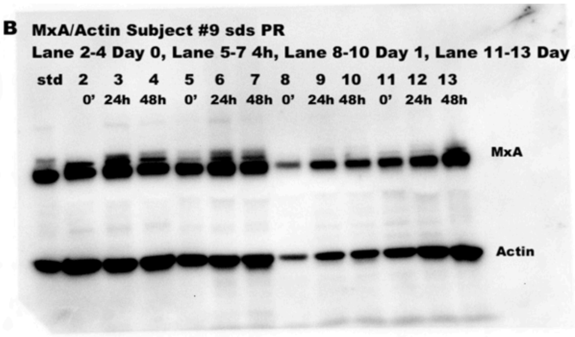

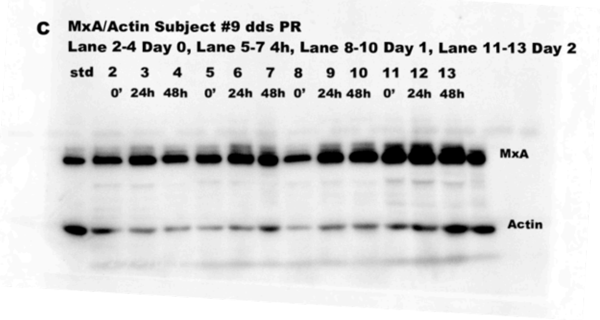

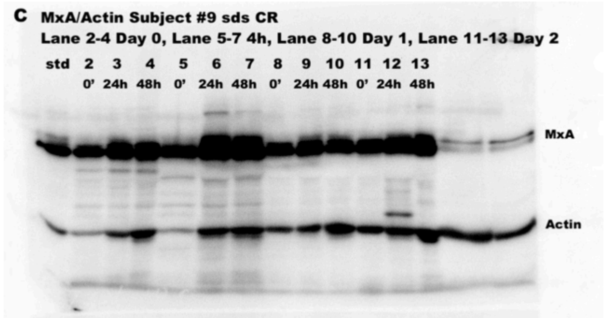

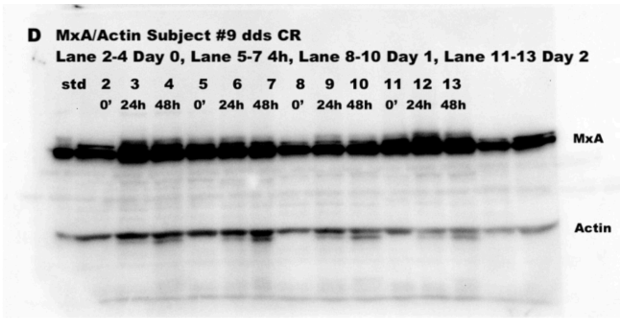

Subject #10

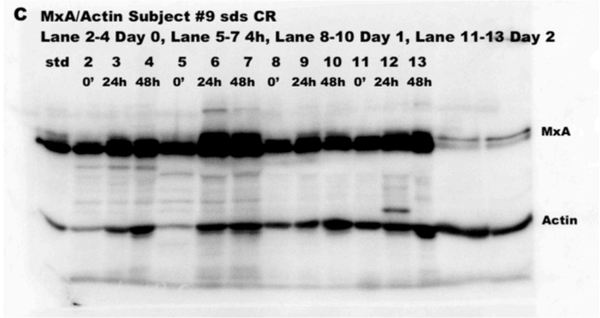

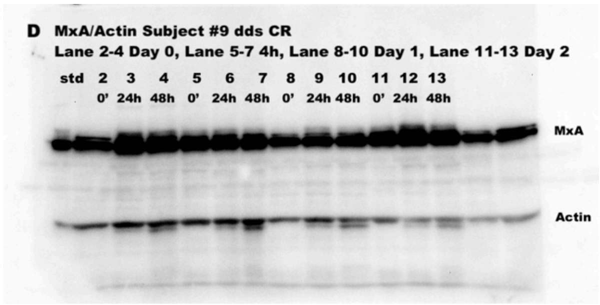

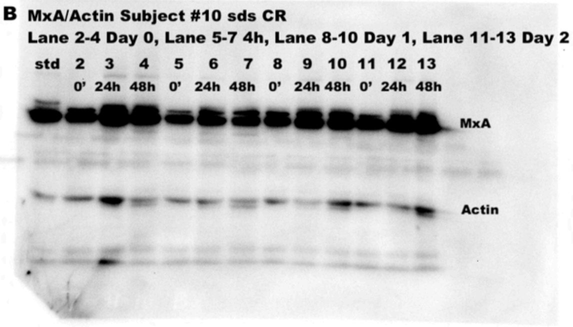

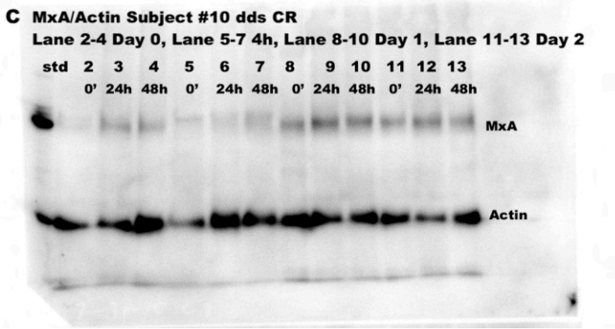

Subject #11

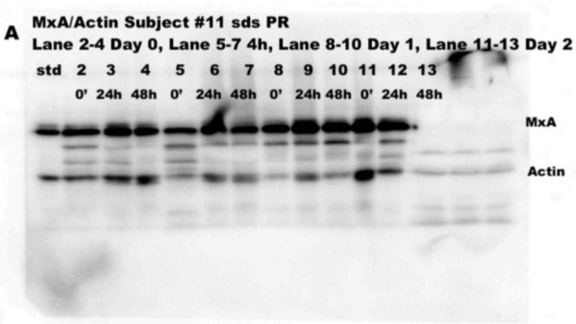

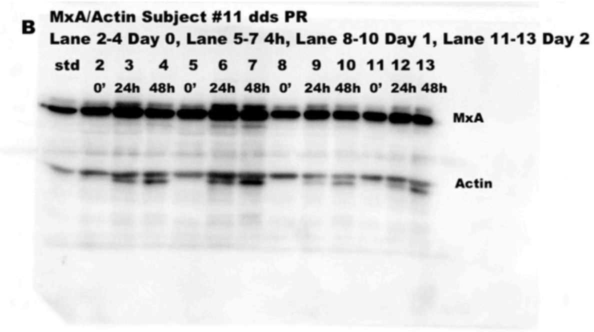

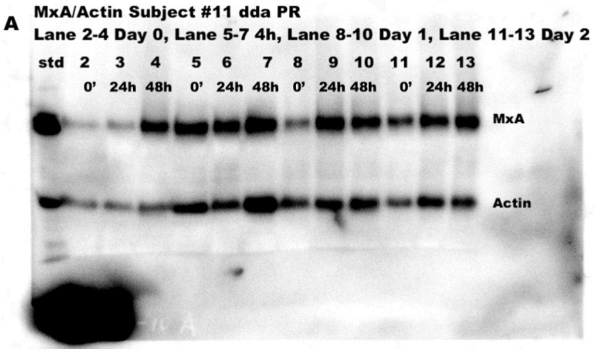

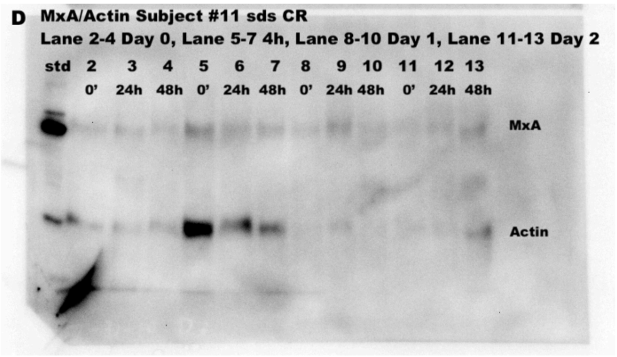

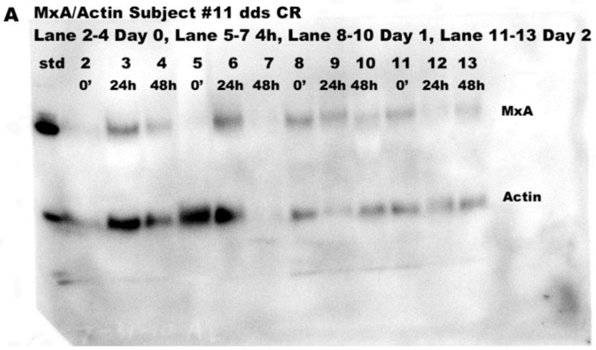

Subject #12

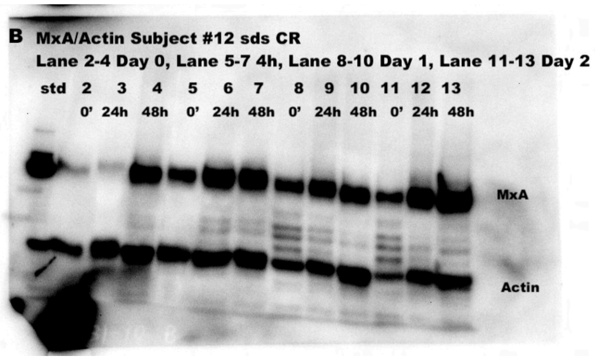

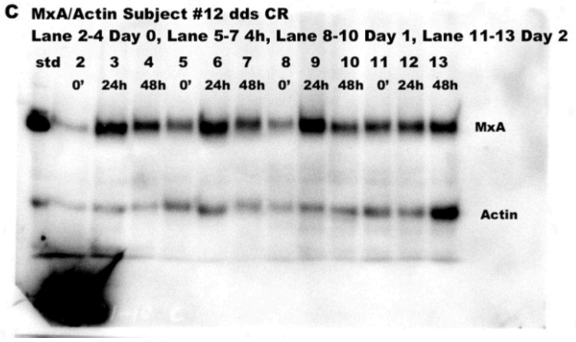

Subject #13

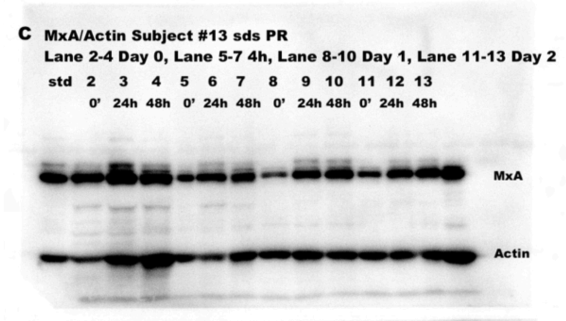

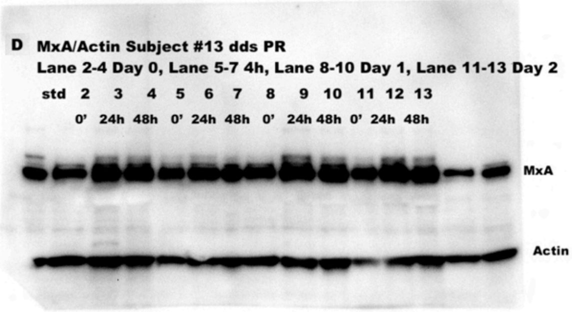

Subject #14

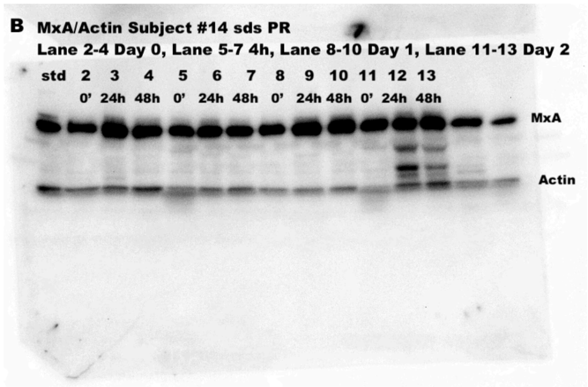

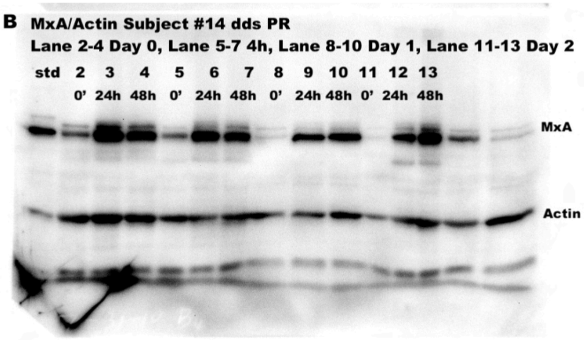

U-STAT1

Subject #1

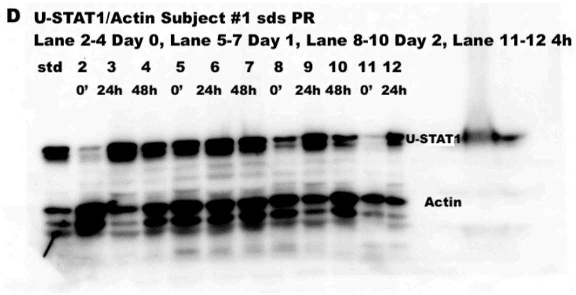

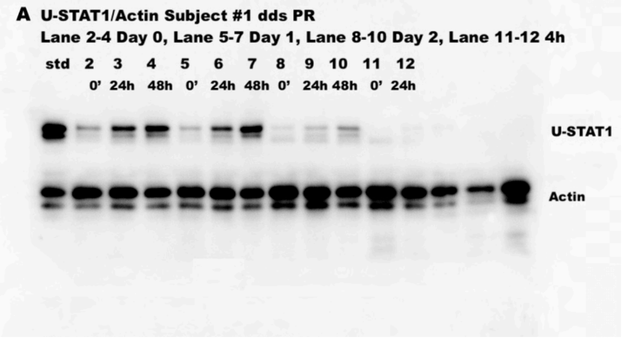

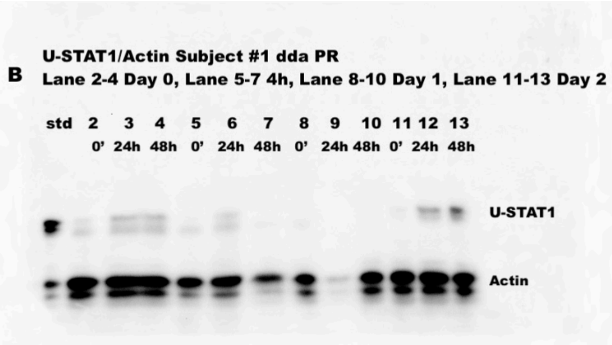
B
U-STAT1/Actin Subject #1 dda PR
Lane 2-4 Day 0, Lane 5-7 4h, Lane 8-10 Day 1, Lane 11-13 Day 2
std 2 3 4 5 6 7 8 9 10 11 12 13
0' 24h 48h 0' 24h 48h 0' 24h 48h 0' 24h 48h
U-STAT1
Actin

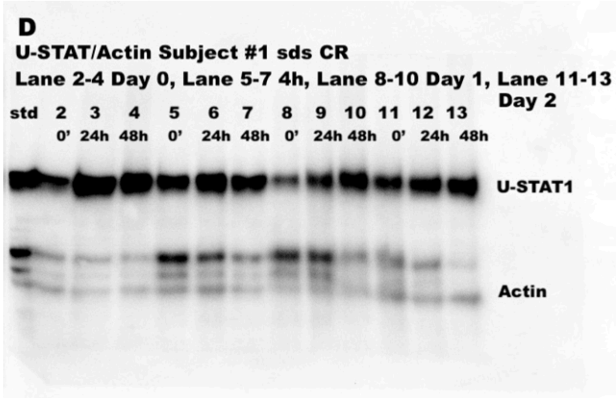
D
U-STAT/Actin Subject #1 sds CR
Lane 2-4 Day 0, Lane 5-7 4h, Lane 8-10 Day 1, Lane 11-13 Day 2
std 2 3 4 5 6 7 8 9 10 11 12 13
0' 24h 48h 0' 24h 48h 0' 24h 48h 0' 24h 48h
U-STAT1
Actin

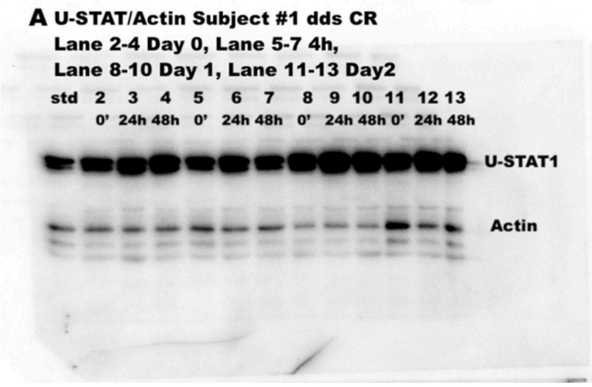
A U-STAT/Actin Subject #1 dds CR
Lane 2-4 Day 0, Lane 5-7 4h,
Lane 8-10 Day 1, Lane 11-13 Day2
std 2 3 4 5 6 7 8 9 10 11 12 13
0' 24h 48h 0' 24h 48h 0' 24h 48h 0' 24h 48h
U-STAT1
Actin

Subject #2

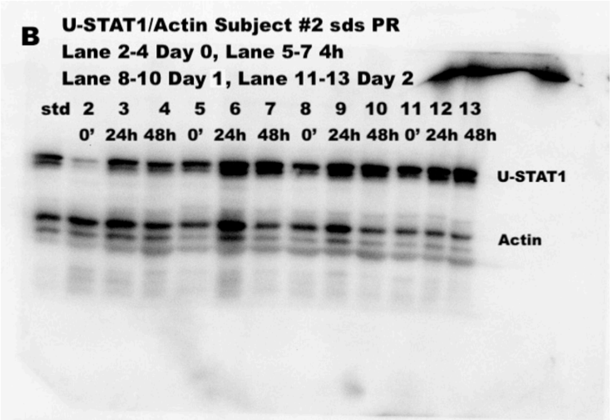
B
U-STAT1/Actin Subject #2 sds PR
Lane 2-4 Day 0, Lane 5-7 4h
Lane 8-10 Day 1, Lane 11-13 Day 2
std 2 3 4 5 6 7 8 9 10 11 12 13
0' 24h 48h 0' 24h 48h 0' 24h 48h 0' 24h 48h
U-STAT1
Actin

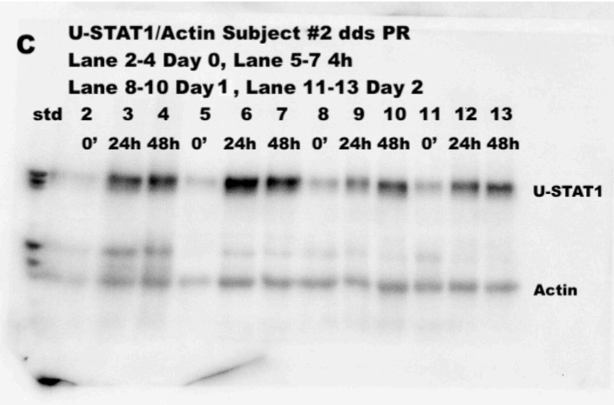
C
U-STAT1/Actin Subject #2 dds PR
Lane 2-4 Day 0, Lane 5-7 4h
Lane 8-10 Day 1, Lane 11-13 Day 2
std 2 3 4 5 6 7 8 9 10 11 12 13
0' 24h 48h 0' 24h 48h 0' 24h 48h 0' 24h 48h
U-STAT1
Actin

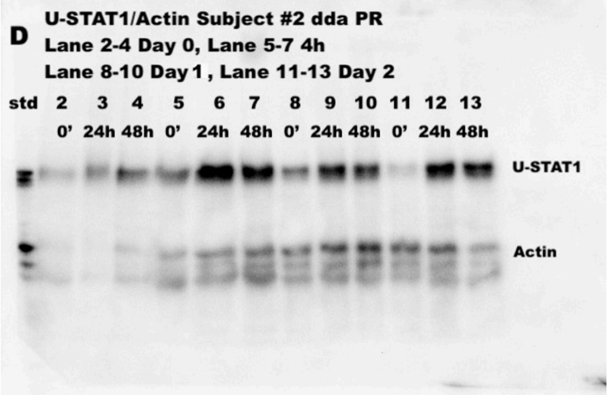
D
U-STAT1/Actin Subject #2 dda PR
Lane 2-4 Day 0, Lane 5-7 4h
Lane 8-10 Day 1, Lane 11-13 Day 2
std 2 3 4 5 6 7 8 9 10 11 12 13
0' 24h 48h 0' 24h 48h 0' 24h 48h 0' 24h 48h
U-STAT1
Actin

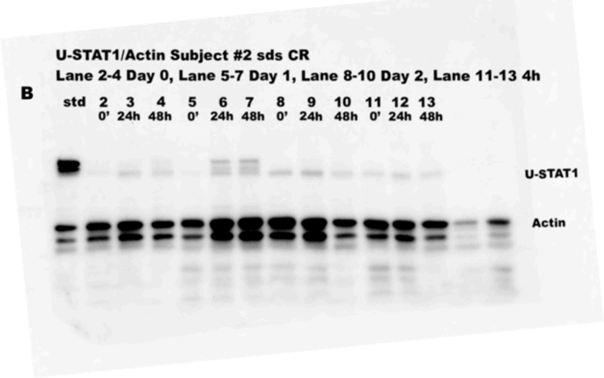
B
U-STAT1/Actin Subject #2 sds CR
Lane 2-4 Day 0, Lane 5-7 Day 1, Lane 8-10 Day 2, Lane 11-13 4h
std 2 3 4 5 6 7 8 9 10 11 12 13
0' 24h 48h 0' 24h 48h 0' 24h 48h 0' 24h 48h
U-STAT1
Actin

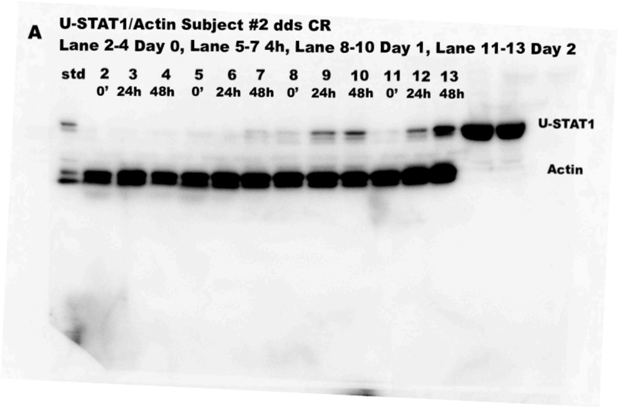

Subject #3

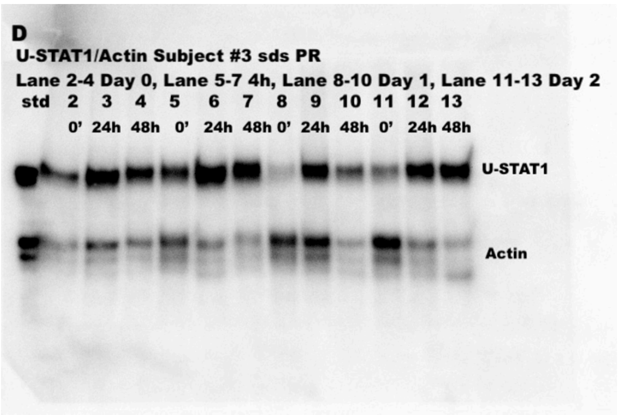

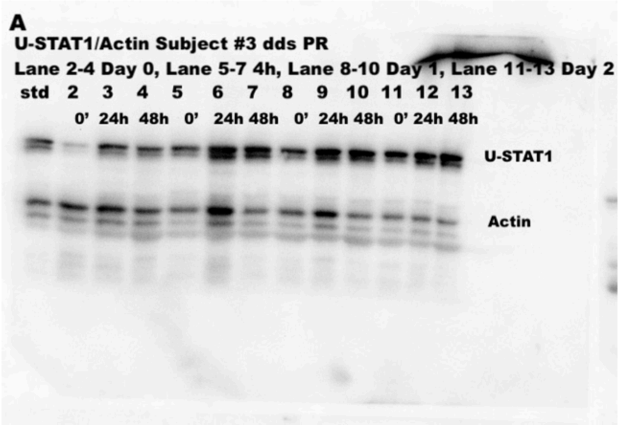

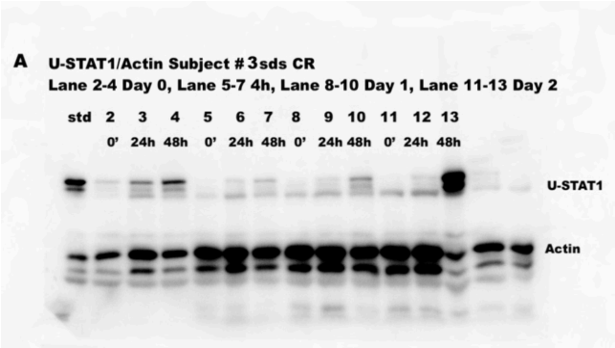

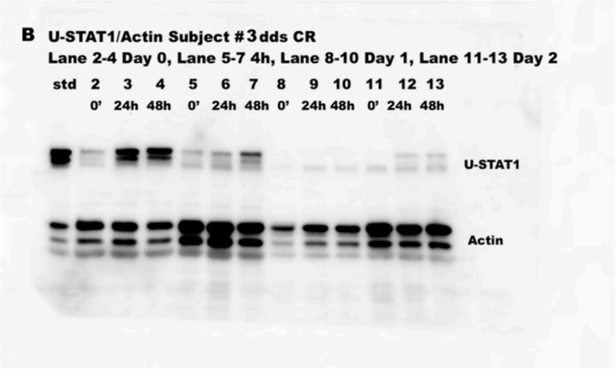

Subject #4

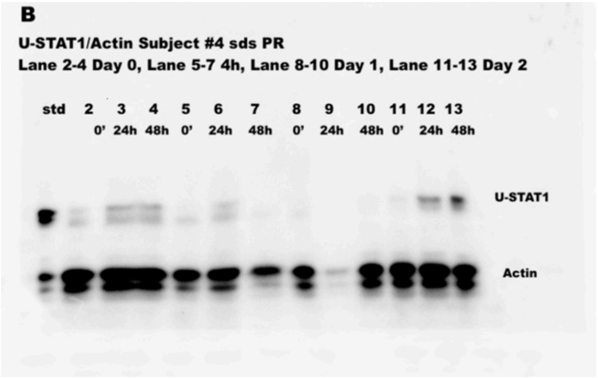

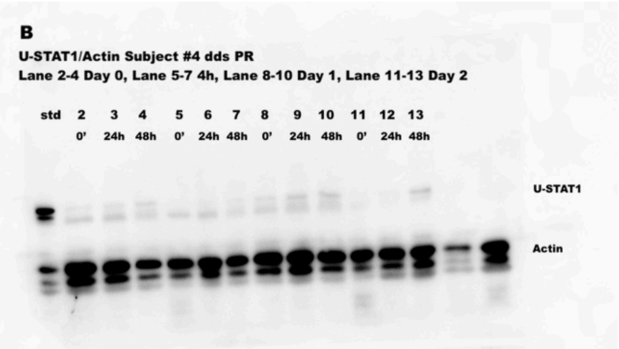

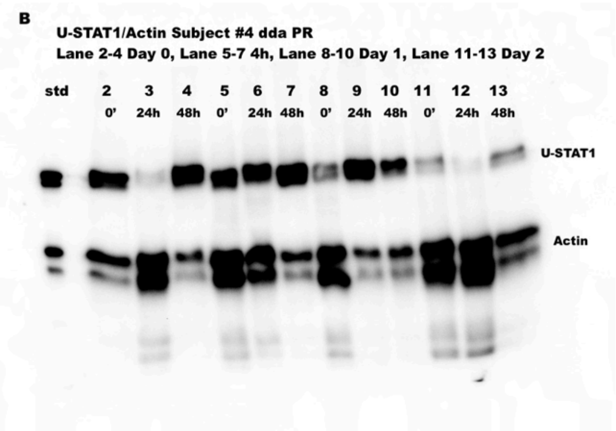
B
U-STAT1/Actin Subject #4 dda PR
Lane 2-4 Day 0, Lane 5-7 4h, Lane 8-10 Day 1, Lane 11-13 Day 2
std 2 3 4 5 6 7 8 9 10 11 12 13
0' 24h 48h 0' 24h 48h 0' 24h 48h 0' 24h 48h
U-STAT1
Actin

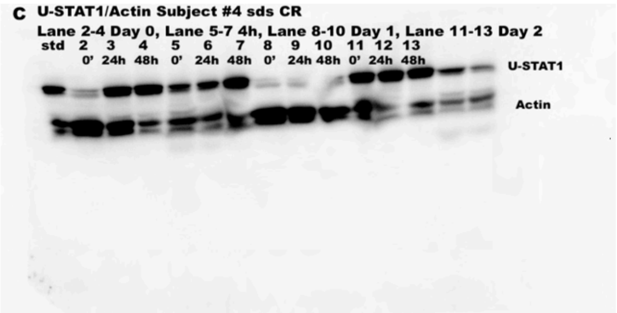
C
U-STAT1/Actin Subject #4 sds CR
Lane 2-4 Day 0, Lane 5-7 4h, Lane 8-10 Day 1, Lane 11-13 Day 2
std 2 3 4 5 6 7 8 9 10 11 12 13
0' 24h 48h 0' 24h 48h 0' 24h 48h 0' 24h 48h
U-STAT1
Actin

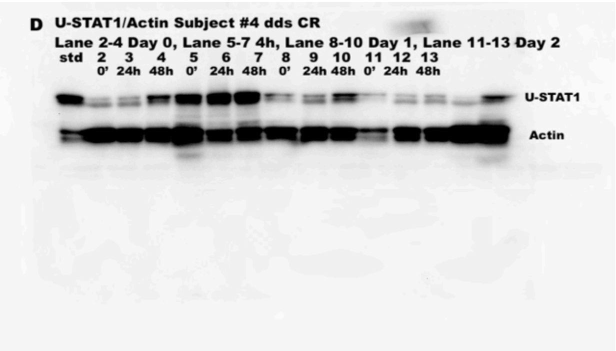
D
U-STAT1/Actin Subject #4 dds CR
Lane 2-4 Day 0, Lane 5-7 4h, Lane 8-10 Day 1, Lane 11-13 Day 2
std 2 3 4 5 6 7 8 9 10 11 12 13
0' 24h 48h 0' 24h 48h 0' 24h 48h 0' 24h 48h
U-STAT1
Actin

Subject #5

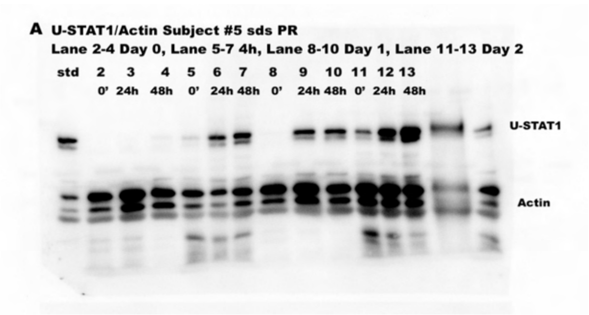
A
U-STAT1/Actin Subject #5 sds PR
Lane 2-4 Day 0, Lane 5-7 4h, Lane 8-10 Day 1, Lane 11-13 Day 2
std 2 3 4 5 6 7 8 9 10 11 12 13
0' 24h 48h 0' 24h 48h 0' 24h 48h 0' 24h 48h
U-STAT1
Actin

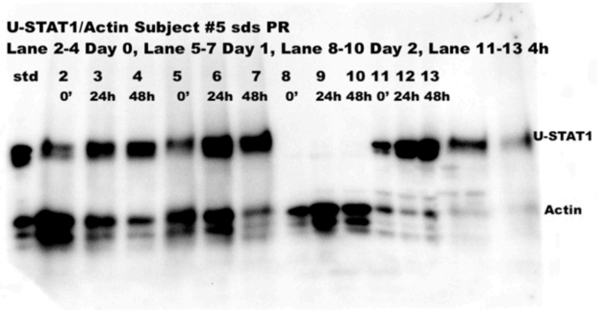
D
U-STAT1/Actin Subject #5 sds PR
Lane 2-4 Day 0, Lane 5-7 Day 1, Lane 8-10 Day 2, Lane 11-13 4h
std 2 3 4 5 6 7 8 9 10 11 12 13
0' 24h 48h 0' 24h 48h 0' 24h 48h 0' 24h 48h
U-STAT1
Actin

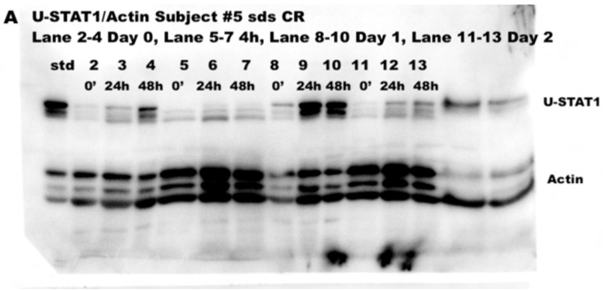
A
U-STAT1/Actin Subject #5 sds CR
Lane 2-4 Day 0, Lane 5-7 4h, Lane 8-10 Day 1, Lane 11-13 Day 2
std 2 3 4 5 6 7 8 9 10 11 12 13
0' 24h 48h 0' 24h 48h 0' 24h 48h 0' 24h 48h
U-STAT1
Actin

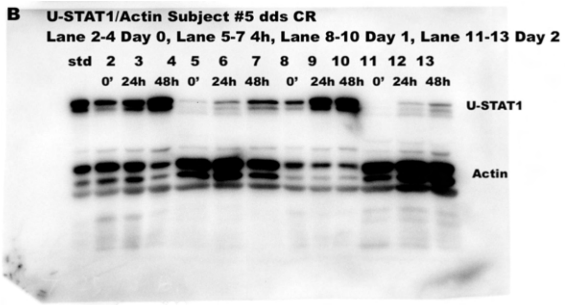
B
U-STAT1/Actin Subject #5 dds CR
Lane 2-4 Day 0, Lane 5-7 4h, Lane 8-10 Day 1, Lane 11-13 Day 2
std 2 3 4 5 6 7 8 9 10 11 12 13
0' 24h 48h 0' 24h 48h 0' 24h 48h 0' 24h 48h
U-STAT1
Actin

Subject #6

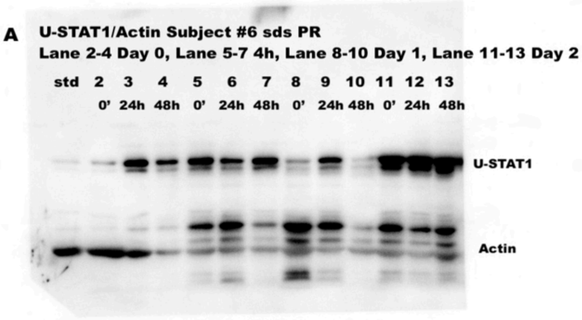

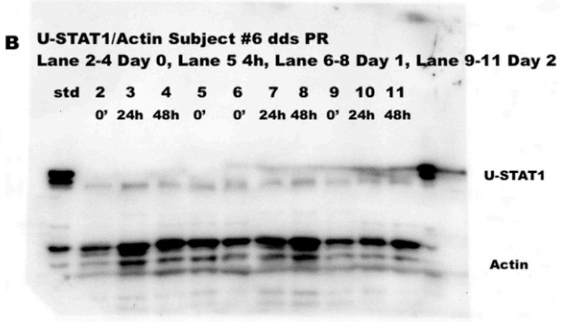

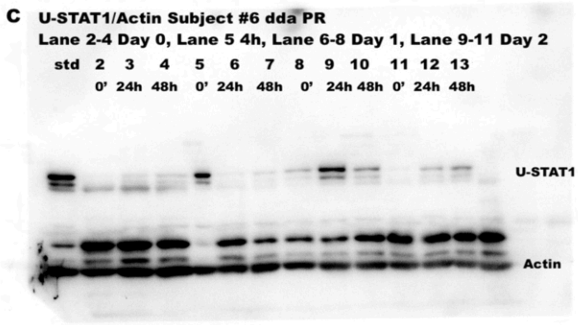

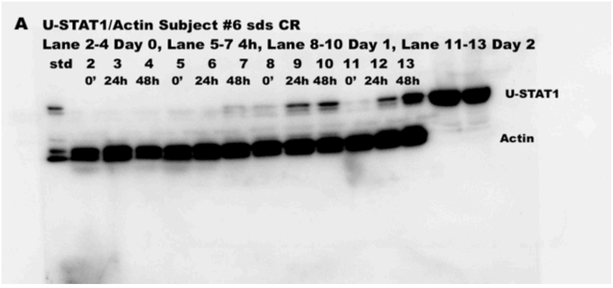

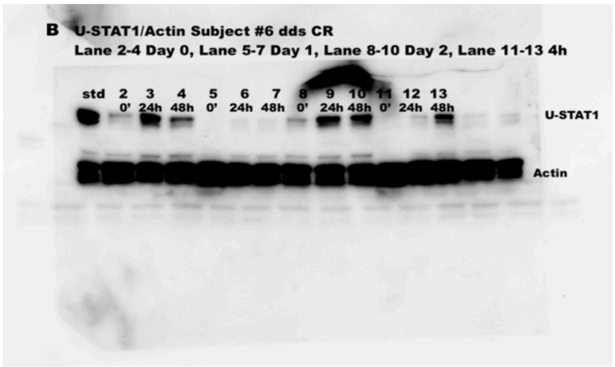

Subject #7

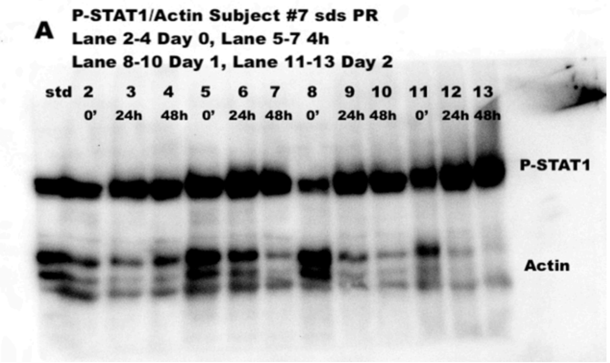

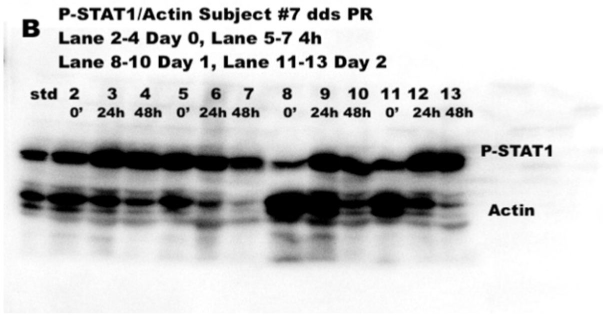

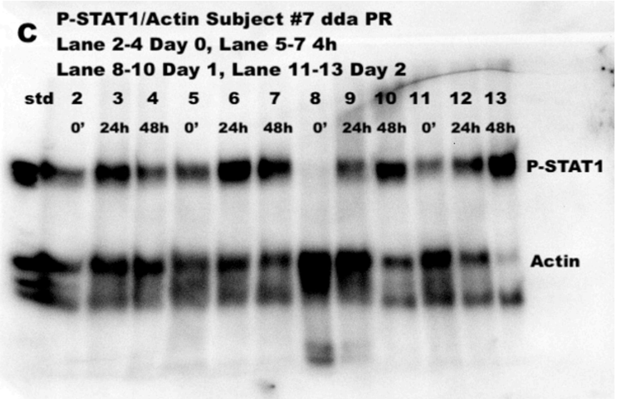
C
P-STAT1/Actin Subject #7 dda PR
Lane 2-4 Day 0, Lane 5-7 4h
Lane 8-10 Day 1, Lane 11-13 Day 2
std 2 3 4 5 6 7 8 9 10 11 12 13
0' 24h 48h 0' 24h 48h 0' 24h 48h 0' 24h 48h
P-STAT1
Actin

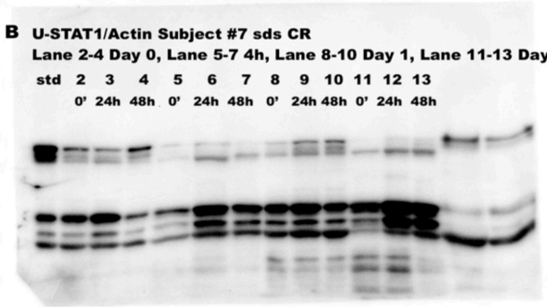
B
U-STAT1/Actin Subject #7 sds CR
Lane 2-4 Day 0, Lane 5-7 4h, Lane 8-10 Day 1, Lane 11-13 Day 2
std 2 3 4 5 6 7 8 9 10 11 12 13
0' 24h 48h 0' 24h 48h 0' 24h 48h 0' 24h 48h
U-STAT1
Actin

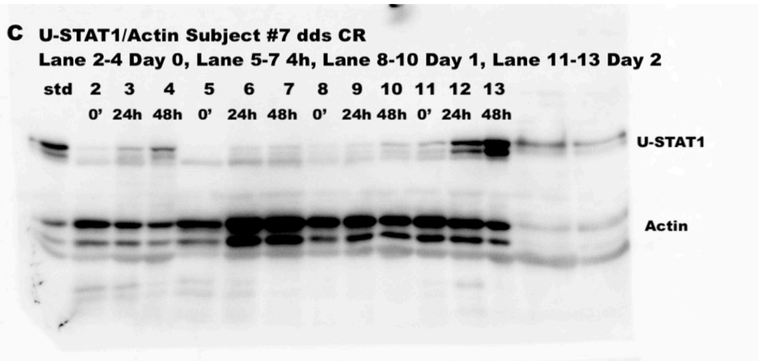
C
U-STAT1/Actin Subject #7 dds CR
Lane 2-4 Day 0, Lane 5-7 4h, Lane 8-10 Day 1, Lane 11-13 Day 2
std 2 3 4 5 6 7 8 9 10 11 12 13
0' 24h 48h 0' 24h 48h 0' 24h 48h 0' 24h 48h
U-STAT1
Actin

Subject #8

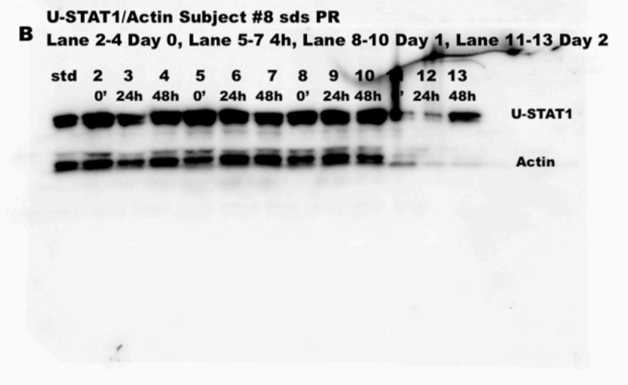
B
U-STAT1/Actin Subject #8 sds PR
Lane 2-4 Day 0, Lane 5-7 4h, Lane 8-10 Day 1, Lane 11-13 Day 2
std 2 3 4 5 6 7 8 9 10 12 13
0' 24h 48h 0' 24h 48h 0' 24h 48h 24h 48h
U-STAT1
Actin

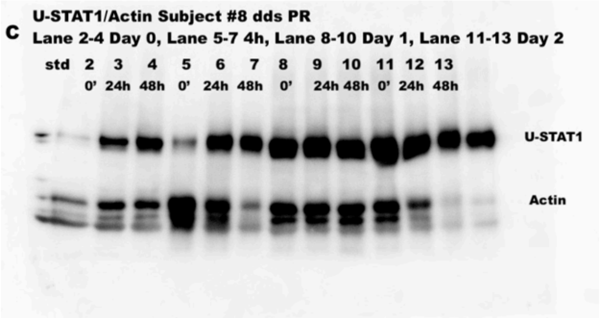
C
U-STAT1/Actin Subject #8 dds PR
Lane 2-4 Day 0, Lane 5-7 4h, Lane 8-10 Day 1, Lane 11-13 Day 2
std 2 3 4 5 6 7 8 9 10 11 12 13
0' 24h 48h 0' 24h 48h 0' 24h 48h 0' 24h 48h
U-STAT1
Actin

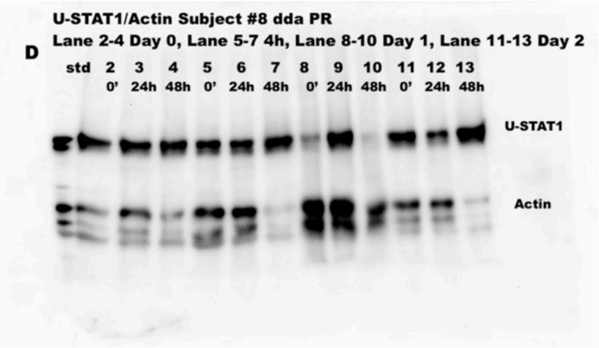
D
U-STAT1/Actin Subject #8 dda PR
Lane 2-4 Day 0, Lane 5-7 4h, Lane 8-10 Day 1, Lane 11-13 Day 2
std 2 3 4 5 6 7 8 9 10 11 12 13
0' 24h 48h 0' 24h 48h 0' 24h 48h 0' 24h 48h
U-STAT1
Actin

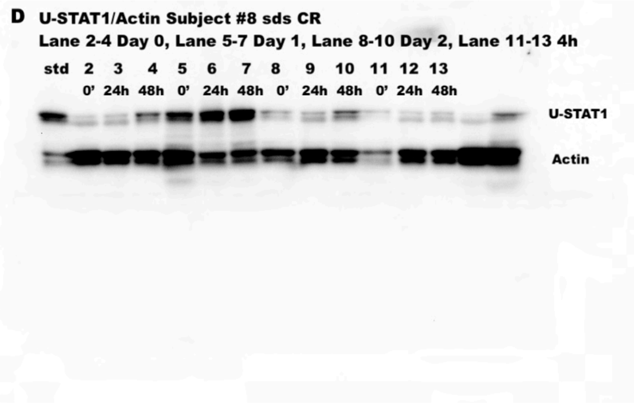
D
U-STAT1/Actin Subject #8 sds CR
Lane 2-4 Day 0, Lane 5-7 Day 1, Lane 8-10 Day 2, Lane 11-13 4h
std 2 3 4 5 6 7 8 9 10 11 12 13
0' 24h 48h 0' 24h 48h 0' 24h 48h 0' 24h 48h
U-STAT1
Actin

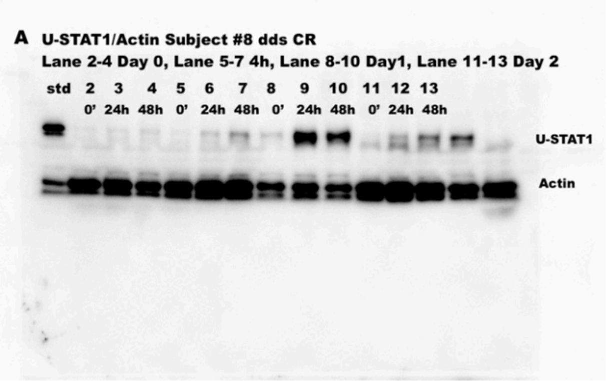

Subject #9

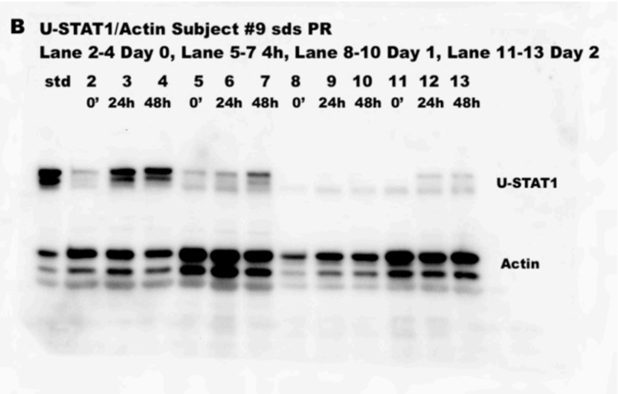

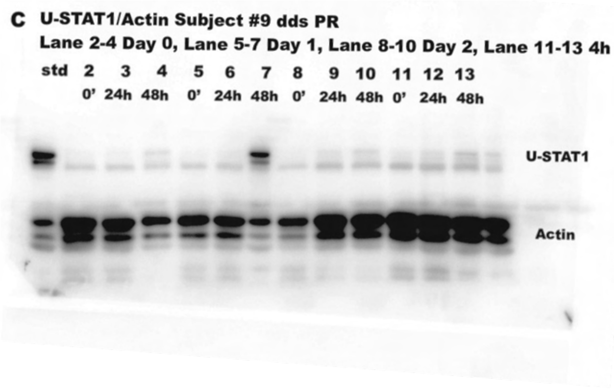

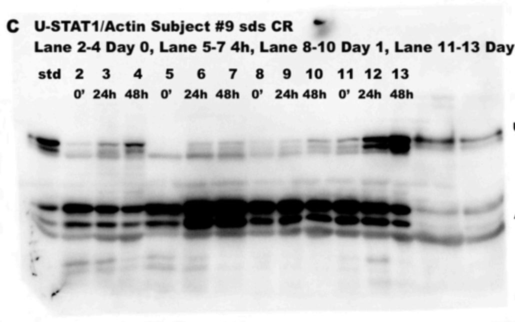

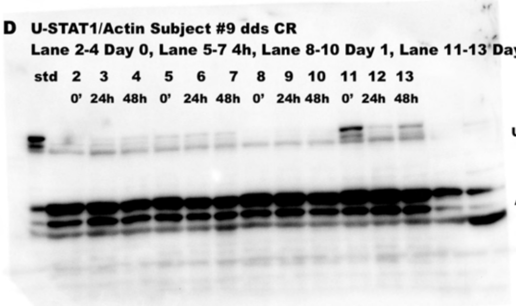

Subject #10

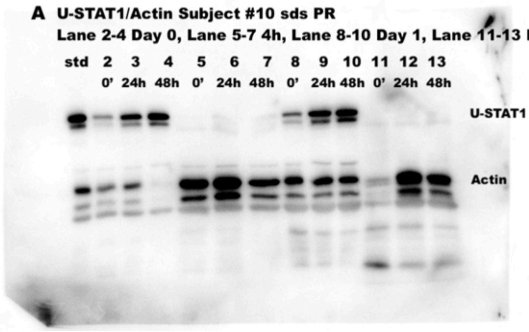

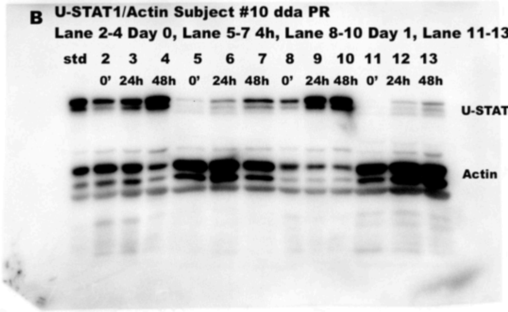

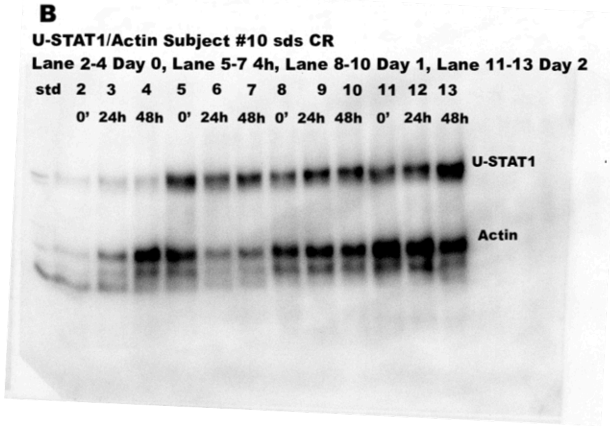
B
U-STAT1/Actin Subject #10 sds CR
Lane 2-4 Day 0, Lane 5-7 4h, Lane 8-10 Day 1, Lane 11-13 Day 2
std 2 3 4 5 6 7 8 9 10 11 12 13
0' 24h 48h 0' 24h 48h 0' 24h 48h 0' 24h 48h
U-STAT1
Actin

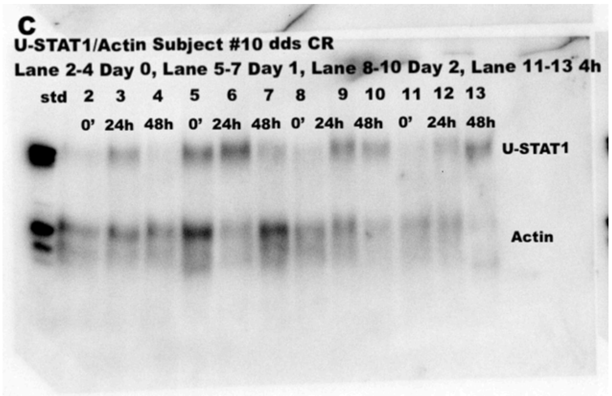
C
U-STAT1/Actin Subject #10 dds CR
Lane 2-4 Day 0, Lane 5-7 Day 1, Lane 8-10 Day 2, Lane 11-13 4h
std 2 3 4 5 6 7 8 9 10 11 12 13
0' 24h 48h 0' 24h 48h 0' 24h 48h 0' 24h 48h
U-STAT1
Actin

Subject #11

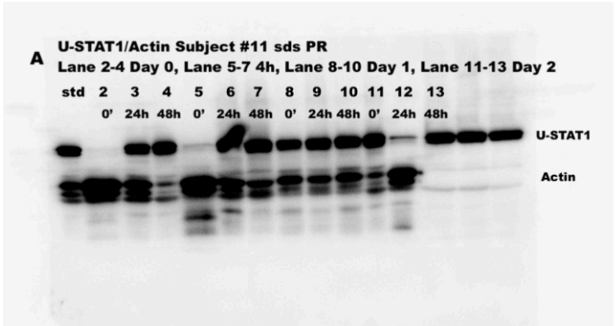
A
U-STAT1/Actin Subject #11 sds PR
Lane 2-4 Day 0, Lane 5-7 4h, Lane 8-10 Day 1, Lane 11-13 Day 2
std 2 3 4 5 6 7 8 9 10 11 12 13
0' 24h 48h 0' 24h 48h 0' 24h 48h 0' 24h 48h
U-STAT1
Actin

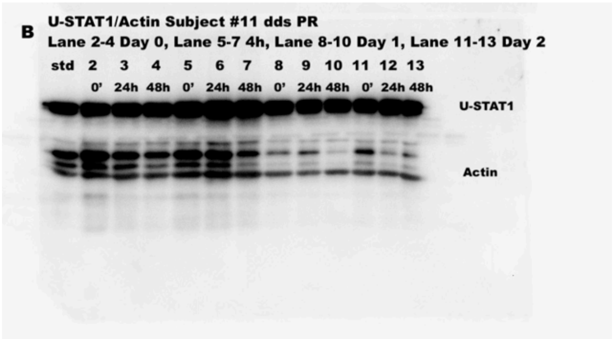
B
U-STAT1/Actin Subject #11 dds PR
Lane 2-4 Day 0, Lane 5-7 4h, Lane 8-10 Day 1, Lane 11-13 Day 2
std 2 3 4 5 6 7 8 9 10 11 12 13
0' 24h 48h 0' 24h 48h 0' 24h 48h 0' 24h 48h
U-STAT1
Actin

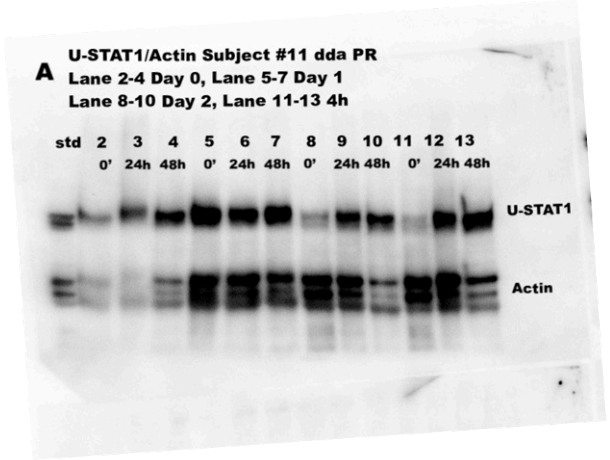
A
U-STAT1/Actin Subject #11 dda PR
Lane 2-4 Day 0, Lane 5-7 Day 1
Lane 8-10 Day 2, Lane 11-13 4h
std 2 3 4 5 6 7 8 9 10 11 12 13
0' 24h 48h 0' 24h 48h 0' 24h 48h 0' 24h 48h
U-STAT1
Actin

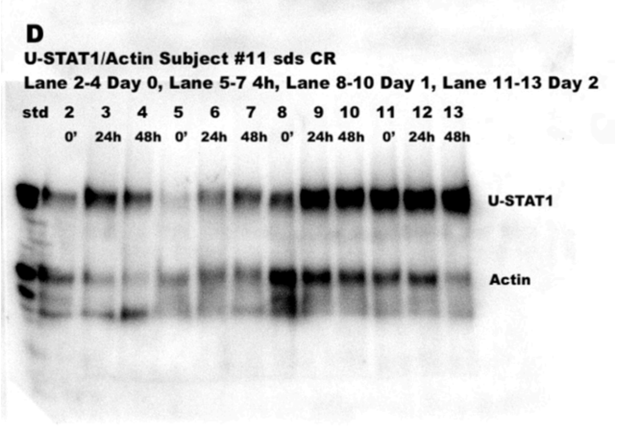
D
U-STAT1/Actin Subject #11 sds CR
Lane 2-4 Day 0, Lane 5-7 4h, Lane 8-10 Day 1, Lane 11-13 Day 2
std 2 3 4 5 6 7 8 9 10 11 12 13
0' 24h 48h 0' 24h 48h 0' 24h 48h 0' 24h 48h
U-STAT1
Actin

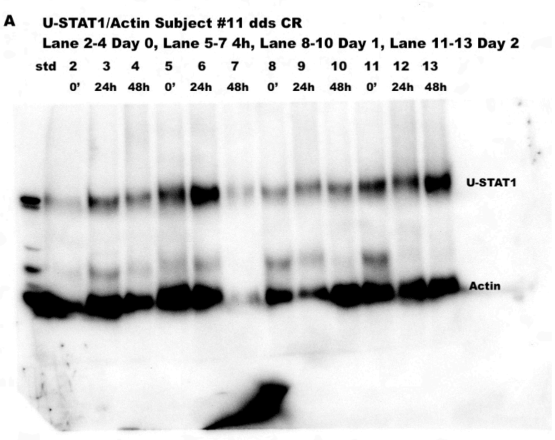

Subject #12

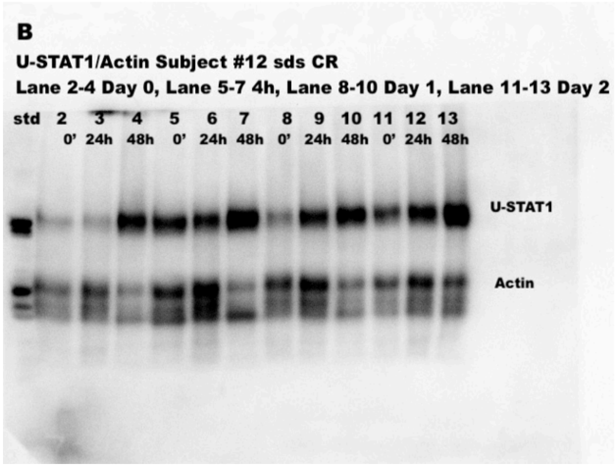

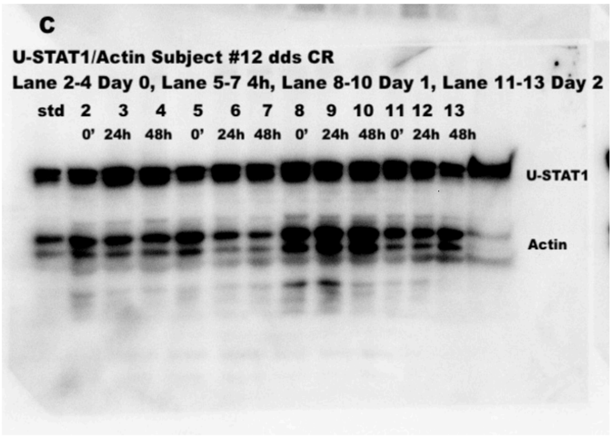

Subject #13

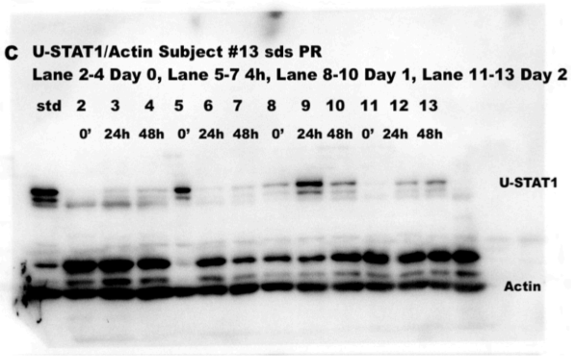

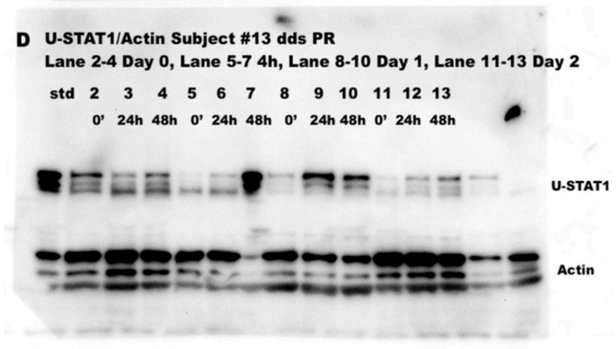

Subject #14

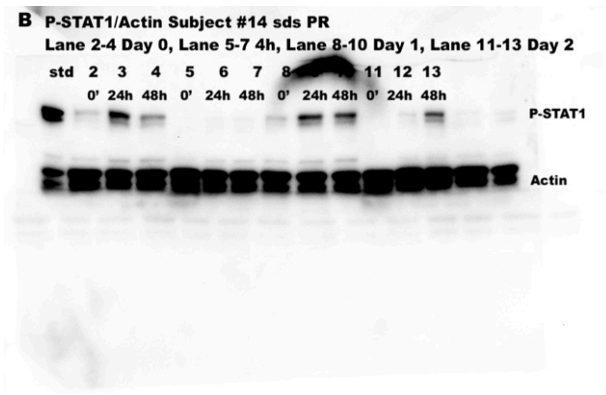

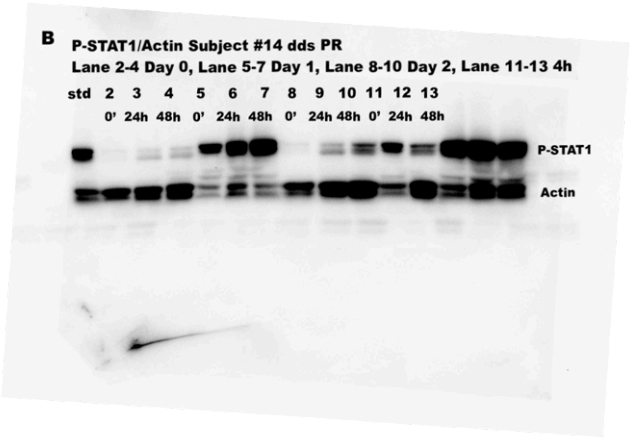

LSB = Laemmli Sample Buffer Only
Blots correspond to S1 table
