# Supplementary material for: IFN-β therapy rescues dysregulated IFN-stimulated proteins, serum cytokines, and neurotrophic factors in multiple sclerosis: Multiplex analysis of short-term and long-term IFN responses
Source: PLoS One. 2025 Sep 19;20(9):e0330867. doi: 10.1371/journal.pone.0330867 (PMC12449033; doi:10.1371/journal.pone.0330867)
Supplement: Attachment — Submitted filename: Review of PLOS Reder paper.pdf [file pone.0330867.s014.pdf]

Review of PLOS 24-55-183

This was a very difficult paper to read and follow. This is a follow-up paper from the EBioMed paper in 2019. These investigators are continuing work on samples obtained more than 10 years ago in a study of the effects in vivo and in vitro of beta-interferon, in MS patients. The samples were collected in a study that ended in 2012. When were these assays done? If done recently, how were the samples maintained during the years after the study?

The MS patients are divided into people on beta-interferon who are stable over approx 5 years, while another group had a relapse during BIFN treatment. Some samples also taken during a relapse. The study appears to have been well-planned and carried out.

Findings were difficult to follow, as it involves tables of numerical data, which are hard to read even with color coding. Graphic representation of the study design was greatly appreciated. Graphic representations of the results would be helpful too (e.g. longitudinal changes in the PR vs CR groups, including SEM bars to demonstrate overlap between the groups over time). An overall graphic summary of what the Investigators believe is happening, and the key differences between CR and PR would very much improve reader understanding.

Many different comparisons between IFNB use and dose with transcription factors, neurotrophins, cytokines, and chemokines were made. The Statistical handling of these multiple comparisons seems insufficient, with the use of primarily parametric t-tests or Mann-Whitney U tests. This reviewer would have expected more corrections for these multiple comparisons, or discussion of why they were not done. The potential for identifying spurious associations may have been compounded by the one-sided nature of some of the t tests, as well as the small-ish number of subjects. **It is this Reviewer's opinion that the advice of a statistician should be sought, and perhaps re-analysis of much or all of the data**.

In a few instances, the results in the PR and CR groups were at odds, for example MxA induction in vitro (Table 3c). Could this be spurious?

Interpretations regarding PR versus CR might also be spurious, and not (based on these data) able to be applied to individual patients. In almost all datasets, there was considerable overlap between the mean+/- SEM results in the two groups. Thus, the following Conclusion: "IFNB-induced proteins and IFN levels predict clinical response to IFNB" is overly zealous. It implies an ability to predict that is not clearly present in the results.

Vitamin D was quantitated, but it seemed "added on" and the rationale for examining it in this context was unclear until the Discussion (but even after reading, it still seemed added on). Recent studies do not support that high dose Vit D affects MS, making this part of the manuscript perhaps of little relevance. It was also odd that serum vitamin D correlated with serum cytokines in PR but not in CR, after IFNB injections. Could this be a spurious finding? Should this part be removed from the manuscript?

More of an explanation of why double dose IFNB was used is needed. This is not standard dose and why was it used in this study?

There were some statements in this paper that seemed stated as fact, but unproven. Here are some that seem overstated:

1. "….. virus infections in MS are half as frequent as in healthy controls [36]" Are there other references or studies beyond Ref 36?
2. Reference to " a <u>serum cytokine storm</u>" during MS exacerbations. This is not a commonly held concept for MS relapses, to Reviewer's knowledge. This should be toned down or even removed, or alternatively more data to support this statement added. The paper referenced is from the 1990's.
3. Description on Line 837 of NMO as being "an inflammatory CNS demyelinating disease" does not go far enough to describe the destructive nature of NMO. As these authors know well, NMO involves far more than inflammatory demyelination and it is likely that the demyelination is secondary."

A paragraph devoted to Study Limitations in Discussion is needed, but missing.

Otherwise, the Discussion seems too long.
